# Comparative analysis of structured RNAs in *S. cerevisiae *indicates a multitude of different functions

**DOI:** 10.1186/1741-7007-5-25

**Published:** 2007-06-18

**Authors:** Stephan Steigele, Wolfgang Huber, Claudia Stocsits, Peter F Stadler, Kay Nieselt

**Affiliations:** 1 Wilhelm-Schickard-Institut für Informatik, ZBIT-Center for Bioinformatics Tübingen, University of Tübingen, Sand-14, D-72076 Tübingen, Germany; 2 Bioinformatics Group, Department of Computer Science, and Interdisciplinary Center for Bioinformatics (IZBI), University of Leipzig, Härtelstraße 16-18, D-04107 Leipzig, Germany; 3 EMBL Outstation Hinxton, European Bioinformatics Institute, Wellcome Trust Genome Campus, Hinxton, Cambridge, CB10 1SD, UK; 4 Department of Theoretical Chemistry University of Vienna, Währingerstraße 17, A-1090 Wien, Austria; 5 Santa Fe Institute, 1399 Hyde Park Rd., Santa Fe, NM 87501, USA

## Abstract

**Background:**

Non-coding RNAs (ncRNAs) are an emerging focus for both computational analysis and experimental research, resulting in a growing number of novel, non-protein coding transcripts with often unknown functions. Whole genome screens in higher eukaryotes, for example, provided evidence for a surprisingly large number of ncRNAs. To supplement these searches, we performed a computational analysis of seven yeast species and searched for new ncRNAs and RNA motifs.

**Results:**

A comparative analysis of the genomes of seven yeast species yielded roughly 2800 genomic loci that showed the hallmarks of evolutionary conserved RNA secondary structures. A total of 74% of these regions overlapped with annotated non-coding or coding genes in yeast. Coding sequences that carry predicted structured RNA elements belong to a limited number of groups with common functions, suggesting that these RNA elements are involved in post-transcriptional regulation and/or cellular localization. About 700 conserved RNA structures were found outside annotated coding sequences and known ncRNA genes. Many of these predicted elements overlapped with UTR regions of particular classes of protein coding genes. In addition, a number of RNA elements overlapped with previously characterized antisense transcripts. Transcription of about 120 predicted elements located in promoter regions and other, previously un-annotated, intergenic regions was supported by tiling array experiments, ESTs, or SAGE data.

**Conclusion:**

Our computational predictions strongly suggest that yeasts harbor a substantial pool of several hundred novel ncRNAs. In addition, we describe a large number of RNA structures in coding sequences and also within antisense transcripts that were previously characterized using tiling arrays.

## Background

The genomic structure of yeast is much simpler than the genomic organization of multicellular species. With a size of about 12 million bases, the yeast genome is shorter than the genomes of most other currently known fungi; *Neurospora crassa*, as well as many other multicellular fungi, have up to 10 times larger genomes [1]. The genomic organization of yeast is also much simpler than that of its multicellular relatives. The yeast genome exhibits a rather straightforward pattern of coding genes with 5'-control (promoter) regions, usually intron-less coding-sequences (CDS), and very short 5'- and 3'-UTRs (untranslated regions) surrounding the coding sequences. The genome is densely packed with known genes, leaving only short intergenic sequences with a typical size of 300–600 bases [2].

Recent reports highlighted very different aspects of alternate regulative modes of gene expression in yeast. Several of them emphasize non-protein coding RNA molecules: the data in Steigele and Nieselt [3] showed an unexpected complexity of antisense transcripts, that could potentially bypass or supplement classical gene regulation. Havilio et al [4] analyzed protein coding regions in the *S. cerevisiae *genome. A substantial number of these sequences have no apparent orthologs in other species. Nevertheless, Havilio et al demonstrated abundant transcription of many of these orphan transcripts. A plausible working hypothesis is that most of these sequences are in fact non-coding RNAs (ncRNAs) similar to mRNA-like ncRNAs [5,6] that were erroneously annotated as protein coding genes.

Recent tiling array experiments [7-9] revealed abundant transcription of intergenic regions. In total, at least 80% of the yeast genome shows evidence of transcription. These observations emphasize the need for a concise computational analysis of non-coding RNAs in yeast, and for a comparison of those elements with verified transcripts of recent large-scale experiments.

Previously, only one computational study has been conducted to discover new ncRNAs in yeast [10]. This work focused on small ncRNA genes only, disregarding all structures that overlap with known features such as coding-sequences and UTRs. Several authors have pointed out, however, that structured RNAs might also be abundant in UTRs [11,12] as well as in protein coding regions [13-15]. Therefore, we consider here the entire yeast genome using RNAz [12], a comparative method for the *de novo *identification of structured RNAs. Structured RNAs are defined here to be either an ncRNA gene, or a conserved RNA structure embedded within coding sequences or UTRs. A detailed comparison of the predicted RNAs is provided, with experimental evidence from recent high-throughput experiments.

## Results

### A large number of structured RNAs in the yeast genome

We screened the genomes of the seven yeast species *S. cerevisiae*, *S. paradoxus*, *S. mikatae*, *S. kudriavzeii*, *S. bayanus*, *S. castelli *and *S. kluyveri *for structured RNAs. The coverage of the multiz multiple sequence alignments (MSAs) was almost complete, covering 96.7% of the 12 Mb yeast genome. This input data set consisted of 27031 individual alignment blocks longer than 20 bp that were processed in overlapping windows. Altogether, 239313 windows were analyzed, as described in the Methods section.

Washietl et al [12] showed that an RNA classification confidence value (*P*_*SVM*_) larger than 0.5 presents a plausible trade-off between specificity and sensitivity for most classes of non-coding RNAs. Thus, we used this *P*_*SVM *_value as the lower cutoff value. In addition, we report the data for a more conservative *P*_*SVM *_cutoff of 0.9. With a *P*_*SVM *_value larger than 0.5, 4567 windows with an RNA structure were found. Of these, 1821 windows have a *P*_*SVM *_value larger than 0.9. To remove false positives, we shuffled the alignments of all windows with a structured RNA and recalculated the probability of the shuffled alignment to contain a structured RNA. To be conservative, we removed predictions for which the shuffled alignments were also classified as structured RNAs with an above-cutoff classification confidence. This filtering step, indicated by a * in the following, retained 4395 candidates at PSVM∗
 MathType@MTEF@5@5@+=feaafiart1ev1aaatCvAUfKttLearuWrP9MDH5MBPbIqV92AaeXatLxBI9gBaebbnrfifHhDYfgasaacH8akY=wiFfYdH8Gipec8Eeeu0xXdbba9frFj0=OqFfea0dXdd9vqai=hGuQ8kuc9pgc9s8qqaq=dirpe0xb9q8qiLsFr0=vr0=vr0dc8meaabaqaciaacaGaaeqabaqabeGadaaakeaacqWGqbaudaqhaaWcbaGaem4uamLaemOvayLaemyta0eabaGaey4fIOcaaaaa@3278@ ≥ 0.5 and 1766 predictions at PSVM∗
 MathType@MTEF@5@5@+=feaafiart1ev1aaatCvAUfKttLearuWrP9MDH5MBPbIqV92AaeXatLxBI9gBaebbnrfifHhDYfgasaacH8akY=wiFfYdH8Gipec8Eeeu0xXdbba9frFj0=OqFfea0dXdd9vqai=hGuQ8kuc9pgc9s8qqaq=dirpe0xb9q8qiLsFr0=vr0=vr0dc8meaabaqaciaacaGaaeqabaqabeGadaaakeaacqWGqbaudaqhaaWcbaGaem4uamLaemOvayLaemyta0eabaGaey4fIOcaaaaa@3278@ cutoff 0.9. Overall, 3–4% of the positively predicted windows were identified as likely false positives in the shuffling experiment. Most of the removed candidates have very high sequence identity (91% versus an average of 79% in all predictions), so that there is little evidence from sequence covariation in these alignments. However, two classes of well known ncRNAs, rRNAs and tRNAs, also belong to this class of highly conserved sequence windows. In fact, sequence divergence of these RNA classes was much smaller than in protein coding regions. Correspondingly, 17.3% and 12.8% of them were removed in the shuffling step, indicating that the filtering step is too conservative at the highest levels of sequence conservation. All retained windows that were overlapping or that were at most 60 bp apart were combined into a single entity. From the 0.5 and 0.9 PSVM∗
 MathType@MTEF@5@5@+=feaafiart1ev1aaatCvAUfKttLearuWrP9MDH5MBPbIqV92AaeXatLxBI9gBaebbnrfifHhDYfgasaacH8akY=wiFfYdH8Gipec8Eeeu0xXdbba9frFj0=OqFfea0dXdd9vqai=hGuQ8kuc9pgc9s8qqaq=dirpe0xb9q8qiLsFr0=vr0=vr0dc8meaabaqaciaacaGaaeqabaqabeGadaaakeaacqWGqbaudaqhaaWcbaGaem4uamLaemOvayLaemyta0eabaGaey4fIOcaaaaa@3278@ values, we thus obtained 2811 and 1156 entities, respectively, that we refer to as 'predicted RNA elements' (see Additional file 1).

### Most predicted RNA structures overlap with genomic loci with known annotations

In order to assess the sensitivity of our screen, we compared our predictions with the *Saccharomyces *Genome Database (SGD), which provides an almost complete annotation of the yeast genome. We analyzed all features of the yeast genome that are related to the transcriptional output of the yeast genome and further subdivided these into several classes, including ncRNA (snoRNA, tRNAs, rRNA and snRNA) and several types of features that are related to proteins or more generally to mRNAs (CDS, pseudogenes, introns and transposable elements). A total of 2089 of 2811 (74%) and 789 of 1136 (69%) 'predicted RNA elements' from the 0.5 and 0.9 PSVM∗
 MathType@MTEF@5@5@+=feaafiart1ev1aaatCvAUfKttLearuWrP9MDH5MBPbIqV92AaeXatLxBI9gBaebbnrfifHhDYfgasaacH8akY=wiFfYdH8Gipec8Eeeu0xXdbba9frFj0=OqFfea0dXdd9vqai=hGuQ8kuc9pgc9s8qqaq=dirpe0xb9q8qiLsFr0=vr0=vr0dc8meaabaqaciaacaGaaeqabaqabeGadaaakeaacqWGqbaudaqhaaWcbaGaem4uamLaemOvayLaemyta0eabaGaey4fIOcaaaaa@3278@ value cutoff-level, respectively, overlap with a known feature of the yeast genome. The remaining RNA structures (722 (26%) and 347 (31%), respectively) did not significantly overlap with any annotated loci. In addition to the P-value, which was used as cutoff-value, we also computed the distribution of z-scores of predicted RNA structures as reported by RNAz for each annotation class (see Additional file 2).

**Figure 1:**
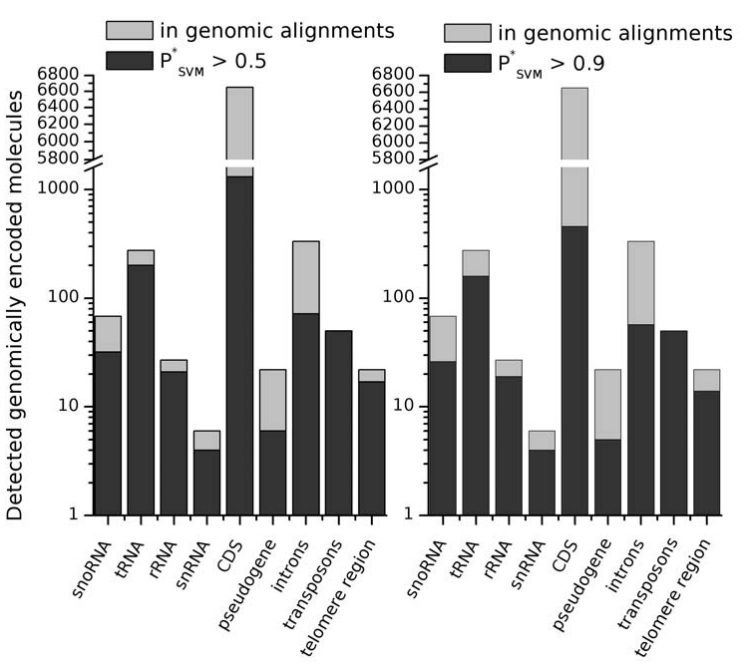
Known RNA genes in the yeast genome, covered by predicted RNA structures. The annotation was taken from the *Saccharomyces *Genome Database. Structured elements with reported PSVM∗
 MathType@MTEF@5@5@+=feaafiart1ev1aaatCvAUfKttLearuWrP9MDH5MBPbIqV92AaeXatLxBI9gBaebbnrfifHhDYfgasaacH8akY=wiFfYdH8Gipec8Eeeu0xXdbba9frFj0=OqFfea0dXdd9vqai=hGuQ8kuc9pgc9s8qqaq=dirpe0xb9q8qiLsFr0=vr0=vr0dc8meaabaqaciaacaGaaeqabaqabeGadaaakeaacqWGqbaudaqhaaWcbaGaem4uamLaemOvayLaemyta0eabaGaey4fIOcaaaaa@3278@ values larger than 0.5 (left) and 0.9 (right) are shown.

We found the majority of all known ncRNAs overlapped with 'predicted RNA elements' (Figure 1, and Additional files 3, 4). Conserved classes of ncRNAs were almost completely recovered by this screen: of 274 tRNAs, which are present in the input alignments (of a total of 299 annotated in the yeast genome), we recovered 227. About 12% of them were dropped in the filtering step at the 0.5 *P*_*SVM *_value cutoff-level, however. We almost completely recovered the ribosomal RNAs, which are encoded by the RDN1 and RDN2 loci.

In contrast to the strong and stable RNAz signals of the known ncRNA genes, the signals of predictions in the coding sequences are systematically weaker and are less likely to be destroyed by the shuffling procedure: only 2.4% of shuffled windows were again classified as 'structured RNA' compared to 3.8% of the entire screen. However, the majority of the predicted signals within the coding sequence vanished when they were filtered at the more restrictive 0.9 PSVM∗
 MathType@MTEF@5@5@+=feaafiart1ev1aaatCvAUfKttLearuWrP9MDH5MBPbIqV92AaeXatLxBI9gBaebbnrfifHhDYfgasaacH8akY=wiFfYdH8Gipec8Eeeu0xXdbba9frFj0=OqFfea0dXdd9vqai=hGuQ8kuc9pgc9s8qqaq=dirpe0xb9q8qiLsFr0=vr0=vr0dc8meaabaqaciaacaGaaeqabaqabeGadaaakeaacqWGqbaudaqhaaWcbaGaem4uamLaemOvayLaemyta0eabaGaey4fIOcaaaaa@3278@ value cutoff level. This effect is not simply explained by a higher mean sequence identity of coding sequences, because many classes of ncRNAs, in particular tRNAs and rRNAs, are much less variable than the coding sequences (see Additional file 3).

To evaluate the sensitivity of the screen, we defined the sensitivity as the proportion of correctly predicted RNA genes (TP) divided by the number of known RNA genes (T), i.e. *S*_*E *_= *TP*/*T*. The sensitivity of the genome-wide screen is the composite of two effects, namely the sensitivity of the RNAz classificator and the quality of the input alignments. In order to assess the latter contribution, we counted the total number of all known RNA genes that are represented in the input alignments. Almost all ncRNA genes reported in *S. cerevisiae *are present in the other yeast genomes and are also present in the multiple alignments. We concluded that the sensitivity of our screen is thus dominated by RNAz. For rRNAs and tRNAs we found *S*_*E*_(*rRNA*) = 0.78 and *S*_*E*_(*tRNA*) = 0.72, respectively, while we detected essentially all the transposable elements. Altogether, we predicted 257 out of 375 known ncRNAs, yielding a sensitivity of 69%.

### Structured RNAs associated with protein coding sequences

Altogether, we found 1309 coding sequences in *S. cerevisiae *that contained at least one structured RNA predicted by RNAz (Figure 1). Because of the general lack of a systematic analysis of structured RNAs in CDS regions, and in order to assess the false discovery rate in coding sequences (for which RNAz is not explicitly trained), we decided to re-evaluate the predictions of RNA structures found in the CDS more carefully.

The idea was firstly, to include a wider range of species in the search of conserved structures in coding-sequences to counterbalance the higher average sequence similarity in coding regions, and secondly, to employ a refined alignment and shuffling procedure that corrects specifically for potential biases arising from the special structure of coding sequences (see Methods). To ensure that highly similar sequences were not dominating the alignments, we always chose the four most diverged sequences. This is especially useful in sequence-based comparisons of coding-sequences that mutate much more slowly than sequences of ncRNAs and are therefore much more similar. Also, to achieve a high sequence diversity we used additional yeast species for the analysis that are more distant to *S. cerevisiae*. For the search of orthologs the following species were used: *S. kudriavzeii*, *S. mikatae*, *S. kluyveri*, *S. paradoxus*, *S. castelli*, *S. bayanus*, *A. gossypii *and *S. pombe*.

As a first step, we searched for orthologous sequences of *S. cerevisiae *proteins. Of 1309 CDS, 318 have no ortholog or are duplicated in *S. cerevisiae *and were disregarded. The remaining 991 CDS were then re-screened using the 'shuffled-CDS method' with the following result: at the cutoff level of 0.5, 286 of 991 CDS were found to contain a predicted conserved RNA structure. At the nucleotide level, the average mean percent identity of the RNA structure positive alignments was 61.7% compared to 67.8% overall (see Additional file 5).

Next, we considered whether the 286 CDS harboring a conserved RNA structure had common functions. For these, we analyzed the CDS by means of the gene ontology [16] (see Methods). SGD provided gene ontology (GO) terms for 285 of these genes. Interestingly, we found several large groups with common functional annotations (Table 1). Most of the CDS are involved in metabolic functions. We found the largest group of CDS function within non-membrane-bound organelles, especially within the mitochondrion. Other significant GO groups are involved in the formation of the ribosome, and catabolic functions such as protein catabolism or asparagin or carbohydrate metabolism.

**Table 1:** Dominant functional GO-terms of CDS with a predicted RNA structure

**GO term**	**# of CDS**	***P*-value**
mRNA-binding (hnRNP) protein import into nucleus	6	6 × 10^-4^
Carbohydrate metabolism	21	7 × 10^-4^
Sporulation	13	8 × 10^-4^
Development	26	1 × 10^-3^
Regulation of metabolism	31	5 × 10^-3^
Nitrogen compound metabolism	19	7 × 10^-3^
Protein catabolism	13	2 × 10^-2^
Ribosome	23	3 × 10^-3^
Intracellular non-membrane-bound organelle	55	5 × 10^-2^

Only significant groups larger than five CDS are reported. The P-values of a set of GO annotated genes is determined for a set of genes against the background of all genes in the genome sharing the same GO annotation (see Methods).

**Figure 2:**
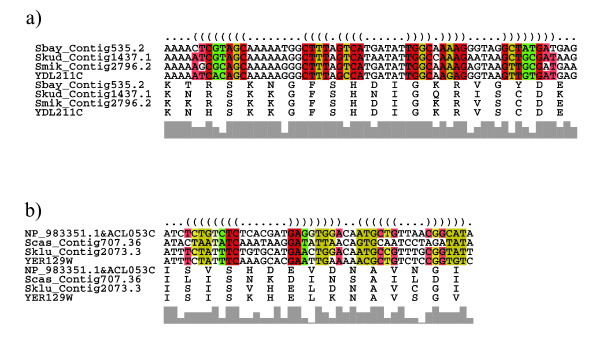
Examples of conserved RNA structures found within coding sequences with covariant sites caused by different substitutions of the corresponding amino acids. The color scheme used for coloring the alignment indicates the number of different types of base pairs that support stabilizing selection on the structure. The transparency indicates the number of incompatible pairs (ranging from 0 to 2 going from dark to light). Red: one base pair type; ochre: two base pair types; green: three base pair types. (A) Section of conserved structure found within an alignment of four fungal species (*S. bayanus*, *S. kudriavzeii*, *S. mikatae*, *S. cerevisiae*) of the yeast gene YDL211C, for which the protein product is vacuolar localized. Substitution of the amino acid arginine (R) by histidine (H) at the opening base pairs is followed by a different usage of codons for cysteine (C) in the respective closing base pairs. (B) Section of conserved structure found within an alignment of four fungal species (A. gossypii, S. castelli, S. kluyveri, S. cerevisiae) of the yeast gene SAK1 (YER129W) involved in glucose metabolism. Substitution of the amino acid isoleucine (I) by valine (V) at the opening base pairs is followed by a different usage of codons for glutamic acid (E) in the closing base pairs. Another example of covariant sites is within the second stem where a different usage of codons for asparagine (N) at the opening base pairs follows a substitution of isoleucine (I) to valine (V) in the closing base pairs.

At least some of the predicted RNA structures found within the CDS showed some covariant sites that lead to different substitutions of the corresponding amino acids. Two examples are given in Figure 2.

### Structured RNAs in UTRs of protein-coding genes

A group of predicted elements was found in the immediate vicinity of the protein coding sequences. In the case of yeast, most CDS unfortunately lack annotation of the exact transcript structure, so the exact positions of the 5'- and 3'-UTRs are unknown. We therefore pragmatically considered a window of 120 base pairs upstream and downstream of a CDS as a likely UTR. This approximation conforms with the approximation for UTR length given by Hurowitz et al [17]. We predicted 150 structured RNAs (at 0.5 PSVM∗
 MathType@MTEF@5@5@+=feaafiart1ev1aaatCvAUfKttLearuWrP9MDH5MBPbIqV92AaeXatLxBI9gBaebbnrfifHhDYfgasaacH8akY=wiFfYdH8Gipec8Eeeu0xXdbba9frFj0=OqFfea0dXdd9vqai=hGuQ8kuc9pgc9s8qqaq=dirpe0xb9q8qiLsFr0=vr0=vr0dc8meaabaqaciaacaGaaeqabaqabeGadaaakeaacqWGqbaudaqhaaWcbaGaem4uamLaemOvayLaemyta0eabaGaey4fIOcaaaaa@3278@ value cutoff level), which are approximately evenly distributed between 5'- and 3'-UTRs. Further details are shown in Table 2 (see also Additional file 6).

**Table 2:** Total number of putative UTRs containing structured RNAs

UTR	PSVM∗ MathType@MTEF@5@5@+=feaafiart1ev1aaatCvAUfKttLearuWrP9MDH5MBPbIqV92AaeXatLxBI9gBaebbnrfifHhDYfgasaacH8akY=wiFfYdH8Gipec8Eeeu0xXdbba9frFj0=OqFfea0dXdd9vqai=hGuQ8kuc9pgc9s8qqaq=dirpe0xb9q8qiLsFr0=vr0=vr0dc8meaabaqaciaacaGaaeqabaqabeGadaaakeaacqWGqbaudaqhaaWcbaGaem4uamLaemOvayLaemyta0eabaGaey4fIOcaaaaa@3278@ > 0.5		PSVM∗ MathType@MTEF@5@5@+=feaafiart1ev1aaatCvAUfKttLearuWrP9MDH5MBPbIqV92AaeXatLxBI9gBaebbnrfifHhDYfgasaacH8akY=wiFfYdH8Gipec8Eeeu0xXdbba9frFj0=OqFfea0dXdd9vqai=hGuQ8kuc9pgc9s8qqaq=dirpe0xb9q8qiLsFr0=vr0=vr0dc8meaabaqaciaacaGaaeqabaqabeGadaaakeaacqWGqbaudaqhaaWcbaGaem4uamLaemOvayLaemyta0eabaGaey4fIOcaaaaa@3278@ ≥ 0.9	
	Count	Distance	Count	Distance
5'-UTR	80	12.1 bp	33	13.5 bp
3'-UTR	87	8.6 bp	44	15.8 bp

Number of putative UTRs (120 bp 5' and 3', respectively, of a CDS) containing one or more structured RNAs. 'Distance' denotes the mean distance of predicted RNA structures from the CDS boundaries.

GO terms are available for 65 of the 80 CDS that have a predicted RNA element in their 5'-UTR. Here, we report selected significant groups larger than five CDS only. The most significant functional classes are development (8 genes, *P *= 1.3 × 10^-2^), regulation of cellular physiological processes (12 genes, *P *= 1.6 × 10^-2^), response to stress (9 genes, *P *= 2.2 × 10^-2^), a larger group of genes involved in the transport and localization of other proteins (15 genes, *P *= 4.0 × 10^-2^) and a group of genes involved in the cell cycle (8 genes, *P *= 4.6 × 10^-2^). A much large number of CDS with 5' structures are annotated constituents of non-membrane-bound organelle (47 CDS, *P *= 2.8 × 10^-3^). Here, the biggest subgroup consists of mitochondrial proteins. Around a quarter of all CDS with structured 5'-UTRs are related to mitochondrial function, homeostasis or integrity of mitochondria (19 CDS, *P *= 4.5 × 10^-3^).

Specific functional groupings are also found for the predicted 3'-UTR structures. GO terms are provided for 70 of the 87 CDS in question. Significant gene groups are involved in amino acid metabolism (8 genes, *P *= 7.0 × 10^-4^) or are constituents of the ribosome (11 genes, *P *= 1.8 × 10^-4^). Similar to CDS with RNA structures in their 5'-UTR, proteins were found that are constituents of non-membrane-bound organelles are again significantly overrepresented (19 CDS, *P *= 7.5 × 10^-3^).

**Figure 3:**
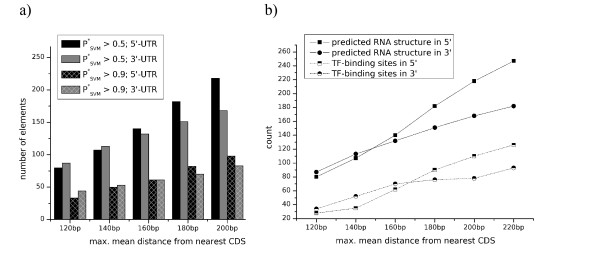
Correlation of 'predicted RNA structures' with UTR-regions and TF-binding sites. (a) Number of 'predicted RNA structures' in intervals of increasing length adjacent to coding sequences. (b) Number of 'predicted RNA structures' overlapping with transcription factor binding sites taken from [18].

Increasing the sequence intervals adjacent to a CDS should begin to cover elements that are independently transcribed. We therefore considered the distribution of RNAz hits in intervals with lengths increasing from 120 to 220 base pairs (Figure 3a). As expected, the number of positive predictions increases approximately linearly with interval length. Surprisingly, however, we found a strong bias towards structured RNAs at the 5'-side of the CDS. With increasing distance from the CDS boundaries, more RNA structure at the 5'- than the 3'-ends of the CDS was found. Recall that this bias is not present for the shortest interval, which essentially covers the UTRs.

A possible explanation for this is that many of these RNAz hits are associated with promoter regions. In order to investigate this possibility, we used the data on transcription factor binding sites in yeast that were compiled by Harbison et al [18](Figure 3b and Additional file 7). In addition, a higher number of TF-binding sites is covered by RNA structures on the 5'-end of CDS. The increased number of structures in the 5' region indeed strongly correlated with an increased number of overlapping TF-binding sites (Pearson correlation coefficients ρ_5' _= 0.99 and ρ_3' _= 0.98 for the 5' and 3' regions, respectively).

Interestingly, we found several transcription factor binding-sites that are frequently covered by predicted RNA structures (Table 3). With the exception of DIG1, these transcription factors are involved in stress response (CBF1 and MCM1) and/or the cell cycle (CBF1, MBP1, REB1 and SWI6).

**Table 3:** Transcription factor (TF)-binding sites overlapping with RNA structures

Binding site	CBF1	DIG1	MBP1	MCM1	REB1	SWI6
Total number of instances in genome	123	134	138	64	176	133
Number of instances covered by RNAz hits	22	17	32	28	10	35

Comparison of number of TF-binding sites of the six transcription factors CBF1 (cell cycle, stress response), DIG1 (Down-regulator of Invasive Growth), MBP1 (cell cycle), MCM1 (stress response), REB1 (cell cycle), SWI6 (cell cycle) overlapping with a predicted RNA structure with total number of TF-binding sites of these transcription factors. TF-binding site data were taken from [18].

### Novel ncRNAs in yeast

A total of 572 unannotated predicted RNA elements (at the 0.5 PSVM∗
 MathType@MTEF@5@5@+=feaafiart1ev1aaatCvAUfKttLearuWrP9MDH5MBPbIqV92AaeXatLxBI9gBaebbnrfifHhDYfgasaacH8akY=wiFfYdH8Gipec8Eeeu0xXdbba9frFj0=OqFfea0dXdd9vqai=hGuQ8kuc9pgc9s8qqaq=dirpe0xb9q8qiLsFr0=vr0=vr0dc8meaabaqaciaacaGaaeqabaqabeGadaaakeaacqWGqbaudaqhaaWcbaGaem4uamLaemOvayLaemyta0eabaGaey4fIOcaaaaa@3278@ cutoff level) are located in intergenic, non-UTR regions. The first question is if any of these elements are conserved outside of the hemiascomycetes. We therefore performed homology searches using blastn [19] with the following genomes: *Neurospora crassa*, *Cryptococcus neoformans*, *Aspergillus nidulans*, *Gibberella zeae *and *Ustilago maydis*. Only one significant hit was found, which is a short (and as yet unannotated) duplication of the 26S-RNA in vicinity to the original rRNA-cluster on chromosome 12 of *S. cerevisiae*. All other predicted elements seem to be restricted to the hemiascomycetes phylum.

We also searched specialized ncRNA databases to see if some of the 572 RNAz hits can be annotated by homology with a known functional ncRNA. A blast search (E-value 10^-6^) in the NONCODE database [20] revealed two significant hits. One element is the snoRNA *snR161 *that was recently identified by Schattner et al [21]. This sequence was not included in the release of the *Saccharomyces *Genome Database used in this work. The other element is 100% identical over a length of 80 nucleotides to an RNA from mice annotated as U5 RNA. However, intensive searches in mammalian genomes convinced us that this sequence is most likely a contamination and misclassified in NONCODE (NONCODE accession number u4120). Searches of the Rfam database [22] using Sean Eddy's Infernal software [23] did not provide additional annotation information.

The intergenic candidates were screened using snoGPS [21] and snoSCAN [24] for putative box H/ACA and box C/D snoRNAs, respectively. We found 5 box C/D candidates and 41 putative box H/ACA snoRNAs. The latter candidates have 58 putative uridylation targets in SSU or LSU rRNA. More than half of these target sites are also targeted by other, previously known snoRNAs. This high redundancy might explain why the deletion and/or depletion of many snoRNAs is not lethal [25,26]: there exists a functional backup within the genome. A list of snoRNA candidates and their predicted target sites is provided in Additional file 8.

Recently, several large scale-studies using yeast tiling arrays were published. David et al [9] used tiling arrays to determine the transcribed portion of the yeast genome. Samanta et al [7] and Davis et al [8] used tiling arrays to analyze the effect of deletions of essential RNA processing proteins on the yeast transcriptome. Taken together, these three studies provide evidence for approximately 650 transcribed genomic regions not covered by the SGD annotation.

**Figure 4:**
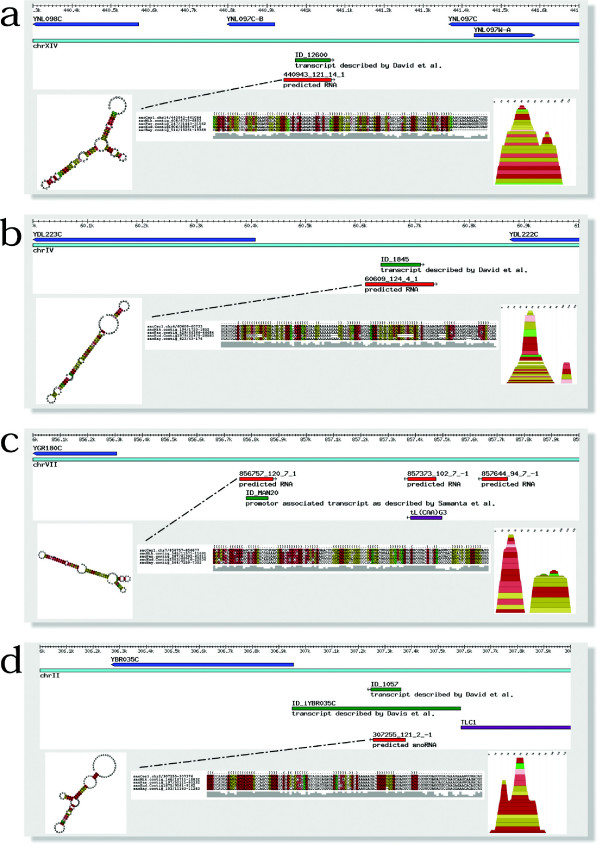
Examples of predicted ncRNAs: genomic context, tiling array pattern and predicted consensus structure. The color scheme used for coloring the RNA structures and the mountain plots representation is the same as in Figure 2. (a) and (b) Intergenic region with predicted RNA overlaps with transcripts described by David et al [9]. Note, that in (b) the sequence for sacKud.contig1979/20479-20583 is truncated (a stretch of seven gaps in the 3' end of the stem, which is not compensated by the deletions in the 5' part of the stem), which probably renders an unusable RNA due to an altered secondary structure. (c) Intergenic region with predicted RNA overlaps with promoter associated transcript described by Samanta et al [7]. (d) Predicted H/ACA snoRNA, overlapping with transcripts described by both David et al [7] and Davis et al [8].

In summary, transcription of 96 (16.8%) of the predicted intergenic RNA elements is verified by tiling array data, for additional 49 elements there is evidence from ESTs and/or SAGE data (Table 4, see also Additional file 9). Some prominent examples are shown in Figure 4.

**Table 4:** Comparison of predicted RNA elements with yeast tiling array data or SAGE/EST data

Experimental data	RNAz hits		intergenic transcripts		p-value
	RNAz hits	Intergenic regions	Transcripts	Intergenic regions	
Davis et al [8]	41	36	196	196	1 × 10^-7^
David et al [9]	40	36	372	294	9.4 × 10^-4^
Samanta et al [7]	15	12	77	74	5.8 × 10^-3^
Combined	84	72	573	536	-
ESTs [66]	17	15	154	116	1.8 × 10^-2^
SAGE [28]	32	31	680	533	0.91
Total combined	124	109	1202	1035	-

A predicted RNA structure is reported as transcribed if its overlap with an experimentally verified transcript is larger than 50%. RNAz hits shows the total number of intergenic RNAz hits that overlap a transcript. Intergenic regions describes the number of intergenic regions, that are covered by at least one predicted RNA structure. Intergenic transcripts shows the total number of intergenic transcripts. Intergenic regions depicts the number of intergenic regions that are covered by at least one transcript. All transcripts that collapse to one genomic locus were counted once in the rows combined and total combined. The numbers given in the intergenic regions column were used to calculate p-values (with a total of 6551 intergenic regions and 469 regions that are covered by at least one predicted RNA structure). The p-values are calculated for the one-sided hypergeometric test against the null hypothesis of odds-ratio ≤ 1.

Interestingly, intergenic transcripts seem to be enriched with RNA secondary structure (in the datasets of Davis et al, Samanta et al and David et al, 18.4%, 16.9% and 11.9%, respectively, contain a predicted RNA structure). Samanta et al further provided a sub-classification of intergenic transcripts into real intergenic transcripts and transcripts that are associated with known promoter regions. Interestingly, 13 of 15 RNA elements overlap with promoter-based transcripts (of a total of 50 promoter-based transcripts reported in [7]). However, there is little intersection between the individual transcript datasets: only eight RNA elements overlap with transcripts described by David et al and Davis et al, and four RNA elements with transcripts from David et al and Samanta et al. The predicted RNA elements overlapping with transcripts as predicted by the tiling arrays fall into at least two classes: most of our predicted RNA structures are smaller than the transcripts with which they are overlapping. One exception is a subset of transcripts described by David et al that were found using total RNA, where a large fraction of the transcripts (11 of 20) was of equal size or even smaller than the predicted RNA structure.

A similar number of the intergenic RNA structures were also verified by EST sequences. From the 154 ESTs that unambiguously map mainly to intergenic regions of the yeast genome, 33 ESTs overlap with 17 predicted (novel) ncRNAs. To check for typical signals of POL-II transcripts, we searched for poly-(A) tails using the program Trimest [27] (with standard parameters). Of the original 3041 EST sequences, Trimest predicted 197 EST sequences would contain poly-(A) tails. Three of these poly-(A) containing EST sequences (T37778, T37205, T38846) overlap with a predicted RNA structure.

In addition, the overlap of these sequences with 680 intergenic SAGE-tags [28] was analyzed. Here, 36 different tags overlapped with 32 predicted ncRNAs.

### Non-coding antisense transcripts

One question that arises when analyzing RNA structure elements is their overlap with known antisense transcripts. We compared predicted RNA elements with transcribed antisense sequences deduced from tiling array data [9]: 63 predicted RNA structures (PSVM∗
 MathType@MTEF@5@5@+=feaafiart1ev1aaatCvAUfKttLearuWrP9MDH5MBPbIqV92AaeXatLxBI9gBaebbnrfifHhDYfgasaacH8akY=wiFfYdH8Gipec8Eeeu0xXdbba9frFj0=OqFfea0dXdd9vqai=hGuQ8kuc9pgc9s8qqaq=dirpe0xb9q8qiLsFr0=vr0=vr0dc8meaabaqaciaacaGaaeqabaqabeGadaaakeaacqWGqbaudaqhaaWcbaGaem4uamLaemOvayLaemyta0eabaGaey4fIOcaaaaa@3278@ ≥ 0.5 cutoff level) that overlapped with antisense transcripts were found. It was shown previously that *S. cerevisiae *exhibits a large number of CDS that overlap as sense/antisense pairs [3]. Of these 369 *cis*-antisense pairs, 59 pairs (16%) have predicted structures in their overlap region. In addition, 27 intergenic RNA elements form large duplex regions, which potentially act as pure non-coding antisense transcripts (see Additional file 10, which shows the duplex structure of 16 pairs that are formed by the 27 elements).

## Discussion

The comparative search in several yeasts showed a large number of signals indicative for structured RNAs. We found evidence for structured RNAs not only in intergenic regions (that are often believed to be ncRNAs [10,29]), but also in coding regions and untranslated regions of coding sequences. The only previous *in silico *study to predict new ncRNAs in yeast by McCutcheon and Eddy [10] used QRNA [30] and was based on pairwise alignments of the intergenic regions only. The authors estimated the sensitivity of their screen to be 45%, measured against known and annotated ncRNAs (162 predicted out of 363 known ncRNAs). In contrast to the screen of McCutcheon and Eddy, we considered the entire genomic sequence. Based on multiple alignments instead of pairwise alignments, our RNAz-based approach has a significantly increased sensitivity and specificity. We recovered 257 of the 375 known ncRNAs in the *S. cerevisiae *genome, amounting to a sensitivity of 69%. We retrieved almost all known ncRNAs that were also detected by QRNA, while the overlap with the novel predictions is much smaller. Only 42 of the 94 candidate ncRNAs from McCutcheon and Eddy [10] are contained in our predictions. McCutcheon and Eddy verified the transcription of eight candidate ncRNAs (RUF1-8) using Northern blots; three of these (RUF4, RUF6, RUF7), however, turned out to be false positives in later experiments; RUF8 was identified as a misclassified ORF. Our RNAz-based approach classified RUF1, RUF2, RUF3, RUF5-1 and RUF5-2 as structured RNAs, but did not detect any of the false positives. This observation adds confidence to the specificity of our approach.

Surprisingly, the largest single class of predicted RNA structures was found in protein coding sequences. By contrast, it is widely believed that RNA structures in CDS can interfere both with translation and with the evolution of the protein coding sequence [13]. Furthermore, statistical evidence of widespread secondary structure in eukaryotic CDS was recently provided by Meyer et al [14]. The best-known examples of RNA structures that are superimposed on protein coding regions come from viruses: e.g. the Rev response element of HIV1 [31] or the cis-acting regulation element (CRE) in picorna viruses [32]. Eukaryotic examples are the mammalian steroid receptor activator (SRA) [33] or the plant gene ENOD40 [34]. An example in yeast is ASH1, which is one of the best-studied systems for localization of mRNAs within the cell [35,36]. The ASH1 mRNA harbours at least four regions (E1, E2A, E2B, E3) with RNA secondary structures within its protein coding region. These localization elements of ASH1 have no similarity on the sequence level, but are structurally related, thus, it is believed, that these elements function on the structural level [37]. Our data strongly suggest that this phenomenon is in fact common in yeast.

The relevance of the observation of a large number of structured RNA elements in coding regions is supported by an unexpected clustering of functional GO annotation terms of the affected protein coding genes. This significant clustering into a small number of functional classes strongly supports the interpretation that these RNAz hits are functional on a posttranscriptional level. The most prominent groups is related to cellular metabolism. Another large group of proteins is found to function within the ribosomal complex or within the mitochondria. ASH1 also belongs to the latter group. Many mitochondrial proteins are among the 55 organelle-specific proteins that have RNAz signals. This list includes in particular ATP2 and TIM44, both of which are known to be actively transported to the mitochondria [38,39]. It is tempting to speculate that many or most of RNA structures within coding sequences are functional as localization signals.

Structured RNA elements in UTR regions (*cis*-acting elements) often bind trans-acting factors and control important aspects of gene expression, such as translational efficiency, mRNA stability and subcellular localization. Known examples are iron response elements (IRE), the translation control elements (TCE), internal ribosome entry sites (IRES) and AU-rich elements [40-43]. In addition, many cellular targeting signals are located within UTRs [37]. From our screen, two groups of CDS with conserved RNA structures in their 3'-UTRs seem to be of special importance. First, one group of proteins is involved in the process of translation, mostly ribosomal proteins. Shalgi et al [44] also reported that genes with common RNA sequence motifs in their 3'-UTR that control the stability of the transcripts are enriched in ribosomal proteins. It is conceivable that similar RNA motifs are embedded in larger, conserved structured regions that can be detected by RNAz.

The second large group consists of mitochondrial genes with structured 3'-UTRs. A number of mRNAs corresponding to nuclear-encoded mitochondrial proteins are targeted to the vicinity of mitochondria [45,46,39,38]. Many of the *cis*-acting mitochondrial localization elements are localized in the 3'-UTRs of the transcripts and are shown to be sufficient to target mRNAs to mitochondria [39]. Together with the structured signals found in CDS of mitochondrial proteins, this is the first report of an enlarged set for this class of proteins. Shalgi et al [44] described a motif common to many mitochondrial proteins, which was also associated with a distinct subcellular localization. It is plausible that more nuclear encoded mitochondrial transcripts are actively transported. However, more subtle roles of transcript localization might exist that seem to be partially redundant, and where the specific localization mechanisms are not yet completely understood.

Most of the predicted RNA structures with a distance of more than 120 bp to the nearest known feature could not be reliably annotated. With a very small number of exceptions, no significant sequence or structural homology outside the *Saccharomyces *genus was found. Nevertheless, the combination of three independent tiling array studies, EST data, and SAGE data provide evidence that about 120 of these novel intergenic elements are transcribed in *S. cerevisiae*. As our computational approach is designed to detect stabilizing selection acting on the RNA structure, we suggest that these transcripts are functional at the RNA level rather than being the mere by-product of other regulatory processes or constituting transcriptional noise.

For a subclass of the novel intergenic elements, we have at least circumstantial evidence that hints at their function. Firstly, a significantly larger number of structured RNAs is predicted in the 5' vicinity of known protein coding transcripts than in their 3' neighborhood. Secondly, tiling array data indicate that many of the transcribed sequences are promoter associated transcripts in the sense that they are transcribed upstream of a gene and covered the promoter region of the gene. Structured RNA signals are overrepresented in these sequences. One of the current hypotheses about the function of promoter-associated transcripts suggests that these RNAs are directly involved in transcriptional regulation of Pol II due to occupied promoter regions [7]. Recently, such a regulation was shown in yeast for the ncRNA *SRG1*, which controls the transcription of its downstream gene *SER3 *[47,48].

Our data also suggest another possibility. Recently, Thomas et al [49] described a synthetic aptamer that binds with high affinity to Pol II and is able to specifically inhibit transcription. Similar cases are known for an ncRNA (B2) in mouse, that acts in the same way in response to stress signals [50,51], and the bacterial 6S RNA [52,53]. A non-coding RNA, *Evf-2*, that probably acts as a transcriptional enhancer, was recently found in mammals [54]. Most probably, these molecules are examples of an expanding repertoire of direct transcriptional modifiers. It is thus not implausible that many of the promoter-based transcripts that exhibit a conserved RNA structure function via direct modification of the Pol II transcription complex.

Finally, our data also indicate that at least some of the predicted structured RNAs could be functional by a direct modus via RNA-RNA interactions: we derived a substantial number of CDS/ncRNA or ncRNA/ncRNA antisense overlaps from the computational data, drawing a picture similar to that known in other eukaryotic species [55,56]. This finding further implies that the antisense mechanism is dependent on RNA structures, for example to control the accessibility of antisense regions in the first step of duplex formation.

## Conclusion

The comparative analysis of the genomes of seven yeast species to predict evolutionary conserved RNA secondary structures provided strong evidence for a large number of small ncRNA genes and structural motifs that overlap with known features such as coding seqences and UTRs. Altogether, we found roughly 2800 genomic loci that show conserved RNA secondary structures; many of these were ranked with high-scoring *P*-values, indicating several previously unknown ncRNAs. Furthermore, transcription of a number of predicted elements is supported by experimental data. Overall, although our findings are predictions, the present survey of evolutionary conserved structured RNA motifs in yeast genomes suggests widespread and diverse functions for structured RNAs in these organisms that we are only beginning to understand.

## Methods

### Data sources

Multiple alignments, calculated by the multiple alignment program multiz [57] of seven yeast species (*S. cerevisiae*, *S. paradoxus*, *S. mikatae*, *S. kudriavzeii*, *S. bayanus*, *S. castelli*, *S. kluyveri*) were downloaded from the Genome Browser at UCSC, California [58]. Each alignment includes the genomic sequences of *S. cerevisiae *as a reference, which is used for annotation of the alignments via known genetic elements from the genome of *S. cerevisiae*.

### Processing of multiple genome alignments

Genomic alignments were processed using the following protocol. In alignments with only two sequences, all gapped positions were deleted. In alignments with more than two sequences, all columns with more than 50% gap characters were removed. If the number of sequences in an alignment was larger than six sequences, one of the two most closely related sequences was removed. This is necessary as the machine-learning approach implemented in the RNAz program is not able to process alignments with more than six sequences. Final alignment sizes larger than 200 bp were processed by a sliding-window approach with a windows size of 120 bp and a stepsize of 40 bp.

### Detection of structured RNAs

We used RNAz v1.01 to predict structured RNAs. Both the forward and backward strand of the alignments were screened separately. The RNAz classifier is based on a support vector machine (SVM). This classifier computes a probability *P*_*SVM *_value that the input alignment has a significant evolutionary conserved secondary structure based on the thermodynamic stability of predicted structure and on sequence covariations consistent with a common structure. For details we refer to [12]. An RNA structure with a *P*_*SVM *_value of 1 defines the most reliably predicted RNA. Signals with a *P*_*SVM *_value smaller than 0.5 were discarded.

As the sensitivity of RNAz is dependent on base composition and sequence identity, we used a shuffling algorithm developed for ncRNAs [59] to remove alignments that also showed a significant RNA structure signal after shuffling. Therefore, all alignments that contained a predicted structured RNA with a *P*_*SVM *_value higher than 0.5 (0.9) were shuffled once and re-screened with RNAz. All alignments that had a *P*_*SVM *_value higher than 0.5 (0.9) after shuffling were discarded. RNAz also computes a z-score, which could be interpreted to quantify the thermodynamic stability of the predicted RNA structure versus the folding energy relative to a set of shuffled sequences.

Finally, all results of the RNAz screen and the corresponding alignments were stored in a relational database for further processing and analysis of the structured RNAs.

### Dynamic mapping of windows to corresponding genomic loci

All multiz alignments were fragmented during the RNAz screen. As we did not track all column removals, we needed to remap the positively classified alignment windows onto the *S. cerevisiae *genome. We used BLAT [60] for this purpose. In many cases, multiple BLAT hits with comparable scores were obtained. In these cases, we used the genomic location given in the multiz alignments and compared the new coordinates and chromosomal positions with the original coordinates. The best compatible coordinates with respect to the original coordinates were chosen.

### Construction of annotation elements

Overlapping windows and windows that are at most 60 bp apart were combined to '*predicted RNA elements*' and thus regarded as single entities. However, a '*predicted RNA element*' is a dynamic entity, which is dependent on construction rules: only windows above a certain treshold for the *P*_*SVM *_value are allowed. In fact, two different *P*_*SVM *_values for the filtering process were used. The first measurement is the *P*_*SVM *_value for the original alignment and the second measurement is the *P*_*SVM *_value for the shuffled windows, which is denoted as PSVM∗
 MathType@MTEF@5@5@+=feaafiart1ev1aaatCvAUfKttLearuWrP9MDH5MBPbIqV92AaeXatLxBI9gBaebbnrfifHhDYfgasaacH8akY=wiFfYdH8Gipec8Eeeu0xXdbba9frFj0=OqFfea0dXdd9vqai=hGuQ8kuc9pgc9s8qqaq=dirpe0xb9q8qiLsFr0=vr0=vr0dc8meaabaqaciaacaGaaeqabaqabeGadaaakeaacqWGqbaudaqhaaWcbaGaem4uamLaemOvayLaemyta0eabaGaey4fIOcaaaaa@3278@. We filtered windows with *P*_*SVM *_values lower than 0.5 and 0.9 and PSVM∗
 MathType@MTEF@5@5@+=feaafiart1ev1aaatCvAUfKttLearuWrP9MDH5MBPbIqV92AaeXatLxBI9gBaebbnrfifHhDYfgasaacH8akY=wiFfYdH8Gipec8Eeeu0xXdbba9frFj0=OqFfea0dXdd9vqai=hGuQ8kuc9pgc9s8qqaq=dirpe0xb9q8qiLsFr0=vr0=vr0dc8meaabaqaciaacaGaaeqabaqabeGadaaakeaacqWGqbaudaqhaaWcbaGaem4uamLaemOvayLaemyta0eabaGaey4fIOcaaaaa@3278@ -values higher than 0.5 and 0.9, respectively.

### Cross-annotation of known features via CHADO-based databases

Most of the annotation was performed using precalculated annotations from the *Saccharomyces Genome Database *[61]. We used a lightweight version of the *Saccharomyces Genome Database*, SGDlite, which has been implemented using the Generic Model Organism Database Construction Set as part of the GMOD project [62].

The genomic loci with RNAz predictions were compared with the SGD annotation. A 'predicted RNA element' was defined to overlap with an SGD annotation element if its sequence length overlaps at least 20% with the respective length of the SGD element.

### The shuffled-CDS method

To align protein coding sequences (CDS) at the level of nucleotide sequence, we aligned sequences in protein space and project the aligned positions back to the nucleotide coordinates. The resulting alignments have some characteristics that are different from pure nucleotide alignments, such as any gap position is a multiple of three (one codon). The background signal within coding regions thus has to be estimated from a random model that takes the protein coding nature of the sequence into account.

The first step of the shuffled-CDS method is the determination of a set of orthologous proteins. Orthology is determined by best-reciprocal FASTA [63] hits in a genome-wide comparison. The multiple alignment of the protein sequences is then backtranslated to nucleotide space. Next, a stepwise exclusion of the most similar sequences is performed until a user defined cutoff value (percent identity or number of sequences) is reached. The outcome of this step is a multiple alignment. In addition, a second "shuffled alignment" is produced by shuffling the alignments codon-wise (thus, always in multiples of three). Both alignments (the shuffled and unshuffled) are analyzed in the normal RNAz prediction pipeline as described above.

### Using GO-termfinder

All common Gene Ontology (GO) terms shared by CDS were detected using the GO-TermFinder perl modules [64]. These provide an object-oriented set of libraries for dealing with data produced by the Gene Ontology project. From this analysis all significant common GO-terms with a P-value smaller than 0.05 are reported. The P-values of a set of GO annotated genes is determined for a set of genes against the background of all genes in the genome sharing the same GO annotation. The P-value is calculated using the hypergeometric distribution as the probability of *x *or more out of *n *genes having a given annotation, given that *X *of *N *(equal the total number of genes) have that annotation in the genome in general.

### Website

A web-site featuring an interface for interactive data exploration and a Predicted RNA Yeast Genome Browser is available [65].

## Authors' contributions

SS co-developed the project idea, designed and implemented the software, performed the research and data analysis and wrote the manuscript. WH assisted in statistical analysis and participated in discussions. CS performed the reanalysis of the strand specificity of predicted RNAs. PFS developed the project idea, participated in discussions and revised the manuscript. KN developed the project idea, revised the manuscript, participated in discussions and supervised the overall project.

## Supplementary Material

Additional file 1 **Predicted RNA structures in fasta file format**. The element_id is decomposed as follows: StartCoord_Length_Chr_strand. First part of the file contains RNA structure elements found on the *P*_*SVM*_^0.5 ^and the second part RNA structure elements found on the *P*_*SVM*_^0.9 ^cutoff level. Click here for file

Additional file 2 **Distribution of z-scores of predicted structured RNA for each annotation class as reported by RNAz**. (A) PSVM∗
 MathType@MTEF@5@5@+=feaafiart1ev1aaatCvAUfKttLearuWrP9MDH5MBPbIqV92AaeXatLxBI9gBaebbnrfifHhDYfgasaacH8akY=wiFfYdH8Gipec8Eeeu0xXdbba9frFj0=OqFfea0dXdd9vqai=hGuQ8kuc9pgc9s8qqaq=dirpe0xb9q8qiLsFr0=vr0=vr0dc8meaabaqaciaacaGaaeqabaqabeGadaaakeaacqWGqbaudaqhaaWcbaGaem4uamLaemOvayLaemyta0eabaGaey4fIOcaaaaa@3278@ ≥ 0.5 (B) PSVM∗
 MathType@MTEF@5@5@+=feaafiart1ev1aaatCvAUfKttLearuWrP9MDH5MBPbIqV92AaeXatLxBI9gBaebbnrfifHhDYfgasaacH8akY=wiFfYdH8Gipec8Eeeu0xXdbba9frFj0=OqFfea0dXdd9vqai=hGuQ8kuc9pgc9s8qqaq=dirpe0xb9q8qiLsFr0=vr0=vr0dc8meaabaqaciaacaGaaeqabaqabeGadaaakeaacqWGqbaudaqhaaWcbaGaem4uamLaemOvayLaemyta0eabaGaey4fIOcaaaaa@3278@ ≥ 0.9 Click here for file

Additional file 3 **Known RNA genes in the yeast genome, covered by predicted RNA structures**. The annotation was taken from the *Saccharomyces *Genome Database. Structured elements with reported PSVM∗
 MathType@MTEF@5@5@+=feaafiart1ev1aaatCvAUfKttLearuWrP9MDH5MBPbIqV92AaeXatLxBI9gBaebbnrfifHhDYfgasaacH8akY=wiFfYdH8Gipec8Eeeu0xXdbba9frFj0=OqFfea0dXdd9vqai=hGuQ8kuc9pgc9s8qqaq=dirpe0xb9q8qiLsFr0=vr0=vr0dc8meaabaqaciaacaGaaeqabaqabeGadaaakeaacqWGqbaudaqhaaWcbaGaem4uamLaemOvayLaemyta0eabaGaey4fIOcaaaaa@3278@ values larger than 0.5 and 0.9, respectively, are shown. *S*_*E *_denotes the sensitivity to detect functional classes of known RNAs, ali refers to the number of elements in the input alignments, mpi is the mean percent identity of the windows. Click here for file

Additional file 4 **Predicted RNA structures annotation file**. The element_id is decomposed as follows: StartCoord_Length_Chr_strand. The first part of the file contains RNA structure elements found on the PSVM∗
 MathType@MTEF@5@5@+=feaafiart1ev1aaatCvAUfKttLearuWrP9MDH5MBPbIqV92AaeXatLxBI9gBaebbnrfifHhDYfgasaacH8akY=wiFfYdH8Gipec8Eeeu0xXdbba9frFj0=OqFfea0dXdd9vqai=hGuQ8kuc9pgc9s8qqaq=dirpe0xb9q8qiLsFr0=vr0=vr0dc8meaabaqaciaacaGaaeqabaqabeGadaaakeaacqWGqbaudaqhaaWcbaGaem4uamLaemOvayLaemyta0eabaGaey4fIOcaaaaa@3278@ ≥ 0.5 and the second part RNA structure elements found on the PSVM∗
 MathType@MTEF@5@5@+=feaafiart1ev1aaatCvAUfKttLearuWrP9MDH5MBPbIqV92AaeXatLxBI9gBaebbnrfifHhDYfgasaacH8akY=wiFfYdH8Gipec8Eeeu0xXdbba9frFj0=OqFfea0dXdd9vqai=hGuQ8kuc9pgc9s8qqaq=dirpe0xb9q8qiLsFr0=vr0=vr0dc8meaabaqaciaacaGaaeqabaqabeGadaaakeaacqWGqbaudaqhaaWcbaGaem4uamLaemOvayLaemyta0eabaGaey4fIOcaaaaa@3278@ ≥ 0.9 cut-off level. File isorganized as follows: element_id, strand of element on genome sequence, length of element, startcoord of overlap, endcoord of element, feature, feature name, strand of feature on genome sequence, length of feature, start coord of overlap, end coord of overlap. Click here for file

Additional file 5 **CDS with conserved RNA structure**. RNA structure found within CDS (PSVM∗
 MathType@MTEF@5@5@+=feaafiart1ev1aaatCvAUfKttLearuWrP9MDH5MBPbIqV92AaeXatLxBI9gBaebbnrfifHhDYfgasaacH8akY=wiFfYdH8Gipec8Eeeu0xXdbba9frFj0=OqFfea0dXdd9vqai=hGuQ8kuc9pgc9s8qqaq=dirpe0xb9q8qiLsFr0=vr0=vr0dc8meaabaqaciaacaGaaeqabaqabeGadaaakeaacqWGqbaudaqhaaWcbaGaem4uamLaemOvayLaemyta0eabaGaey4fIOcaaaaa@3278@ ≥ 0.5 cut-off level). File is formatted as: CDS identifier, start of predicted structure, end of predicted structure, mean percent identity. Click here for file

Additional file 6 **Predicted RNA structures in UTR regions**. The element_id is decomposed as follows: StartCoord_Length_Chr_strand. The file contains all RNA structure elements found on the PSVM∗
 MathType@MTEF@5@5@+=feaafiart1ev1aaatCvAUfKttLearuWrP9MDH5MBPbIqV92AaeXatLxBI9gBaebbnrfifHhDYfgasaacH8akY=wiFfYdH8Gipec8Eeeu0xXdbba9frFj0=OqFfea0dXdd9vqai=hGuQ8kuc9pgc9s8qqaq=dirpe0xb9q8qiLsFr0=vr0=vr0dc8meaabaqaciaacaGaaeqabaqabeGadaaakeaacqWGqbaudaqhaaWcbaGaem4uamLaemOvayLaemyta0eabaGaey4fIOcaaaaa@3278@ ≥ 0.5 cut-off level. File is formatted as element id, CDS with UTR, distance from CDS boundary, 5'/3' UTR. Click here for file

Additional file 7 **Predicted structured RNA overlapping with TF-binding sites**. Data is at the PSVM∗
 MathType@MTEF@5@5@+=feaafiart1ev1aaatCvAUfKttLearuWrP9MDH5MBPbIqV92AaeXatLxBI9gBaebbnrfifHhDYfgasaacH8akY=wiFfYdH8Gipec8Eeeu0xXdbba9frFj0=OqFfea0dXdd9vqai=hGuQ8kuc9pgc9s8qqaq=dirpe0xb9q8qiLsFr0=vr0=vr0dc8meaabaqaciaacaGaaeqabaqabeGadaaakeaacqWGqbaudaqhaaWcbaGaem4uamLaemOvayLaemyta0eabaGaey4fIOcaaaaa@3278@ ≥ 0.5 cut-off level. File is formatted as: element id, SGD identifier for TF-binding site. Click here for file

Additional file 8 **Structured RNAs providing evidence for snoRNAs**. The scores are given as reported by *snoSCAN *for C/D-box snoRNAs and *snoGPS *for H/ACA snoRNAs. The score-cutoff, as reported by the indiviual predictions tools, was defined by analysis of all known snoRNAs from yeast. We used the minimal reported score as cutoff (1 stem *snoGPS *= 14; 2 stem *snoGPS *= 21; *snoSCAN *= 14). RNA elements that were positively predicted by both, the 1 and 2 stem scanner mode of *snoGPS *are given in bold type. Click here for file

Additional file 9 **Predicted RNA structures in intergenic regions**. The element_id is decomposed as follows: StartCoord_Length_Chr_strand. The file contains all RNA structure elements found on the PSVM∗
 MathType@MTEF@5@5@+=feaafiart1ev1aaatCvAUfKttLearuWrP9MDH5MBPbIqV92AaeXatLxBI9gBaebbnrfifHhDYfgasaacH8akY=wiFfYdH8Gipec8Eeeu0xXdbba9frFj0=OqFfea0dXdd9vqai=hGuQ8kuc9pgc9s8qqaq=dirpe0xb9q8qiLsFr0=vr0=vr0dc8meaabaqaciaacaGaaeqabaqabeGadaaakeaacqWGqbaudaqhaaWcbaGaem4uamLaemOvayLaemyta0eabaGaey4fIOcaaaaa@3278@ ≥ 0.5 cut-off level. File is formatted as intergenic RNA elements overlapping with data from David et al, Davis et al, Samanta et al and with SAGE/EST data. Click here for file

Additional file 10 **Analysis of potential duplexes formed by predicted intergenic ncRNA transcripts**. First, we filtered potential duplexes by fast searches for overlap regions with wublast (Gish, W., personal communication) with parameters that also allow for G-U basepairs, as described in Steigele et al [3]. Second, the thermodynamically preferred duplex between two predicted RNA molecules was calculated by RNAcofold. In most cases, only very large overlaps between predicted RNA molecules were found. Click here for file
