# Supplementary figures and images for: Comparative analysis of structured RNAs in S. cerevisiae indicates a multitude of different functions
Source: BMC Biol. 2007 Jun 18;5:25. doi: 10.1186/1741-7007-5-25 (PMC1914338; doi:10.1186/1741-7007-5-25)

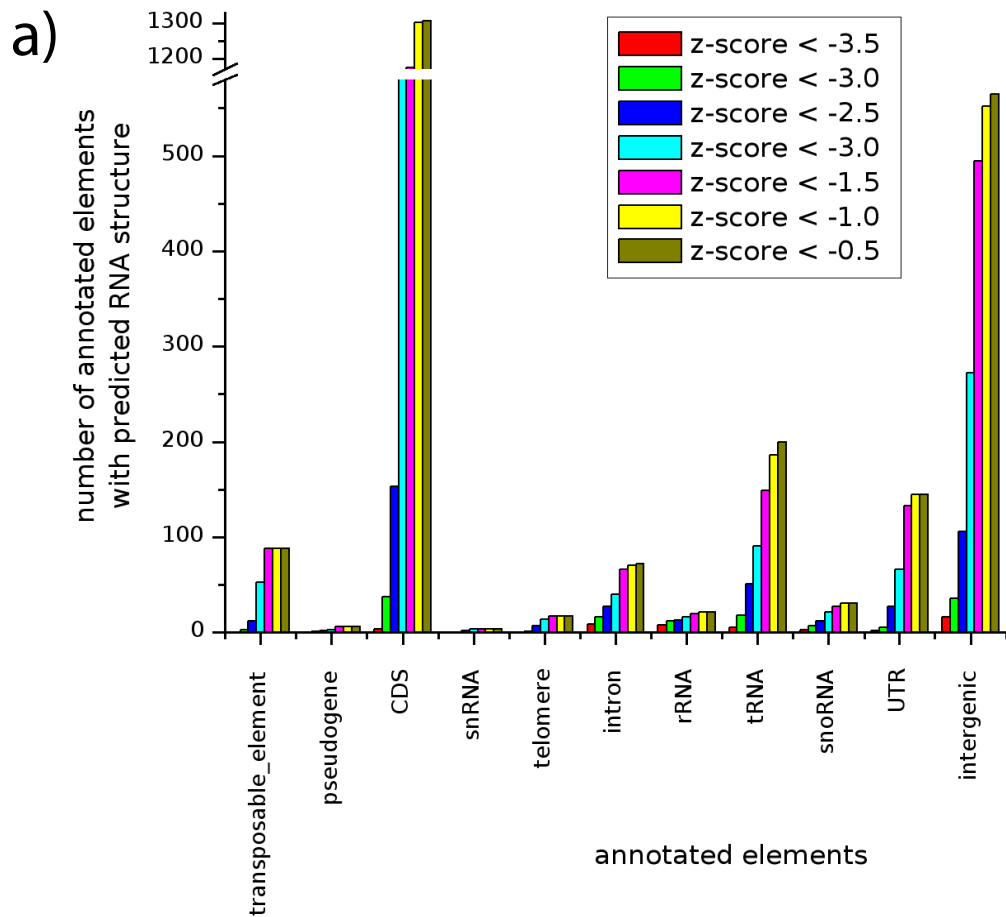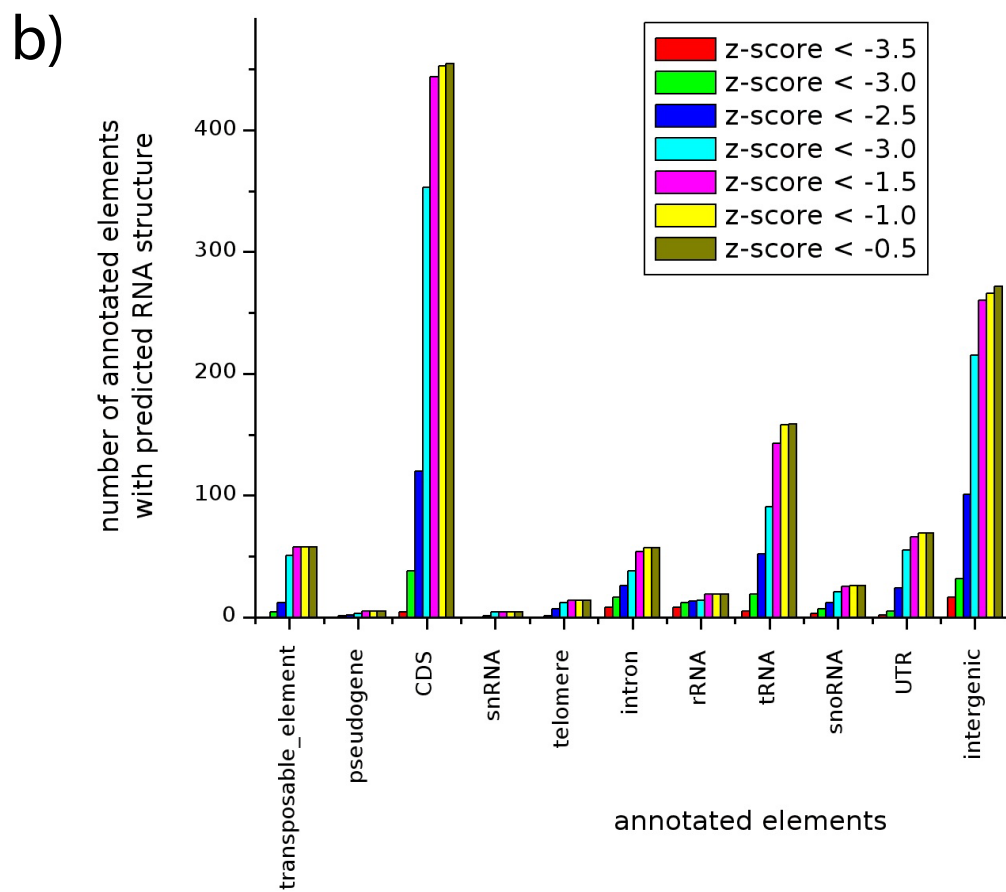

Supplement: Additional file 2 — Distribution of z-scores of predicted structured RNA for each annotation class as reported by RNAz. (A) PSVM∗ MathType@MTEF@5@5@+=feaafiart1ev1aaatCvAUfKttLearuWrP9MDH5MBPbIqV92AaeXatLxBI9gBaebbnrfifHhDYfgasaacH8akY=wiFfYdH8Gipec8Eeeu0xXdbba9frFj0=OqFfea0dXdd9vqai=hGuQ8kuc9pgc9s8qqaq=dirpe0xb9q8qiLsFr0=vr0=vr0dc8meaabaqaciaacaGaaeqabaqabeGadaaakeaacqWGqbaudaqhaaWcbaGaem4uamLaemOvayLaemyta0eabaGaey4fIOcaaaaa@3278@ ≥ 0.5 (B) PSVM∗ MathType@MTEF@5@5@+=feaafiart1ev1aaatCvAUfKttLearuWrP9MDH5MBPbIqV92AaeXatLxBI9gBaebbnrfifHhDYfgasaacH8akY=wiFfYdH8Gipec8Eeeu0xXdbba9frFj0=OqFfea0dXdd9vqai=hGuQ8kuc9pgc9s8qqaq=dirpe0xb9q8qiLsFr0=vr0=vr0dc8meaabaqaciaacaGaaeqabaqabeGadaaakeaacqWGqbaudaqhaaWcbaGaem4uamLaemOvayLaemyta0eabaGaey4fIOcaaaaa@3278@ ≥ 0.9 [file 1741-7007-5-25-S2.pdf]
