# Supplementary material for: Comparative analysis of structured RNAs in S. cerevisiae indicates a multitude of different functions
Source: BMC Biol. 2007 Jun 18;5:25. doi: 10.1186/1741-7007-5-25 (PMC1914338; doi:10.1186/1741-7007-5-25)
Supplement: Additional file 3 — Known RNA genes in the yeast genome, covered by predicted RNA structures. The annotation was taken from the Saccharomyces Genome Database. Structured elements with reported PSVM∗ MathType@MTEF@5@5@+=feaafiart1ev1aaatCvAUfKttLearuWrP9MDH5MBPbIqV92AaeXatLxBI9gBaebbnrfifHhDYfgasaacH8akY=wiFfYdH8Gipec8Eeeu0xXdbba9frFj0=OqFfea0dXdd9vqai=hGuQ8kuc9pgc9s8qqaq=dirpe0xb9q8qiLsFr0=vr0=vr0dc8meaabaqaciaacaGaaeqabaqabeGadaaakeaacqWGqbaudaqhaaWcbaGaem4uamLaemOvayLaemyta0eabaGaey4fIOcaaaaa@3278@ values larger than 0.5 and 0.9, respectively, are shown. SE denotes the sensitivity to detect functional classes of known RNAs, ali refers to the number of elements in the input alignments, mpi is the mean percent identity of the windows. [file 1741-7007-5-25-S3.pdf]

Additional file 3 — Known RNA genes in the yeast genome, covered by predicted RNA structures

Known RNA genes in the yeast genome, covered by predicted RNA structures. The annotation was taken from the `Saccharomyces Genome Database`. Structured elements with reported $P^{\star}_{\mathrm{SVM}}$-values larger than 0.5 and 0.9, resp., are shown. S_E denotes the sensitivity to detect functional classes of known RNAs, **ali** refers to the number of elements in the input alignments, mpi is the mean percent identity of the windows.

**Feature class**	$P^{\star}_{\mathrm{SVM}^{0.5}}$	$P^{\star}_{\mathrm{SVM}^{0.9}}$	**ali**	S_E	**SGD**	**mpi**
snoRNA	32	26	68	0.47	68	74%
tRNAs	200	159	274	0.72	299	85%
rRNA	21	19	27	0.78	27	94%
snRNA	4	4	6	0.66	6	78%
CDS	1309	455	6651	0.20	7050	79%
pseudogene	6	5	22	0.32	22	81%
introns	72	57	332	0.22	365	82%
transposons	50	50	50	1.00	50	78%
telomere region	17	14	22	0.77	32	84%
