# Supplementary material for: Comparative analysis of structured RNAs in S. cerevisiae indicates a multitude of different functions
Source: BMC Biol. 2007 Jun 18;5:25. doi: 10.1186/1741-7007-5-25 (PMC1914338; doi:10.1186/1741-7007-5-25)
Supplement: Additional file 8 — Structured RNAs providing evidence for snoRNAs. The scores are given as reported by snoSCAN for C/D-box snoRNAs and snoGPS for H/ACA snoRNAs. The score-cutoff, as reported by the indiviual predictions tools, was defined by analysis of all known snoRNAs from yeast. We used the minimal reported score as cutoff (1 stem snoGPS = 14; 2 stem snoGPS = 21; snoSCAN = 14). RNA elements that were positively predicted by both, the 1 and 2 stem scanner mode of snoGPS are given in bold type. [file 1741-7007-5-25-S8.pdf]

Additional file 8 — Structured RNAs providing evidence for snoRNAs
Structured RNAs providing evidence for snoRNAs. The scores are given as reported by *snoSCAN* for C/D-box snoRNAs and *snoGPS* for H/ACA snoRNAs. The score-cutoff, as reported by the indiviual predictions tools, was defined by analysis of all known snoRNAs from yeast. We used the minimal reported score as cutoff (1 stem *snoGPS* = 14;.2 stem *snoGPS* = 21; *snoSCAN* = 14). RNA elements, that were positively predicted by both, the 1 and 2 stem scanner mode of *snoGPS* are given in bold face.

**element id**	**score**	**start-end of predicted snoRNA**	**target modification site**	**additional snoRNA which targets the same site**
**H/ACA snoRNAs, 1 stem scanner**				
307255_121_2_-1	34.62	44-106	Cmpl: SSU.U766	Nd
296839_124_16_-1	22.96	14-76	Cmpl: SSU.U211	Nd
308828_138_3_-1	22.31	59-120	Cmpl: LSU.U2876	snR34b-E
426518_246_15_-1	22.15	42-117	Cmpl: LSU.U1003	snR5b-E
**1517880_200_4_1**	20.40	23-173	Cmpl: SSU.U1289	Nd
163190_121_3_-1	20.16	0-120	Cmpl: LSU.U2730	snR189b-E
296839_124_16_-1	20.01	14-76	Cmpl: LSU.U959	snR8a-E
931301_202_15_1	20.01	74-191	Cmpl: SSU.U120	Nd
296839_124_16_-1	19.68	4-76	Cmpl: LSU.U989	snR49-P
76850_126_12_1	19.67	36-120	Cmpl: LSU.U1051	Nd
220972_280_13_1	19.58	67-214	Cmpl: SSU.U1414	Nd
101133_361_13_-1	19.53	251-329	Cmpl: LSU.U2190	snR32-E
118914_157_10_1	19.53	57-141	Cmpl: LSU.U2257	Nd
76206_186_0_1	19.43	58-129	Cmpl: LSU.U2265	Nd
763744_120_14_1	19.10	26-97	Cmpl: LSU.U2128	snR11b-P
500842_161_13_1	18.80	20-110	Cmpl: SSU.U632	RNA161
203336_110_8_-1	18.79	14-91	Cmpl: LSU.U2822	snR34a-E
559168_200_5_-1	18.69	68-143	Cmpl: SSU.U1179	Nd
255367_120_6_-1	18.22	14-97	Cmpl: LSU.U1123	snR5a-E
559168_200_5_-1	17.87	19-108	Cmpl: LSU.U2348	Nd
**14909_343_4_-1**	17.81	31-176	Cmpl: LSU.U775	Nd
797761_210_16_1	17.75	39-170	Cmpl: LSU.U2940	snR37-E
9332_206_13_-1	17.72	100-204	Cmpl: LSU.U1041	snR33-E
**234368_239_4_-1**	17.69	3-93	Cmpl: SSU.U1000	snR31-E
987439_120_7_1	17.47	2-84	Cmpl: SSU.U302	Nd
**14909_343_4_-1**	17.36	70-130	Cmpl: SSU.U106	snR44a-P
264236_127_4_1	17.19	32-92	Cmpl: LSU.U2339	snR9-E
78330_202_3_1	17.11	11-103	Cmpl: LSU.U1109	Nd
1515496_120_4_-1	17.01	32-90	Cmpl: LSU.U2861	snR46-E
810933_96_7_-1	16.48	9-89	Cmpl: LSU.U985	snR8b-E
777355_157_13_-1	16.03	62-137	Cmpl: LSU.U965	snR43-P
76850_126_12_1	15.94	39-103	Cmpl: SSU.U466	snR189a-P
**1517880_200_4_1**	15.66	1-103	Cmpl: LSU.U2132	snR3b-P
**45140_291_2_1**	15.48	62-146	Cmpl: SSU.U1189	snR35-P
426518_246_15_-1	15.41	42-117	Cmpl: LSU.U2313	Nd
169854_127_4_-1	15.34	39-117	Cmpl: SSU.U1185	snR36-E
427179_360_15_-1	15.24	250-340	Cmpl: LSU.U1055	snR44b-P
164690_197_11_1	14.93	36-120	Cmpl: LSU.U2350	Nd
565854_280_5_1	14.80	171-252	Cmpl: SSU.U759	Nd

continued on next page

continued from previous page				
**element id**	**score**	**start-end of predicted snoRNA**	**target modification site**	**additional snoRNA which targets the same site**
**455692_200_10_-1**	14.29	60-186	Cmpl: LSU.U2263	snR3a-E
169854_127_4_-1	14.27	41-117	Cmpl: LSU.U2415	snR11a-E
**H/ACA snoRNAs, 2 stem scanner**				
29726_260_0_1	32.39	21-247	Cmpl: SSU.U120	Nd
33146_467_0_-1	29.53	227-453	Cmpl: LSU.U1051	Nd
29726_260_0_1	28.88	16-170	Cmpl: SSU.U211	Nd
**455692_200_10_-1**	27.89	3-175	Cmpl: SSU.U766	Nd
101133_361_13_-1	27.29	149-329	Cmpl: LSU.U2190	snR32-E
**14909_343_4_-1**	26.26	70-235	Cmpl: SSU.U106	snR44a-P
**1517880_200_4_1**	26.24	1-186	Cmpl: LSU.U2132	snR3b-P
88007_192_2_-1	23.40	12-176	Cmpl: LSU.U2861	snR46-E
929022_299_16_1	23.35	65-296	Cmpl: LSU.U1041	snR33-E
156266_161_16_-1	23.16	1-136	Cmpl: LSU.U959	snR8a-E
268349_431_6_1	23.09	101-363	Cmpl: LSU.U1055	snR44b-P
**45140_291_2_1**	22.88	52-281	Cmpl: LSU.U2940	snR37-E
**234368_239_4_-1**	21.77	3-236	Cmpl: SSU.U1000	snR31-E
2096_297_0_-1	21.46	74-256	Cmpl: LSU.U989	snR49-P
450723_361_12_-1	21.39	106-318	Cmpl: SSU.U759	Nd
**14909_343_4_-1**	21.35	31-295	Cmpl: LSU.U775	Nd
450723_361_12_-1	21.23	106-359	Cmpl: LSU.U2730	snR189b-E
**C/D-box snoRNAs**				
65374_120_14_1	22.89	9-106	Cmpl: ySc-25S-Gm865	snR50
482519_122_13_-1	17.11	122-45	Cmpl: ySc-25S-Um1307	Nd
482519_122_13_-1	16.26	122-45	Cmpl: ySc-25S-Um1307	Nd
168768_121_3_-1	15.06	43-119	Cmpl: ySc-25S-Um1886	snR62
290651_158_3_1	14.75	158-62	Cmpl: ySc-25S-Um2918	snR52
290651_158_3_1	14.71	158-62	Cmpl: ySc-25S-Am782	Nd
482519_122_13_-1	14.44	122-45	Cmpl: ySc-25S-Um1307	Nd
