# Supplementary material for: Comparative analysis of structured RNAs in S. cerevisiae indicates a multitude of different functions
Source: BMC Biol. 2007 Jun 18;5:25. doi: 10.1186/1741-7007-5-25 (PMC1914338; doi:10.1186/1741-7007-5-25)

1 68377_120_1_-1 . . . 68417_160_1_1

68377_120_1_-1

CATTACTGATTTGGGAAATTTCCCAAATTGGAAATATCACCTCGTCGACGTGCTGCGGTG
CATGGCTGTTTTACCCGTTTAGGAAAAAAACTCGGCGGGTTTTCTTGACGGGCAAATGTCG

68417_160_1_1

AAACGGGTAAAACAGCCATGCACCGCAGCACGTCGACGAGGTGATATTTCCAATTTGGGA
AATTTCCCAAATCAGTAATGTAGCCTCTACGGGTGTCTCTGTCAGCCCCGTGGTCGCCAG
CACAGAATGTATCGTACCCCTGAAGGTAGTTTTTTACCGC

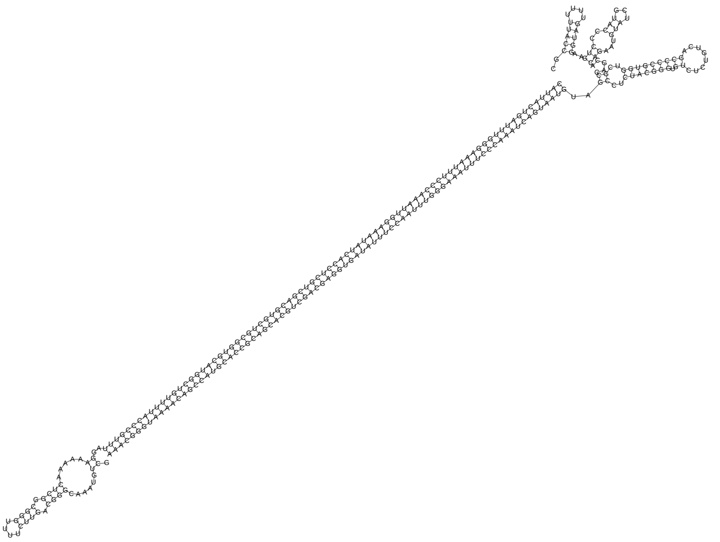

2 176465_196_1_-1 . . . 176465_196_1_-1

176465_196_1_-1

CAAACTAGAGTCCCTGCTTTACGAATGAAACCTGTACAGGTATGAGAGGTTACGAAAAAA
AATGATAAATGAAAAAAAAAAAAGCTTAATAATATGAAATTAATAGTATGAATAGTTAGGC
GCATGTATATAAATATAAATTTTTGTATATATGTATTATTAATATTATAAATTTCTTATT
ATTAAACCATCCTTTA

176465_196_1_-1

CAAACTAGAGTCCCTGCTTTACGAATGAAACCTGTACAGGTATGAGAGGTTACGAAAAAA
AATGATAAATGAAAAAAAAAAAAGCTTAATAATATGAAATTAATAGTATGAATAGTTAGGC
GCATGTATATAAATATAAATTTTTGTATATATGTATTATTAATATTATAAATTTCTTATT
ATTAAACCATCCTTTA

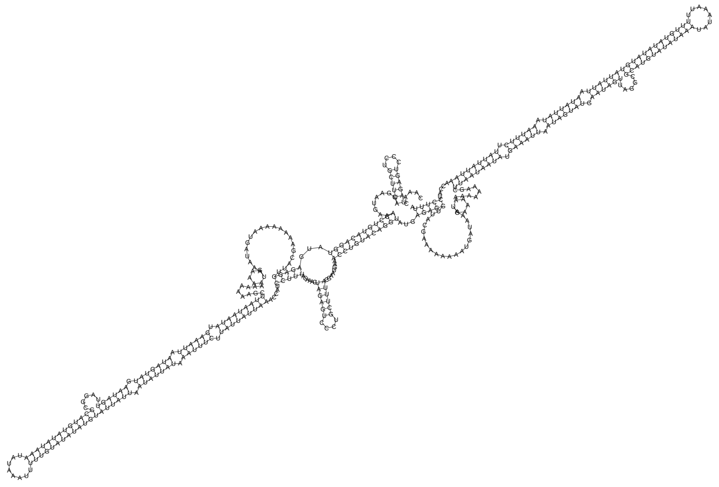

3 177376_120_1_-1 . . . 177336_121_1_1

177376_120_1_-1

TTAATTTTTCATTGTTTCTAATTTGGGAAAAGTCCGATTTCCCTCCTACTAACCGTTTAG
TGCCAAGGGTGAGATGGCCCTTGAACGAGGGCCCAAAATAAATTTGGCTTTGGTTCTATT

177336_121_1_1

AAAAGTGCCTATAGGGCTGCAGCTGCAGTTTTGGCCAAGAAATAGAACCAAAGCCAAATT
TATTTTGGGCCCTCGTTCAAGGGCCATCTCACCCTTGGCACTAAACGGTTAGTAGGAGGG
A

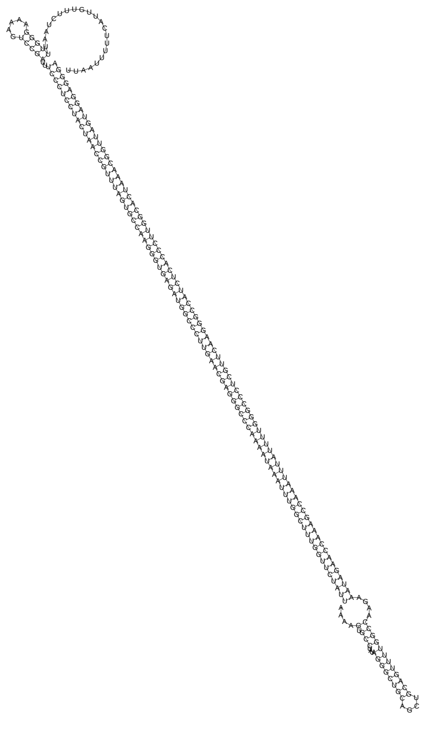

4 144137_119_11_-1 . . . 144176_119_11_1

144137_119_11_-1

CTTGATTTGGCGCATGTTGTAATGAATGGAGTAGGGATAATCCTGGAGATGCATGATTTA
TCCCTTCTATTTCAGTCGACGGCGAAATATGCCAAATTTAGAAAGCCCTCGGCCTTTGA

144176_119_11_1

GTCGACTGAAATAGAAGGGATAAATCATGCATCTCCAGGATTATCCCTACTCCATTCATT
ACAACATGCGCCAAATCAAGCCTATATAAGATTCTCGTCATTTAGCATGCTCTATTGAT

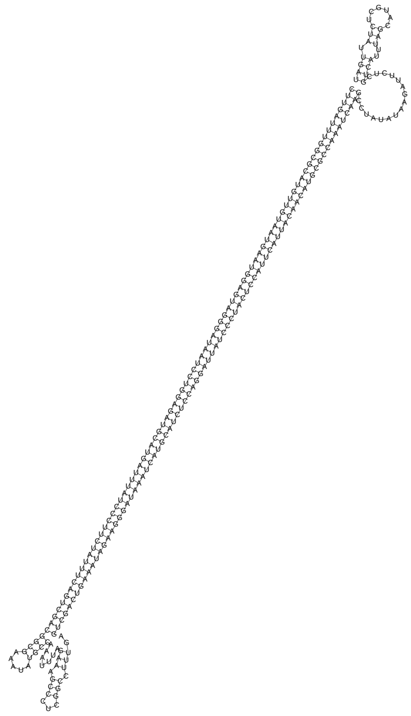

5 289857_120_12_-1 . . . 289817_120_12_1

289857_120_12_-1

TGCACAGTTAACTTTCTAGCAGGAGTATAATGCCATTTGCTCCCCATCTTGAGATGGGAA
GGGCTTAACTAATCTCGGTTCGGAGTGATCCGCCCCGATACTGCCTTCTGCCTTAATATC

289817_120_12_1

GGAGATATCTGCGCCGTTCAGGGGTCCATGTGCCTTGGACGATATTAAGGCAGAAGGCAG
TATCGGGGCGGATCACTCCGAACCGAGATTAGTTAAGCCCTTCCCATCTCAAGATGGGGA

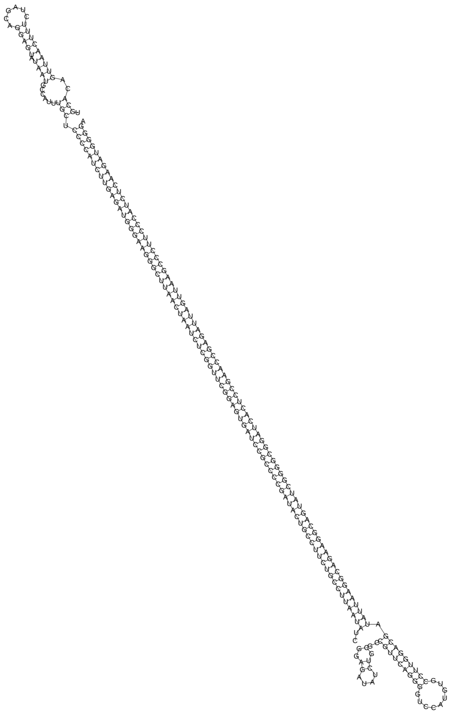

6 534534_117_12_-1 . . . 534573_158_12_1

534534_117_12_-1

GGACAAAATTAACCCTGCTTACACCAGGGTTTATCTAGAAATAAAATAGGTCATTGTTTT
GAGATGACTTGGAAAAACTTAACCCTGATGTGACTGTAACATAACATCTAAGAAAAA

534573_158_12_1

GTTTTTCCAAGTCATCTCAAAACAATGACCTATTTTATTTCTAGATAAACCCTGGTGTAA
GCAGGGTTAATTTTGTCCCAAACGGGCAAAATATAAATACCCCTTTCGGGAAATAAACTA
AAAAGAGTTCTAATTAGCCAATTGGCAAGAAAGCTCGA

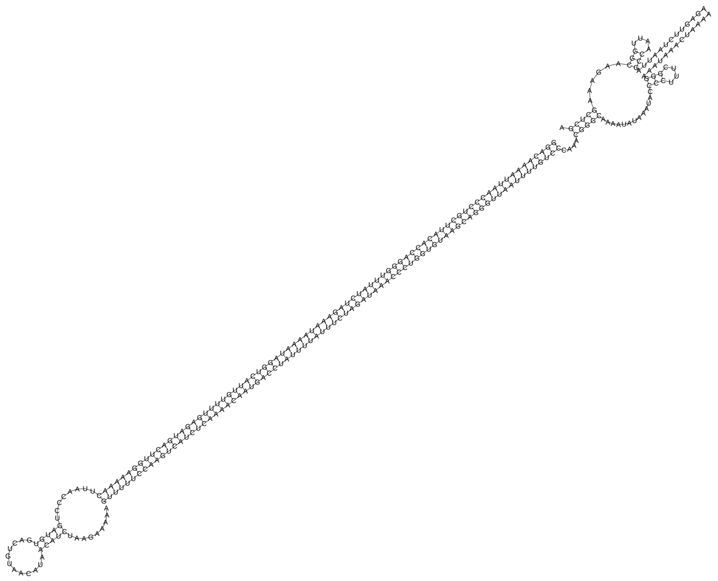

7 602654_123_12_-1 . . . 602614_121_12_1

602654_123_12_-1

GTAATCATTCGTTCTTCGACTTTTATAGCCTTTTTTTTAACTTAACGAACTCGTACTATC
TAATAGTTTATTTCCCCTTGTTTTTTTTTGTTACGATTGGGGAAAAGCAAGGGTTCCCTG
CGT

602614_121_12_1

ATCGATTTCGGTGACTAGTTAGTAGATGATGTAATCGTAGACGCAGGGAACCCTTGCTTT
TCCCCAATCGTAACAAAAAAAAACAAGGGGAAATAAACTATTAGATAGTACGAGTTCGTT
A

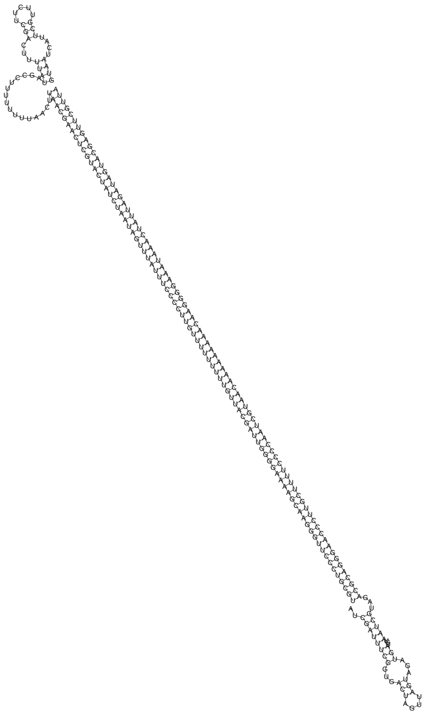

8 **789942_120_12_-1 ... 789982_162_12_1**

789942_120_12_-1

CTCAGCCTCGAGAAACATTCCTCGCGGCATTTCCGTGAGATCTCTCAATTAATTTCTTTT
TTTAGATTTTTTTCCGTTTTCCGTTTGAATAATTTCGCGTTTTCGTAGAGCGCGACGGCC

789982_162_12_1

AAAACGGAAAAAAATCTAAAAAAAGAAATTAATTGAGAGATCTCACGGAAATGCCGCGAG
GAATGTTTCTCGAGGCTGAGCGGCGTGGTCTGTGCAAAAAAATGGCAATTTTTTTGTAGG
AGTTTGCATTGGGCCATTCAGAAGGAGCACCGTTAGATGGGA

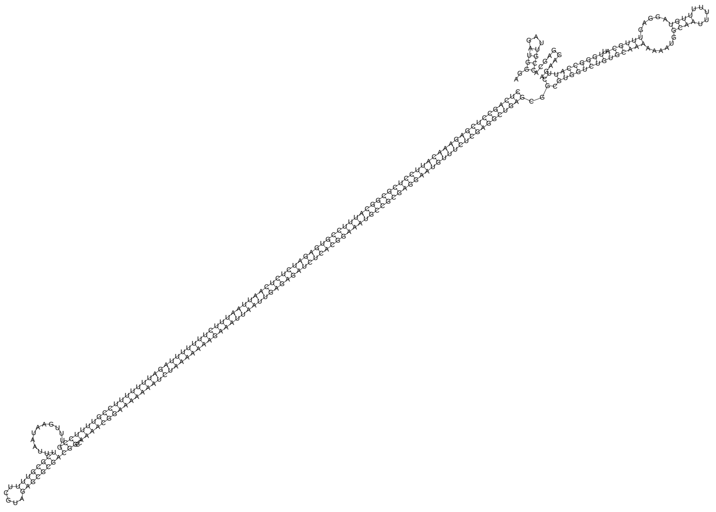

9 481901_121_13_-1 . . . 481942_123_13_1

481901_121_13_-1

AAGACTCCTTATATATGACAGTTTGCTGTATTTGTACAGTTGGACCGTCTTCATTACAAC
AATATTTATTGAACAGTAGCTTGTAATAGGCCGGCATTTTTTTGGTTAATAACAATGCCA
G

481942_123_13_1

GCTACTGTTCAATAAATATTGTTGTAATGAAGACGGTCCAACTGTACAAATACAGCAAAC
TGTCATATATAAGGAGTCTTATGTGACAGCACTTGCGTTATTGTCAGCCGGAGTATGTCT
TTG

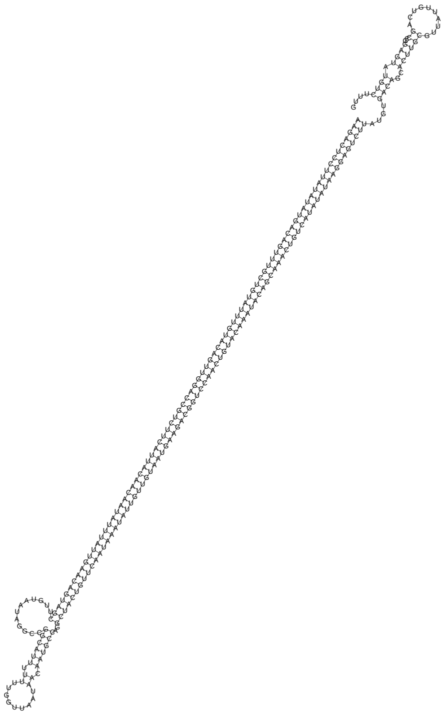

10 861691_124_13_-1 . . . 861651_124_13_1

861691_124_13_-1

TTATTTCGGAGCTTTTTTGCTGGCATTCTTATCTCGTGCTTTTATTAGTGGGCCCCTTCT
TTGAGACCCCGCGGGTGATATGGCCCCTGTCTCAATGATGGATAAAGTTGTTGGAAGCAA
TTAC

861651_124_13_1

CAGTACACTTCGGTAGCAACCTTCGTTTGTGATTGTCTTGGTAATTGCTTCCAACAACTT
TATCCATCATTGAGACAGGGGCCATATCACCCGCGGGGTCTCAAAGAAGGGGCCCACTAA
TAAA

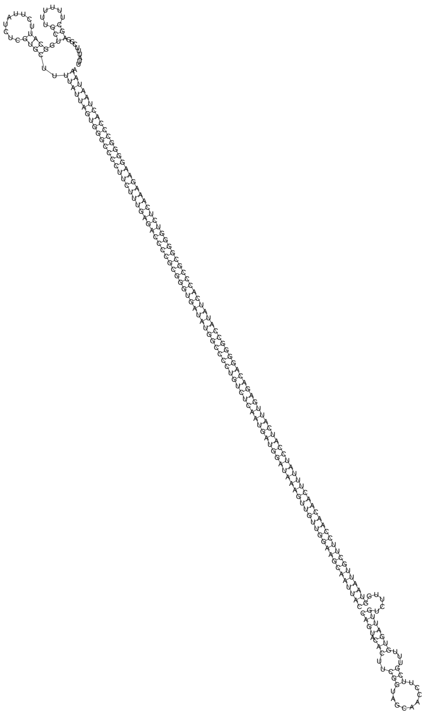

11 87516_120_14_-1 . . . 87556_120_14_1

87516_120_14_-1

TTCGAGTCGCTTTTGCGTCGCATGCCTCCGCGAAAAATTTCGCGCCGGACGCGATTTTTA
ACACGAAATTCTTGTTCGTGCCGCTGTTGCCCTTTTGGGAAATATTTCCCGATCTGGCAT

87556_120_14_1

CACGAACAAGAATTTCGTGTTAAAAATCGCGTCCGGCGCGAAATTTTTCGCGGAGGCATG
CGACGCAAAAGCGACTCGAAATGTCGGGAGCCAAATGAGGCTACAAGGCTGTGGGCAGAT

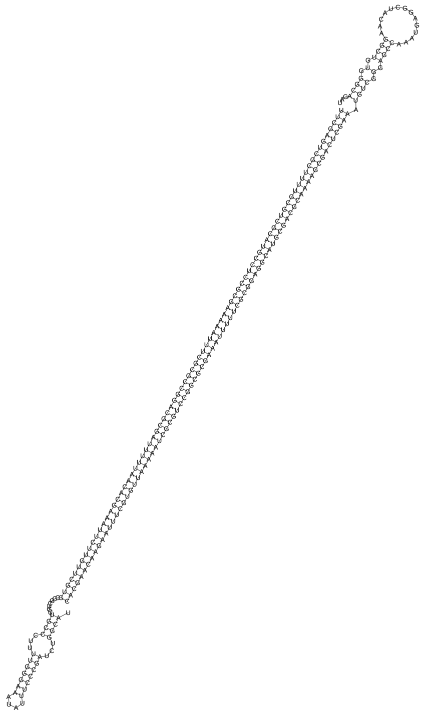

12 405735_125_14_-1 . . . 405776_124_14_1

405735_125_14_-1

GGAAGCTATGATATGGGGATTTCCATTGAGCCGATAGCAATGTAGGGTAATACTGTTGCG
TATATAGTGATAGTTATTGAATTTTATTACCCTGCGGGAATATTGAGACATCACTAAGCA
CGAAT

405776_124_14_1

AAATTCAATAACTATCACTATATACGCAACAGTATTACCCTACATTGCTATCGGCTCAAT
GGAAATCCCCATATCATAGCTTCCATTGGGCCGATGAAGTTAGTCGACGGATAGAAGCGG
TTGT

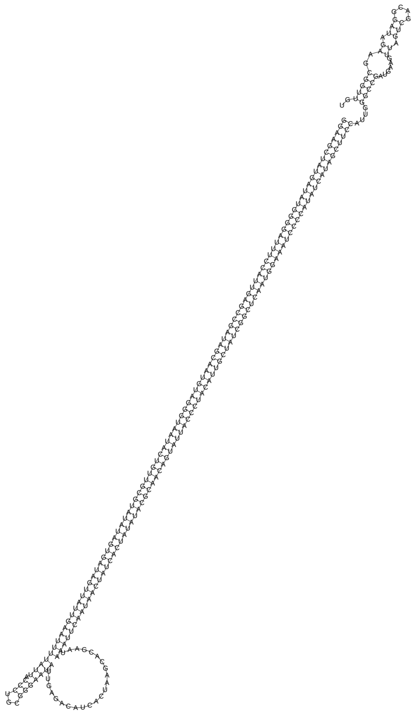

13 1004686_117_15_-1 . . . 1004646_121_15_1

1004686_117_15_-1

AGAAAATAAAAAAAAATTAAAACTCAAAATAAAATGTATTCCAATGTAAAGGAAAAAAATAA
AATAATAAGGAAAAAAGAAACTATTATTTTAAAGCCTTCAATAATGGAATGCTGAAA

1004646_121_15_1

TTGACTATTTGACTCAAATTACCAAAATCATAACTTTTCTTTTCAGCATTCCATTATTGA
AGGCTTTAAAATAATAGTTTCTTTTTTCCTTATTATTTTATTTTTTCCTTTACATTGGAA
T

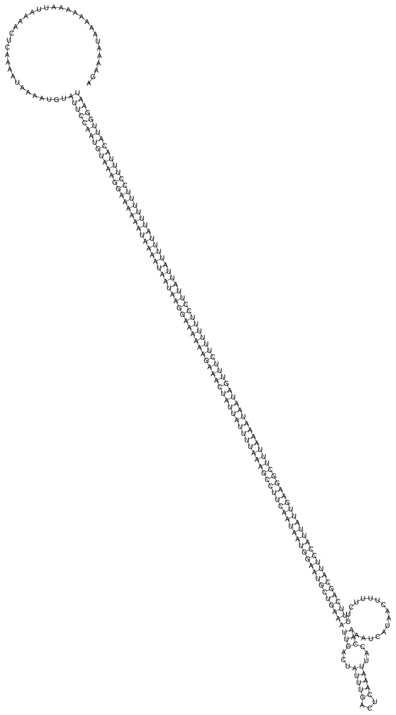

14 1057918_121_15_-1 . . . 1057877_121_15_1

1057918_121_15_-1

AATAGAATAGCGTGTAGTTGTTAAAATTTTATTTCACTGAGGCTTATATGCTTACTGAGA
AATTTTTTTTTTTTTACAAAAAACTTCTTTTTATGTTATACCAGCTGTGGATTACCACAAC
A

1057877_121_15_1

AATTAACTGAATTTTTTTTTCTGGATTGATGTTCTTTCCAATTGTTGTGGTAATCCACAGC
TGGTATAACATAAAAAGAAGTTTTTTGTAAAAAAAAAAAAATTTCTCAGTAAGCATATAAG
C

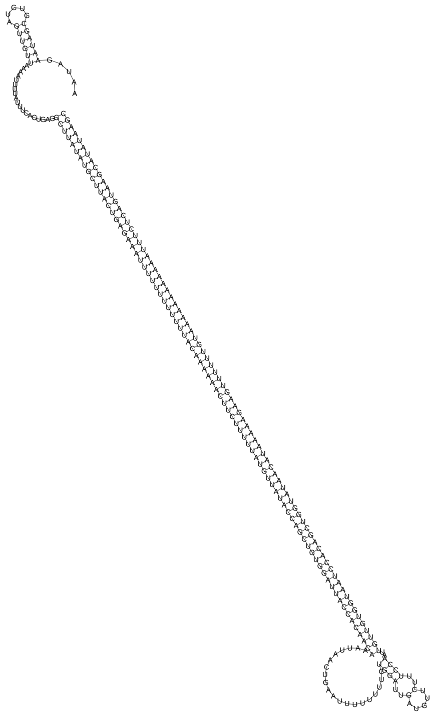

15 425979_122_16_-1 . . . 425897_158_16_1

425979_122_16_-1

TCTTTCTGTCAAACTAGCGCGCTTTTGGCAATCTCCATTTTCCTTCTTTTTTTTTCCGTT
TTTGTTATACTAAACAACTTCGGGGCGCGTTATAGATAGAACGACTTATACACATCTATT
TT

425897_158_16_1

ATGCTGGCGTCCTTAGCCTCACGCCTACTAAACAAGAGCATCTTACTCTCACCATCCATT
TATATACATATATAAGTACGTAAAAATAGATGTGTATAAGTCGTTCTATCTATAACGCGC
CCCGAAGTTGTTTAGTATAACAAAAACGGAAAAAAAAA

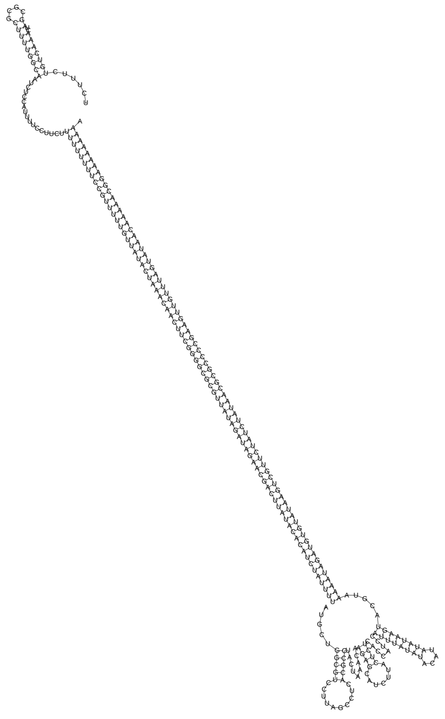

16 812791_123_16_-1 ... 812831_165_16_1

812791_123_16_-1

AGCTGGTTTGCCCGCGATAAGGCGGGCGAGTTATTTTGAAGTTTTCCATAAACTGGTTTT
CCATCTCGAGGTTTTTCCTCGCTTTCCACGCTATGACCCTTTTTAGTTAAGGTACCCGAT
GGC

812831_165_16_1

AGCGAGGAAAAACCTCGAGATGGAAAACCAGTTTATGGAAAACTTCAAAATAACTCGCCC
GCCTTATCGCGGGCAAACCAGCTACGAGAGATAAGGATTTGGCGCCGAGATAAGGTGGAG
ATGTTTCTCCGCCGCGCGGCACTTTTTGCCGGAACTGGAACTGGT

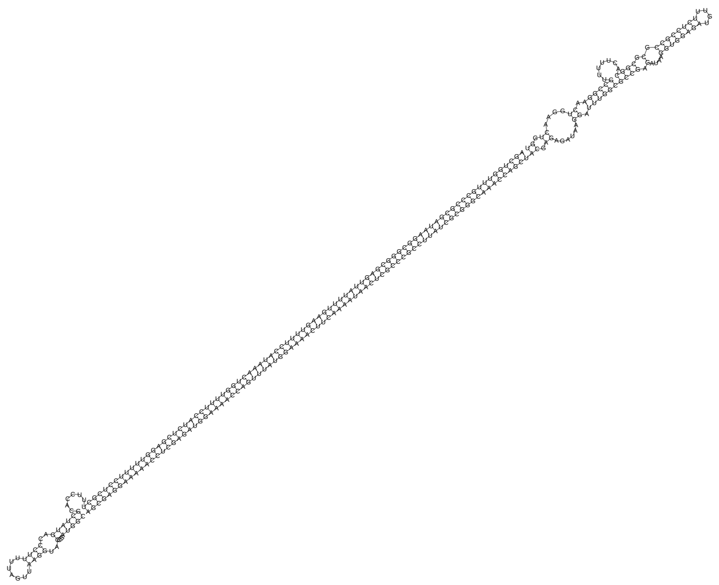

17 830482_202_16_-1 . . . 830605_137_16_1

830482_202_16_-1

TAGAAATAAGACTTCTTCATAATCGTTAATGAAGAAGTGCATATGATTTGTTATTTTTTT
TCTGGTATTTTTTTCATTTTTTTCGGGTTTTCCTTGGGTATAATTTTTTGTTTCTTCCGA
AAGACGGAAAGGGATGAGGGAAAACGGAGTTTTCTTATTTGCTTTCTTTTTTCCCTAATT
TATTCACGGTGTCCATTTATTA

830605_137_16_1

AAATGAAAAAAAATACCAGAAAAAAAAATAACAAATCATATGCACTTCTTCATTAACGATTA
TGAAGAAGTCTTATTTCTAACTAATATTATTATTATTATTATTATTATTATCATTCACCC
CGCTTTCTTTTATTATA

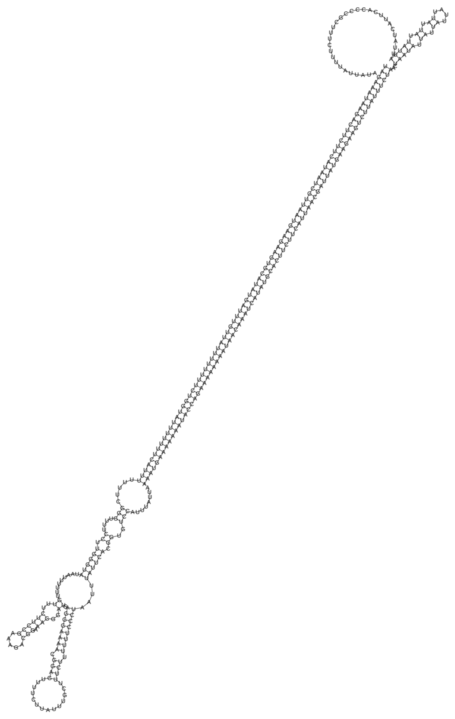

18 1370_244_4_-1 . . . 1269_305_4_1

1370_244_4_-1

AAGGGTATCCATTTCATTTCCTGTTTTCTATCTAGCCAATTCAATAATCGTAATAACGAA
TTTACAAAACTTTAATGCGGGTACATCACGGTGTGCCGTAACGTTGGTTTACATTTAAGG
TGCGACCAGCAATGTCACTGCTCGTACAGCGGTTACCGCTTGAATGAAATACATATTTTA
AGCGTAGATTTCCTGTGACAAAACTGTGTAAACATCTAGTAGAAAATAGACGTAGCTGTC
ACAA

1269_305_4_1

CTATTGACATGGTATCGAAAGGTTGTCCACATTGGGAAGTAACTTGGTTCTATGAATCTT
CATGTCAGATACGTAGGACAGACTCTTTCCTGTGTAAATATTTGTGACAGCTACGTCTAT
TTTCTACTAGATGTTTACACAGTTTTGTCACAGGAAATCTACGCTTAAAATATGTATTTC
ATTCAAGCGGTAACCGCTGTACGAGCAGTGACATTGCTGGTCGCACCTTAAATGTAAACC
AACGTTACGGCACACCGTGATGTACCCGCATTAAAGTTTTGTAAATTCGTTATTACGATT
ATTGA

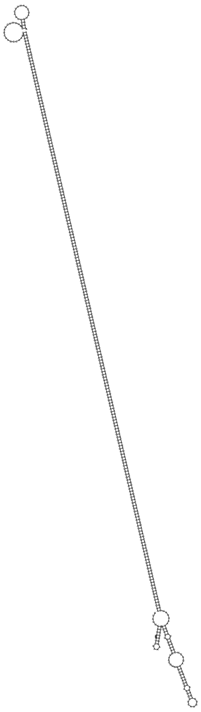

19 60648_126_4_-1 . . . 60609_124_4_1

60648_126_4_-1

GTGACATGGTATAATCCATATGTATCGTGCTGAAATTTTTTCTTGGAGATTTTCATTTAT
TCTCCACCATTCTTTCTCGCCTCCCCTCCTCCTTTTCCTTCTAATAATAACTTAGTTCTT
TTTCCT

60609_124_4_1

TTATTGACGTTTTTTTTTTCTTAGTCTCTTTAATGAAGAAGGAAAAAGAACTAAGTTATT
ATTAGAAGGAAAAGGAGGAGGGGAGGCGAGAAAGAATGGTGGAGAATAAATGAAAATCTC
CAAG

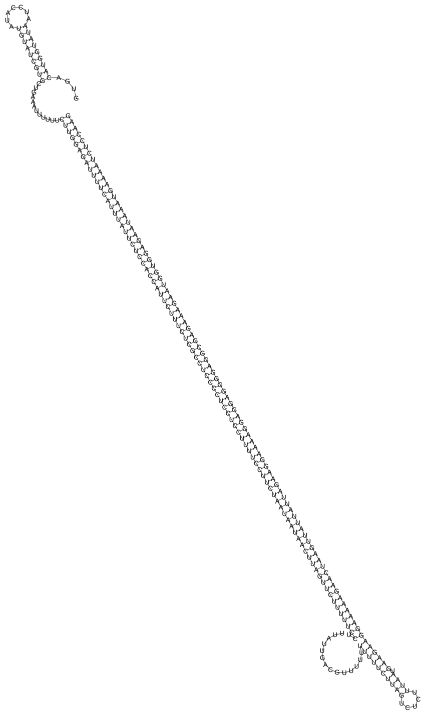

20 1330771_121_4_-1 . . . 1330725_124_4_1

1330771_121_4_-1

GATATTGTATATATTCAATTGCACCTCTTAACTGGTACTTTATTTCTTGTGAATGTTCGA
TATCTTACCGCTTATTCTCTCATTCCATATGAGAACTCTCGTATAGAAAGGGAGACCTTT
T

1330725_124_4_1

CCAAGCCGATCTATTTTGGATATCTTATTGTCATTTTTCAAGTGAAAAAAGGTCTCCCTT
TCTATACGAGAGTTCTCATATGGAATGAGAGAATAAGCGGTAAGATATCGAACATTCACA
AGAA

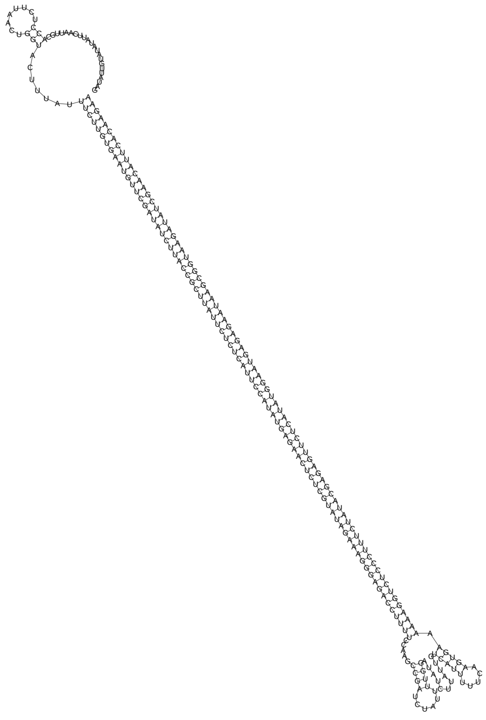

21 1516529_286_4_-1 . . . 1516735_200_4_1

1516529_286_4_-1

TTTAAGCACAAGATATAGACGATACCATACATGAATGTCGATCAGAGACGTATGGTATAG
CTTGTGTCACATTCTGCTATCGTATATGGTATACATGGCACATACTTTAAATTTTAAAGT
AATGTCACTTGATTAAGATCTTTCCTATACGTCAAGGTCTCGAGATAAAACTTTATGTCT
CAAATAATACATGCTAGGCATATAATGACCCATTTTCTGGAGTTGATGTACTCAAGACAT
TGGTTAAATATCAATCACGCATGATGATAGTGCGCCCTATTATAAC

1516735_200_4_1

ATAGCAGAATGTGACACAAGCTATACCATACGTCTCTGATCGACATTCATGTATGGTATC
GTCTATATCTTGTGCTTAAAGGTAGCGTATAGTAAGGTAATTACTATATGATCAATACTG
TGTGTGTAGAGTACAATAAAATATATGGGAAGAACATAAAAAGTTCGTATTGTGACATGT
TATTGAGGCTATGGTAAGCG

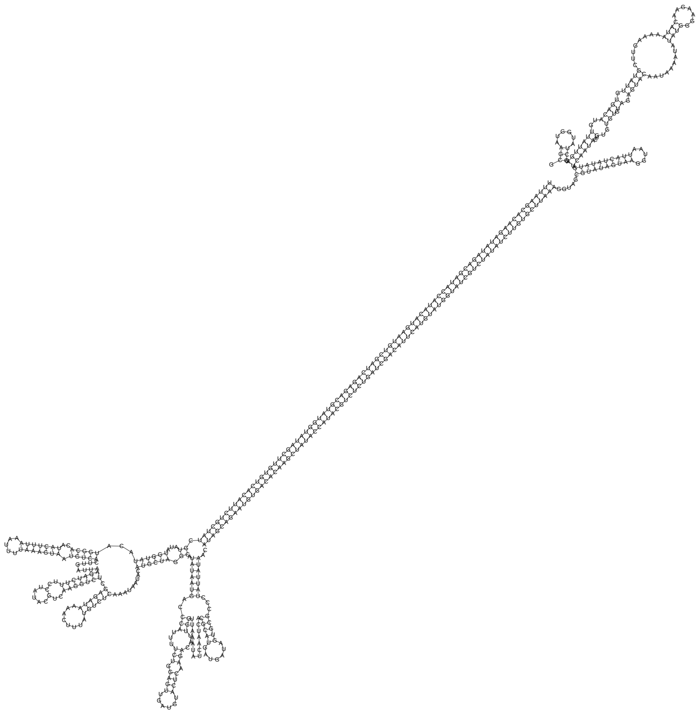

22 1518000_200_4_-1 . . . 1517880_200_4_1

1518000_200_4_-1

TTGCGTTCTTACGTCAAAGAACAATGATGCTACGGAGCGCAAGAAAGTGACGTGGCCAAC
TATCAGCGCGCTAGGATATGTATGCACTGCCGAAAGGCTGAAGCGTATCGTACATTAATT
CATAAAGAGAAAGAAATCTCAACCCTGAATTTAGGATAGCGAAGTGCAATAAGAAAAACT
TTACCATTGTTTTCAGGATG

1517880_200_4_1

GATATCCGCTCGAGATTAAGTGCGGCCCTCTCCACCCGATAAGGAACAAAGGGCATGCTT
CCTTTTTTAGCTTTACGCACTATTGTCAAATTGTTTATATAAACAGGCTGATATAGAATT
CATCCTGAAAACAATGGTAAAGTTTTTCTTATTGCACTTCGCTATCCTAAATTCAGGGTT
GAGATTTCTTTCTCTTTATG

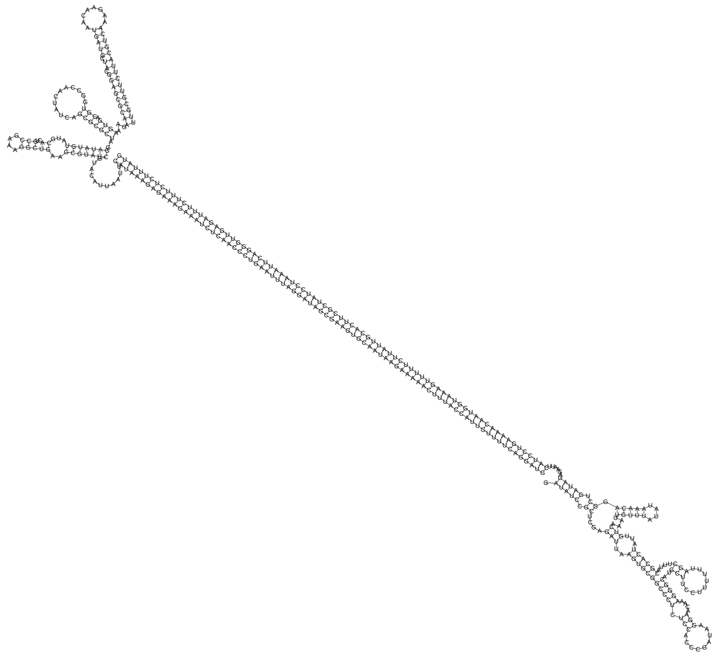

23 77751_161_5_-1 . . . 77832_120_5_1

77751_161_5_-1

TAATTTGGAAACTATCCACCTTGAAGATTCCCACTAAATCTTCGAAGGACGACGATTTTC
TTCGAGGTTGAAAAAAGTTTTTCAAGAATTTTCGCGCTTCTTCCTAATCGGGAATTTATC
TGTCATAATTATAATTCCCTATTTGGGAATTGGAACGAAAT

77832_120_5_1

AAACTTTTTTCAACCTCGAAGAAAATCGTCGTCCTTCGAAGATTTAGTGGGAATCTTCAA
GGTGGATAGTTTCCAAATTAAAACTGCAGAAGAGGCTTTTAAAACATGCCTGAGGTATAA

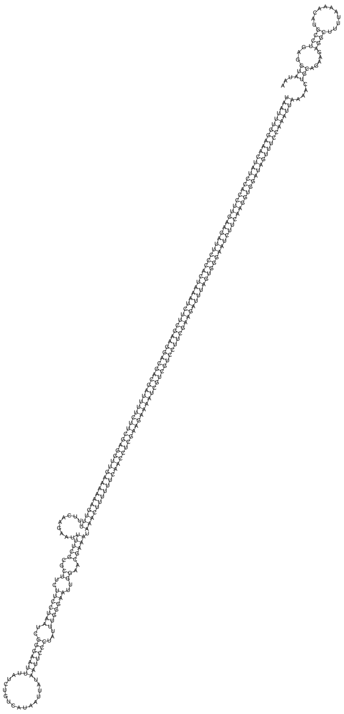

24 322046_121_5_-1 . . . 322006_121_5_1

322046_121_5_-1

GATTAAACAATGGCATTTCTATGGATTTGTTGTTCTCTTTATATATTTGAGGAAAATTCA
AAGATAATGGGGAGAAAGGTGGAGATGTATCTCCACCTATCGTAAATTTGAAAGTTCCGC
T

322006_121_5_1

TAAAAAGCGTTGCGTGATGTTTGGGTGCAATTGGCGGAAAAGCGGAACTTTCAAATTTAC
GATAGGTGGAGATACATCTCCACCTTTCTCCCCATTATCTTTGAATTTTCCTCAAATATA
T

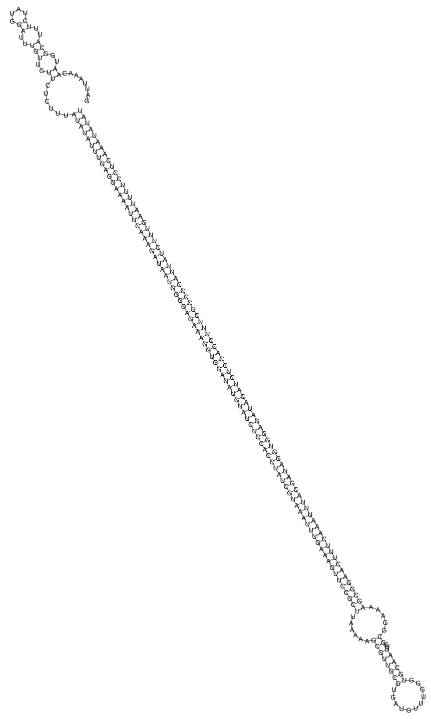

25 559168_200_5_-1 . . . 559126_122_5_1

559168_200_5_-1

CAAGAGACTATACATCCCACAATTGATTCGGTAGGTGCTTTTATACTGATTCATTTGAGG
TCTTGGCATTTGTTCAATTGAAGTACATTTAGTTGAAAAGTTGAGATTTATTTAGGATGA
GCTTGCCGCGGCATGGGGAAAGAAACACTTCATCCTGTATGGAGAAAAACCACTTTGCCG
CGGCACATCAAAACAGGGCT

559126_122_5_1

ATGCGATTGTCGTGTAATGGTATCGCGCATACTGAAGGTTTCAGCCCTGTTTTGATGTGC
CGCGGCAAAGTGGTTTTTCTCCATACAGGATGAAGTGTTTCTTTCCCCATGCCGCGGCAA
GC

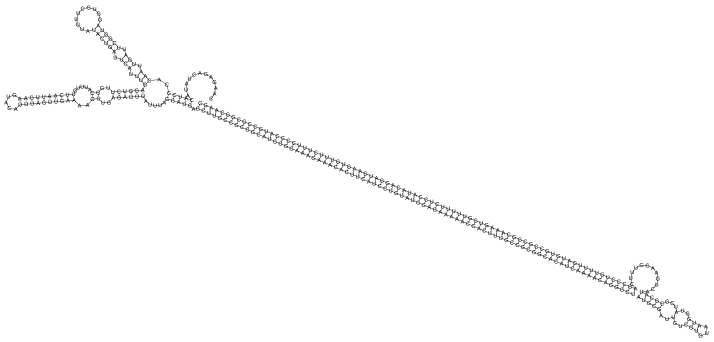

26 99910_119_6_-1 . . . 177336_121_1_1

99910_119_6_-1

GTTTTTTCTCCTAGCAACCGTTTAGTGCCAAGGGTTAGGCAATTGAACGAGGCCAAGACA
ATATTGGCTTTGCTTCTATTACTTGGCTAACATTGTGTCTGCAGGTCGAAAGGCACCTT

177336_121_1_1

AAAAGTGCCTATAGGGCTGCAGCTGCAGTTTTGGCCAAGAAATAGAACCAAAGCCAAATT
TATTTTGGGCCCTCGTTCAAGGGCCATCTCACCCTTGGCACTAAACGGTTAGTAGGAGGG
A

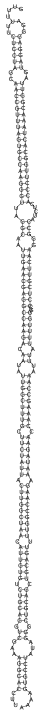

27 255367_120_6_-1 ... 255408_119_6_1

255367_120_6_-1

CGTTTGTTGGAAGATAGCGAAATACCTTACTGGAGCAACCAGGAAAAATACTCTGGTTGC
AAAAACCAACAAAAGAAAAAAATGGAAGACCTAAGAACTATGCATTTTTTTTTAAGGGGTT

255408_119_6_1

TTTTCTTTTGTTGGTTTTTGCAACCAGAGTATTTTTCCTGGTTGCTCCAGTAAGGTATTT
CGCTATCTTCCAACAAACGGGGCACACGTGCGGGAGTTTCAAAGGGGCAGAATAGTGGG

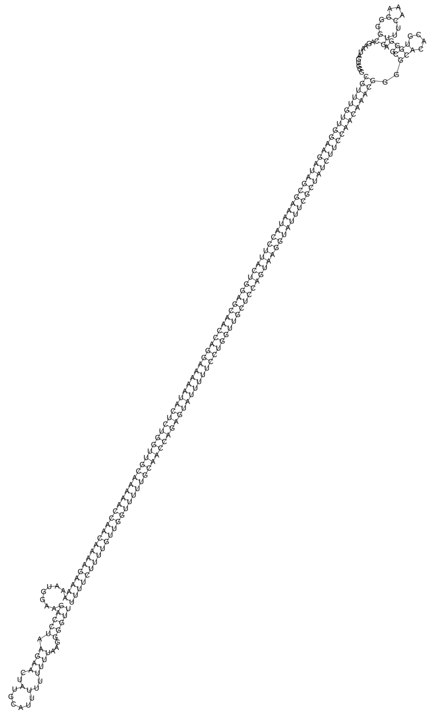

28 268591_148_6_-1 ... 268349_431_6_1

268591_148_6_-1

TTGATCTGAGAGAAGTTAGTGATATGAAATATCAATACATAGTATCTTATTTAGATTACG
CATACCTTTTTTTTTCTGCAATTGAGGCAGGATTTATTCCTACATAGTACTGCTTTTTAC
ATTGTATTGTTTTTTTTTGTTTTTCTTT

268349_431_6_1

GATTTCTTCTCGAGTCATATATACTATTACATGAGTTCGAGTATGGTGTTGTTTTCTTTA
GAATGTCTTATCAAGACCGACAATTCATACAGGTCGAGATATGTAAAGTCTACATGCCGA
GCTAGGATGAGAAAAAAAGTATACAAATTGCAGGCAAATAAACACACCAAAAGATACTAA
AAAAATCTTTTAAAGTTTTGGTTAGTTATTGGGGATCATCATGCATTTAACGACAACAGA
GAAAAGAAAAACAAAAAAAAACAATACAATGTAAAAAGCAGTACTATGTAGGAATAAATC
CTGCCTCAATTGCAGAAAAAAAAAAGGTATGCGTAATCTAAATAAGATACTATGTATTGAT
ATTTCATATCACTAACTTCTCTCAGATCAAATATGATTTAAAGGAGCGATTTCTGGACTG
TACAAAAAAAA

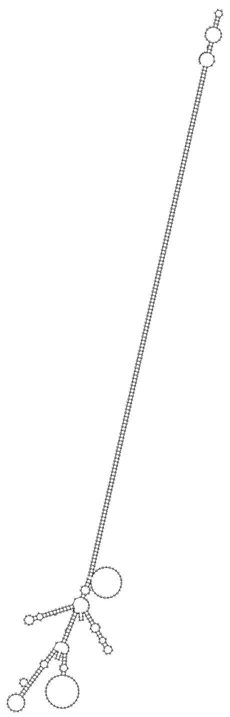

29 16965_130_7_-1 . . . 17016_121_7_1

16965_130_7_-1

TCGGAAATCATGTTTATGTCAAAATTTACTAGCCATTCCAATAATGTATTTATTATTAAA
TATATATATACTTCAAGGTCTGATGGGGGTGCTGTAGCATTTGCGATGTAGCGATAGCAA
TTGAAAATAA

17016_121_7_1

ACCTTGAAGTATATATATATTTAATAATAAATACATTATTGGAATGGCTAGTAAATTTTG
ACATAAACATGATTTCCGAACGAATTAAAATACAGTTGATTTAGGAGAAAGTATCAGTAC
A

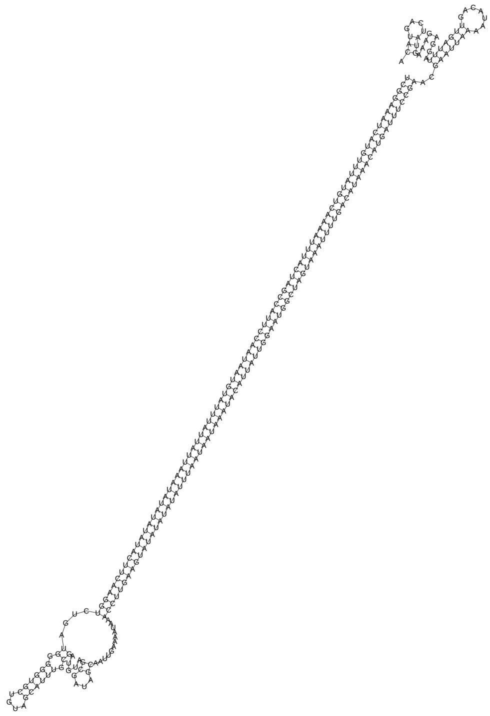

30 166663_122_7_-1 . . . 166622_123_7_1

166663_122_7_-1

ATTTCAGAATTGTAATATCGGACGGCAATACTAGTGTAATCTGAACCACAAGGTTAGTCT
TAGCTGGGTAAAACACCTGGCGAAAGAAGAAAAAAAAATAAGGAATCTGGATGATTATAAA
CT

166622_123_7_1

TGTATGCCCTTTTCATGTATAAAAGAAGGGTAATTTAAAAAAGTTTATAATCATCCAGAT
TCCTTATTTTTTTTCTTCTTTCGCCAGGTGTTTTACCCAGCTAAGACTAACCTTGTGGTT
CAG

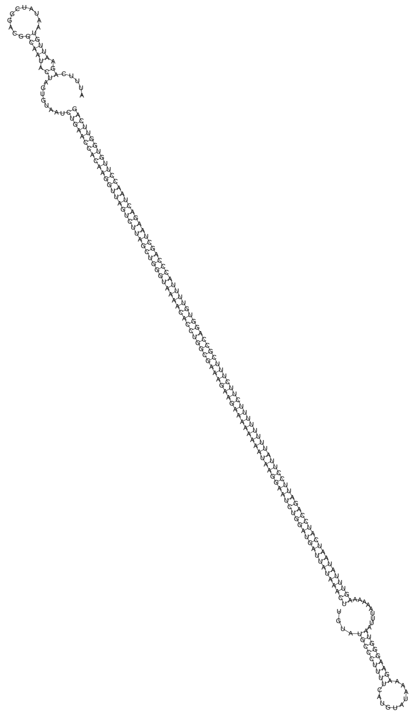

31 884266_119_7_-1 . . . 884226_119_7_1

884266_119_7_-1

GTAACCCGTACATGCCCAAAATAGGGGGCGGGTTACACAGAATATATAACATCGTAGGTG
TCTGGGTGAACAGTTTATTCCTGGCATCCACTAAATATAATGGAGCCCGCTTTTTAAGC

884226_119_7_1

AATATTTTGGTGCTGGGATTCTTTTTTTTTCTGGATGCCAGCTTAAAAAGCGGGCTCCAT
TATATTTAGTGGATGCCAGGAATAAACTGTTCACCCAGACACCTACGATGTTATATATT

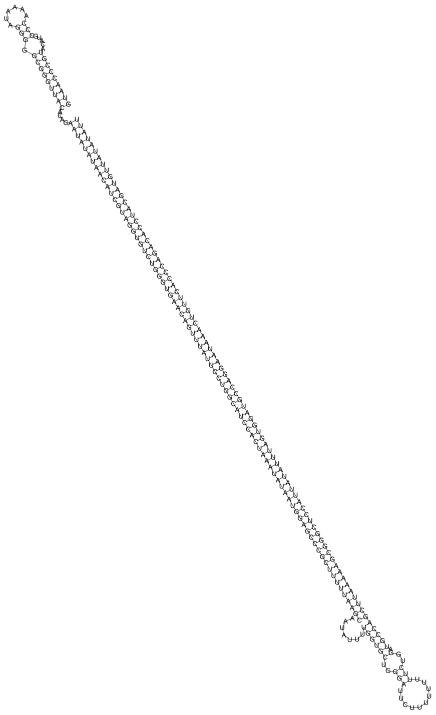

32 5973_130_8_-1 . . . 1269_305_4_1

5973_130_8_-1

TGGTTTACATTTAAGGTGCGACCAGCAATGTCACTGCTCGTACAGCGGTTACCGCTTGAA
TGAAATACATATTTTAAGCGTAGATTTCCTGTGACAAAACTGTGTAAACATCTAGTAGAA
AATAGACGTA

1269_305_4_1

CTATTGACATGGTATCGAAAGGTTGTCCACATTGGGAAGTAACTTGGTTCTATGAATCTT
CATGTCAGATACGTAGGACAGACTCTTTCCTGTGTAAATATTTGTGACAGCTACGTCTAT
TTTCTACTAGATGTTTACACAGTTTTGTCACAGGAAATCTACGCTTAAAATATGTATTTC
ATTCAAGCGGTAACCGCTGTACGAGCAGTGACATTGCTGGTCGCACCTTAAATGTAAACC
AACGTTACGGCACACCGTGATGTACCCGCATTAAAGTTTTGTAAATTCGTTATTACGATT
ATTGA

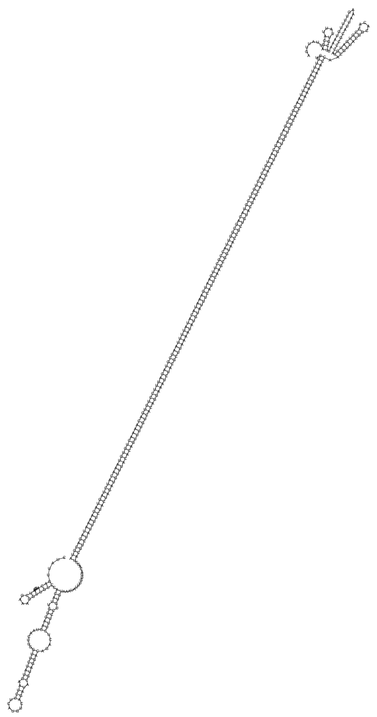

33 50784_119_8_-1 . . . 50743_119_8_1

50784_119_8_-1

TCGTAGATTGGCATCCCTCACCCGTCTGAAATGATTTTATAAGCCTCCGTCATCTCGGAC
GTAATTTTAGAAACTCCGAGCTGGACGGCTATTTTATTTTCTGCGCCGTAATCTGTGAA

50743_119_8_1

AATAATCTAAATACCGCGTTCGTGCGAGACATTCTCTGTGATTCACAGATTACGGCGCAG
AAAATAAAATAGCCGTCCAGCTCGGAGTTTCTAAAATTACGTCCGAGATGACGGAGGCT

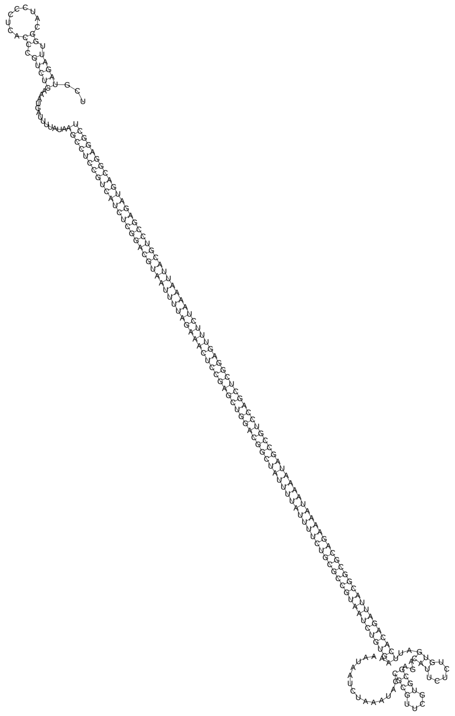

34 236637_122_8_-1 . . . 236597_120_8_1

236637_122_8_-1

TGCGAGTGAATAGGGAGAACCAAATAAATAGGGAGAAACTTGCTTTTTCTTCTATTTGAG
TAACAACTAGGTTGCCAAGAATATATATATTCTGCCAACTACCACGTTGTTACGGTAGTG
AG

236597_120_8_1

TAAAAGTATTTTAGTTTGTTGTTGTAATTTCGTCTTTTCTCTCACTACCGTAACAACGTG
GTAGTTGGCAGAATATATATATTCTTGGCAACCTAGTTGTTACTCAAATAGAAGAAAAAG

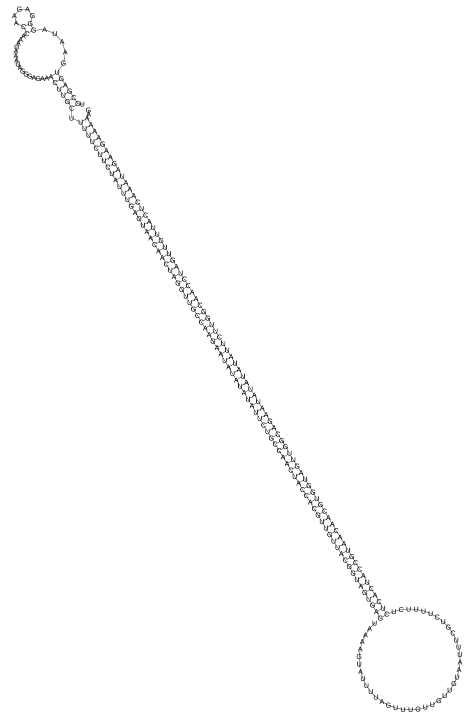

35 319990_120_8_-1 . . . 319950_120_8_1

319990_120_8_-1

TTAGCGCTTTCTCAGGATTTGCGCTTCGCACGGTTTTTTCTTCTAGGCGCGTTTATAAGA
AGAAAAAAATAACGGAAAAAAACAAATGGCGAGGTTTGGTAACTGCGCCATTTGCTTTCTTT

319950_120_8_1

CAAGGTTAGCGCTTTACGGACTTTATTGCTATTCTAGTTCAAAGAAAGCAAATGGCGCAG
TTACCAAACCTCGCCATTTGTTTTTTCCGTTATTTTTTCTTCTTATAAACGCGCCTAGAA

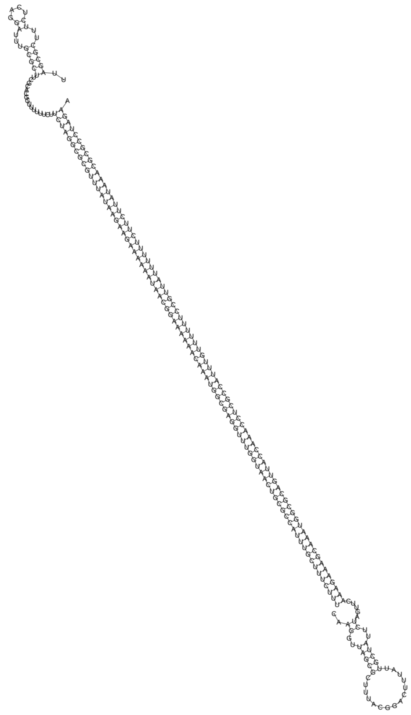

36 117760_117_9_-1 . . . 117720_237_9_1

117760_117_9_-1

TGATAAACTTTTTCTTTTCTCAGTGCCAAATTGGTGTCTTCTTTTTTTGCCACGGGGCGG
GTACTAGATAGTCACGTGAATGTATACACGTGACCCTAATGCTCTTTGAAAAATTTT

117720_237_9_1

GCCAGCTATATTTCCATATTCCTTAGTTAAAGCTCATCGCAAAATTTTTCAAAGAGCATT
AGGGTCACGTGTATACATTCACGTGACTATCTAGTACCCGCCCCGTGGCAAAAAAAGAAG
ACACCAATTTGGCACTGAGAAAAGAAAAAGTTTATCAATAACGAAAACGAAACTTAAGAG
GAAAAAGAGTATAGAGAAAAACAAAGGCAATCAGCGGCTAATTTGGTGGAAATAACA

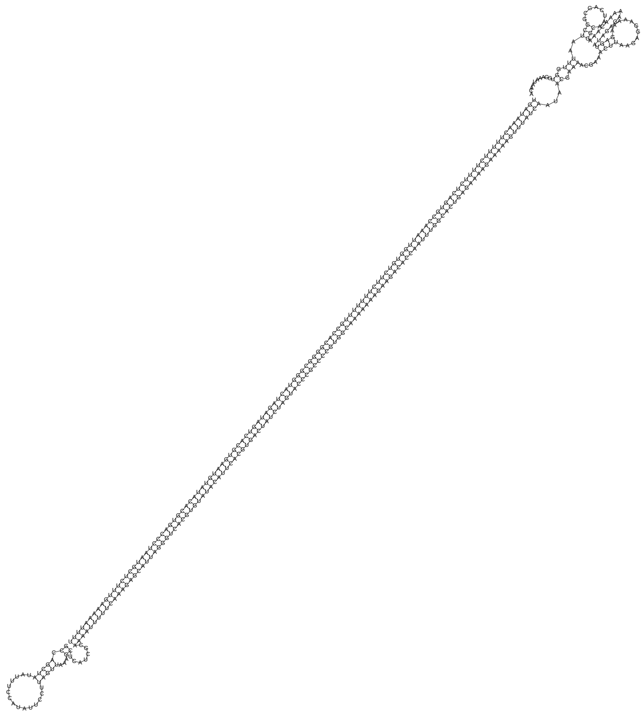

37 2096_297_0_-1 . . . 62832_137_0_1

2096_297_0_-1

TCTATAAAGAAATGAGATTAGATGATATTGAAATGAAATAAAATGAGATGAGATGAATTG
GGGCCCGGAAAGGGACCGAACCCCTCATAATAATTAATTTAATATATATTATAATAAAAC
TTATTATATATATATTTTTATATATATATATATATATTTTTTTTATATATTATATAATAAATAT
TACTTCTTATTAAAATTCCTTATTTTTATTATTTTTAATAATTAAATTCATATAGTTATT
ATAATATATAGATAAACTCCTTTCGGGGTTCCGGCTCCCGTGGCCGGGCCCCGGAAC

62832_137_0_1

ATATATTATTAATTTAATAATTATTATAATAGTTCCGGGGCCCGGCCACGGGAGCCGGAA
CCCCGAAAGGAGTTTATAAAAGATATATTTTTATATTATATTATATTATATTTAATAAAT
ATTACCTTTTTTTATTA

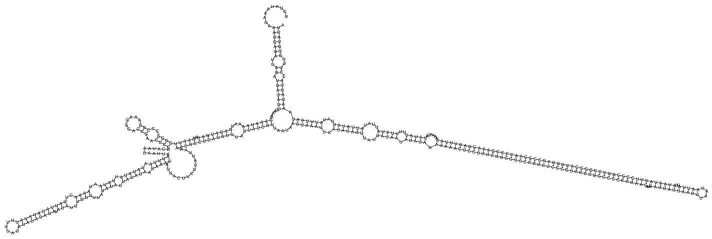

38 29783_136_0_-1 . . . 29726_260_0_1

29783_136_0_-1

TAAATATGAATATTATTAAAAAATATGTTATAATTTCTCCTTCCGGGGTTCCGGCTCCCG
TGGCCGGGCCCCGGAACTATATGATATAATAAATATATTATATAATAATCTTATATTTAT
TATGTTATATTATTTT

29726_260_0_1

TATAGTATTAAAAAAAAATAAAATATTTAATAAATATTATTATTAATAATATTTATTAAAA
ATAATATAACATAATAAATATAAGATTATTATATAATATATTTATTATATCATATAGTTC
CGGGGCCCGGCCACGGGAGCCGGAACCCCGGAAGGAGAAATTATAACATATTTTTTAATA
ATATTCATATTTATTTTATATACAAATAAATATATTTATTTAGAATAATAAAAAAAAATA
ATAAATAAATATATTATTAT

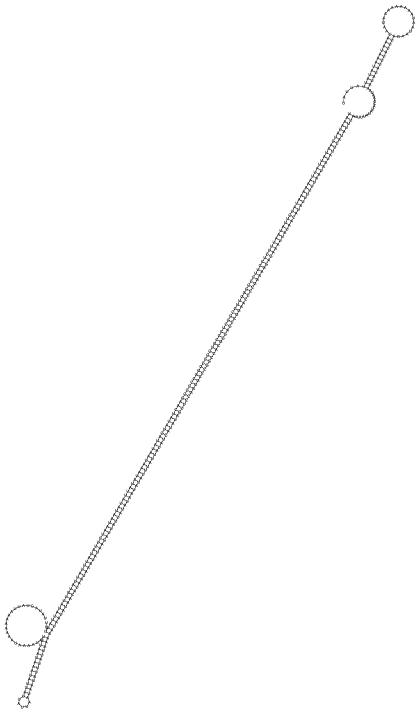

39 29783_136_0_-1 . . . 62832_137_0_1

29783_136_0_-1

TAAATATGAATATTATTAAAAAATATGTTATAATTTCTCCTTCCGGGGTTCCGGCTCCCG
TGGCCGGGCCCCGGAACTATATGATATAATAAATATATTATATAATAATCTTATATTTAT
TATGTTATATTATTTT

62832_137_0_1

ATATATTATTAATTTAATAATTATTATAATAGTTCCGGGGCCCGGCCACGGGAGCCGGAA
CCCCGAAAGGAGTTTATAAAAGATATATTTTTATATTATATTATATTATATTTAATAAAT
ATTACCTTTTTTTATTA

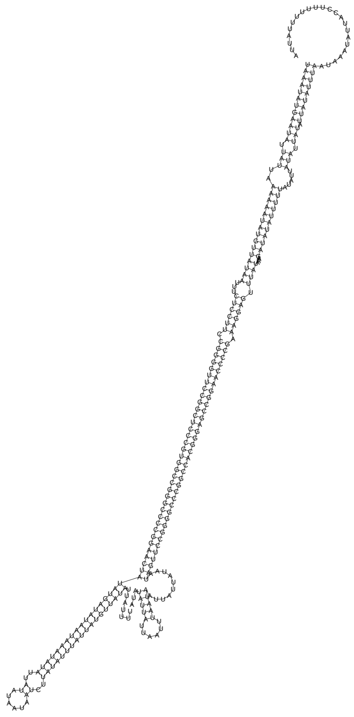

40 29783_136_0_-1 . . . 33146_467_0_-1

29783_136_0_-1

TAAATATGAATATTATTAAAAAATATGTTATAATTTCTCCTTCCGGGGTTCCGGCTCCCG
TGGCCGGGCCCCGGAACTATATGATATAATAAATATATTATATAATAATCTTATATTTAT
TATGTTATATTATTTT

33146_467_0_-1

AGAGGGATAATTATTTTATTATATTTATATACAATAGGATTAAACATTGCCAGTGAACAA
CGGGTAATGTTTGATCCGTATCATATTATATTATATTATATTATATTATATTATATAATT
AATAGTTCCGGGGCCCGGCACGGGAACCGGAACCCCGAAAGGAGGAGAAGATAATATTAT
TACTATTTTTCTTTATAAAAATAAAATAAAATAAAATAAAATAGTCCGGTAGAAAGAGAT
ATTATAACTTTATTAAATATATTAAATATATAATAATTATTATTCCATTTAAAATTACAA
TTTTCGTTTCAATTATAATACTTTTTAATTATATTATAATATATAATTATTTATAATAAT
AGTACGCTCCGAAGGAGTGAGGGACCCCCCCCTTACGGGGGGGAACCGAACCCCGCAGGA
GATATTTATTAATAATGAAATTATATTAATATATTATTATTATTATA

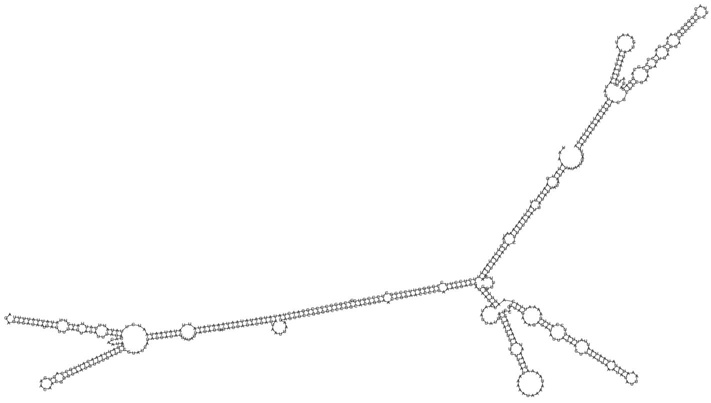

41 29783_136_0_-1 . . . 76206_186_0_1

29783_136_0_-1

TAAATATGAATATTATTAAAAAATATGTTATAATTTCTCCTTCCGGGGTTCCGGCTCCCG
TGGCCGGGCCCCGGAACTATATGATATAATAAATATATTATATAATAATCTTATATTTAT
TATGTTATATTATTTT

76206_186_0_1

TATATTTATAAAAATATTAATATTTTATTTAAAATAAATAATGATTAATTTATAAAATAT
ATATTAATTAAGTTTCGGGTCCCGGCTACGGGACCCGGAACCCCCGAGAGGAGTTATTAT
ATTTATAATTAAATCTTTAAATAATATATCTTAAATTATTATATTGATATTAATATTATA
TTGATA

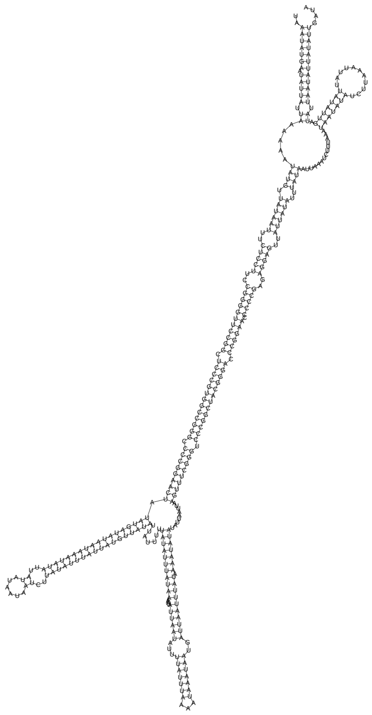

42 30507_136_0_-1 ... 32419_121_0_1

30507_136_0_-1

TATTTCTATTTTATATACATTATTATTATATTAATTAATATGATATTATAATGGTGGGGG
GTCCCAATTATTATTGAAAATAATAATTATTAATGGGACCCCAGATATCTTCTTGTTAAT
CATTTATTATTTTATT

32419_121_0_1

AATAAAATAATAAATGATAAACAAGAAGATATCCGGGTCCCAATAATAATTATTATTGAA
AATAATAATTGGGACCCCATATAGAATATAAATAATTAAATATATATATATAAATAATAA
T

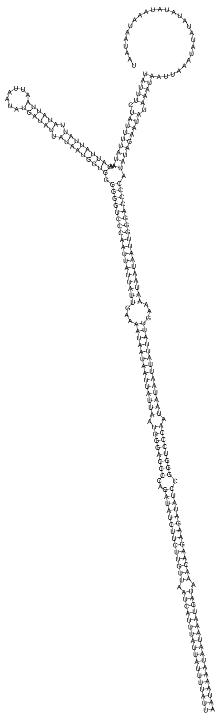

43 30507_136_0_-1 . . . 45142_123_0_1

30507_136_0_-1

TATTTCTATTTTATATACATTATTATTATATTAATTAATATGATATTATAATGGTGGGGG
GTCCCAATTATTATTGAAAATAATAATTATTAATGGGACCCCAGATATCTTCTTGTTAAT
CATTTATTATTTTATT

45142_123_0_1

TAAATAATATAATAAATGATAAACAAGAAGATATCCGGGTCCCAATAATAATTATTATTG
AAAATAATAATTGGGACCCCCATCTAAAATATATATATAACTAATAATATATTATATATA
TTA

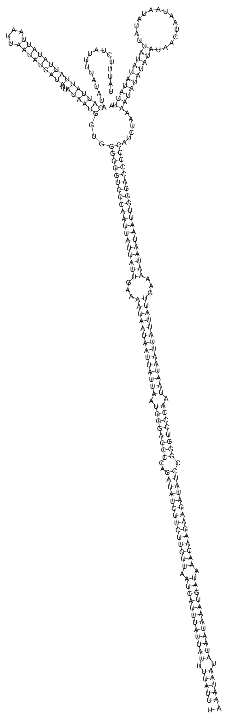

44 33146_467_0_-1 . . . 34913_97_0_-1

33146_467_0_-1

AGAGGGATAATTATTTTATTATATTTATATACAATAGGATTAAACATTGCCAGTGAACAA
CGGGTAATGTTTGATCCGTATCATATTATATTATATTATATTATATTATATTATATAATT
AATAGTTCCGGGGCCCGGCACGGGAACCGGAACCCCGAAAGGAGGAGAAGATAATATTAT
TACTATTTTTCTTTATAAAAATAAAATAAAATAAAATAAAATAGTCCGGTAGAAAGAGAT
ATTATAACTTTATTAAATATATTAAATATATAATAATTATTATTCCATTTAAAATTACAA
TTTTCGTTTCAATTATAATACTTTTTAATTATATTATAATATATAATTATTTATAATAAT
AGTACGCTCCGAAGGAGTGAGGGACCCCCCCCTTACGGGGGGGAACCGAACCCCGCAGGA
GATATTTATTAATAATGAAATTATATTAATATATTATTATTATTATA

34913_97_0_-1

TTTATTAATATAATTTATATATTTATCTTATTCCTCCTTTCGGGGTTCCGGCTCCCGTGG
CCGGGCCCCGGAACTATTAATATATAAATTGAATTAT

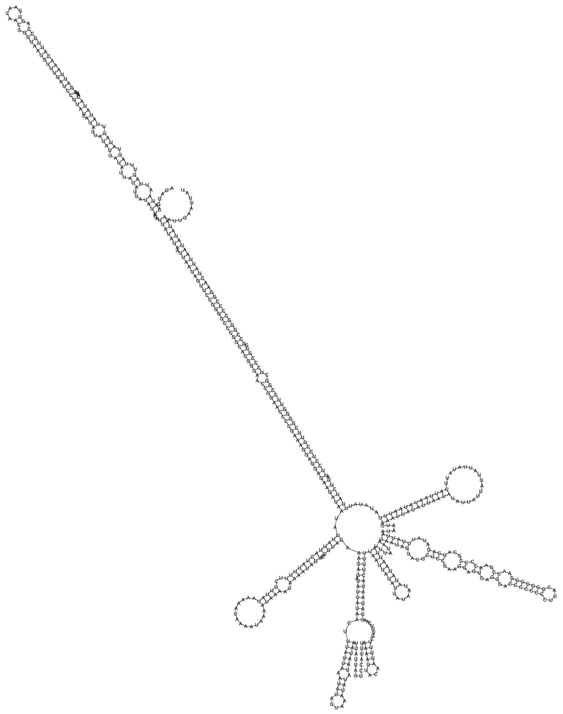

45 33146_467_0_-1 ... 29783_136_0_-1

33146_467_0_-1

AGAGGGATAATTATTTTATTATATTTATATACAATAGGATTAAACATTGCCAGTGAACAA
CGGGTAATGTTTGATCCGTATCATATTATATTATATTATATTATATTATATTATATAATT
AATAGTTCCGGGGCCCGGCACGGGAACCGGAACCCCGAAAGGAGGAGAAGATAATATTAT
TACTATTTTTCTTTATAAAAATAAAATAAAATAAAATAAAATAGTCCGGTAGAAAGAGAT
ATTATAACTTTATTAAATATATTAAATATATAATAATTATTATTCCATTTAAAATTACAA
TTTTCGTTTCAATTATAATACTTTTTAATTATATTATAATATATAATTATTTATAATAAT
AGTACGCTCCGAAGGAGTGAGGGACCCCCCCCTTACGGGGGGGAACCGAACCCCGCAGGA
GATATTTATTAATAATGAAATTATATTAATATATTATTATTATTATA

29783_136_0_-1

TAAATATGAATATTATTAAAAAATATGTTATAATTTCTCCTTCCGGGGTTCCGGCTCCCG
TGGCCGGGCCCCGGAACTATATGATATAATAAATATATTATATAATAATCTTATATTTAT
TATGTTATATTATTTT

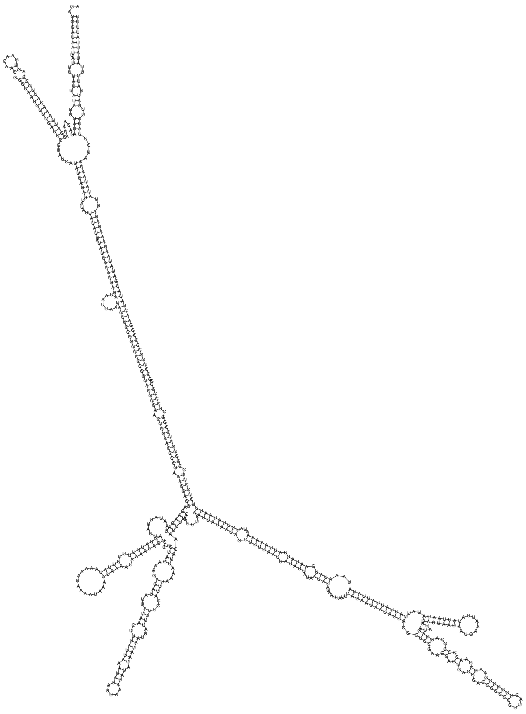

46 34913_97_0_-1 ... 62832_137_0_1

34913_97_0_-1

TTTATTAATATAATTTATATATTTATCTTATTCCTCCTTTCGGGGTTCCGGCTCCCGTGG
CCGGGCCCCGGAACTATTAATATATAAATTGAATTAT

62832_137_0_1

ATATATTATTAATTTAATAATTATTATAATAGTTCCGGGGCCCGGCCACGGGAGCCGGAA
CCCCGAAAGGAGTTTATAAAAGATATATTTTTATATTATATTATATTATATTTAATAAAT
ATTACCTTTTTTTATTA

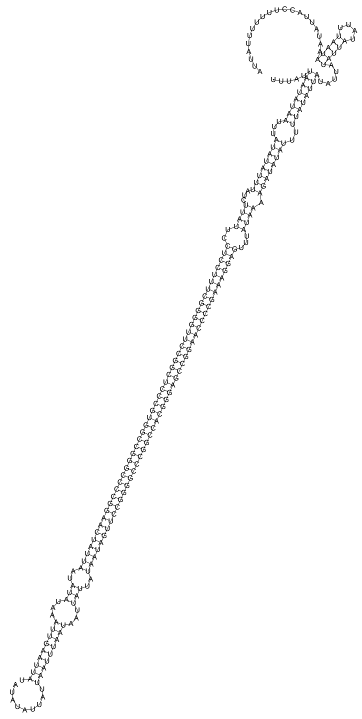

47 34913_97_0_-1 . . . 33146_467_0_-1

34913_97_0_-1

TTTATTAATATAATTTATATATTTATCTTATTCCTCCTTTCGGGGTTCCGGCTCCCGTGG
CCGGGCCCCGGAACTATTAATATATAAATTGAATTAT

33146_467_0_-1

AGAGGGATAATTATTTTATTATATTTATATACAATAGGATTAAACATTGCCAGTGAACAA
CGGGTAATGTTTGATCCGTATCATATTATATTATATTATATTATATTATATTATATAATT
AATAGTTCCGGGGCCCGGCACGGGAACCGGAACCCCGAAAGGAGGAGAAGATAATATTAT
TACTATTTTTCTTTATAAAAATAAAATAAAATAAAATAAAATAGTCCGGTAGAAAGAGAT
ATTATAACTTTATTAAATATATTAAATATATAATAATTATTATTCCATTTAAAATTACAA
TTTTCGTTTCAATTATAATACTTTTTAATTATATTATAATATATAATTATTTATAATAAT
AGTACGCTCCGAAGGAGTGAGGGACCCCCCCCTTACGGGGGGGAACCGAACCCCGCAGGA
GATATTTATTAATAATGAAATTATATTAATATATTATTATTATTATA

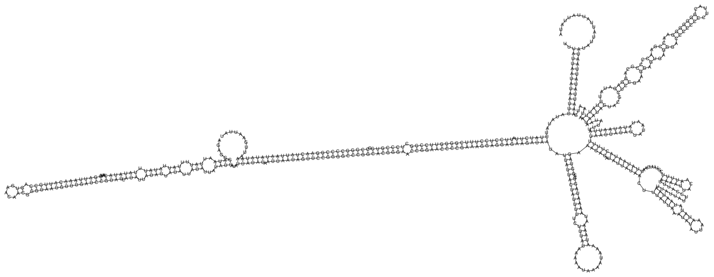

48 34913_97_0_-1 . . . 29726_260_0_1

34913_97_0_-1

TTTATTAATATAATTTATATATTTATCTTATTCCTCCTTTCGGGGTTCCGGCTCCCGTGG
CCGGGCCCCGGAACTATTAATATATATAAATTGAATTAT

29726_260_0_1

TATAGTATTAAAAAAAAATAAAATATTTAATAAATATTATTATTAATAATATTTATTAAAA
ATAATATAACATAATAAATATAAGATTATTATATAATATATTTATTATATCATATAGTTC
CGGGGCCCGGCCACGGGAGCCGGAACCCCGGAAGGAGAAATTATAACATATTTTTTAATA
ATATTCATATTTATTTTATATACAAATAAATATATTTATTTAGAATAATAAAAAAAAATA
ATAAATAAATATATTATTAT

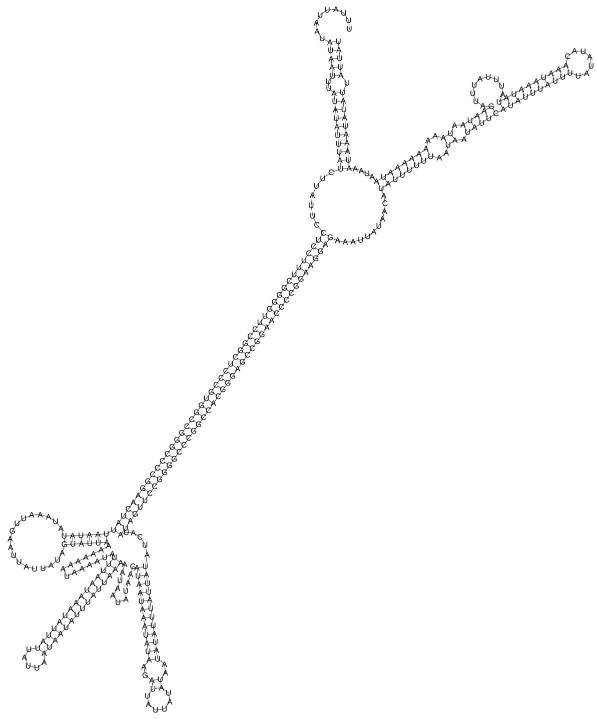

49 68417_160_1_1 . . . 68377_120_1_-1

68417_160_1_1

AAACGGGTAAAACAGCCATGCACCGCAGCACGTCGACGAGGTGATATTTCCAATTTGGGA
AATTTCCCAAATCAGTAATGTAGCCTCTACGGGTGTCTCTGTCAGCCCCGTGGTCGCCAG
CACAGAATGTATCGTACCCCTGAAGGTAGTTTTTTACCGC

68377_120_1_-1

CATTACTGATTTGGGAAATTTCCCAAATTGGAAATATCACCTCGTCGACGTGCTGCGGTG
CATGGCTGTTTTACCCGTTTAGGAAAAAAACTCGGCGGGTTTTCTTGACGGGCAAATGTCG

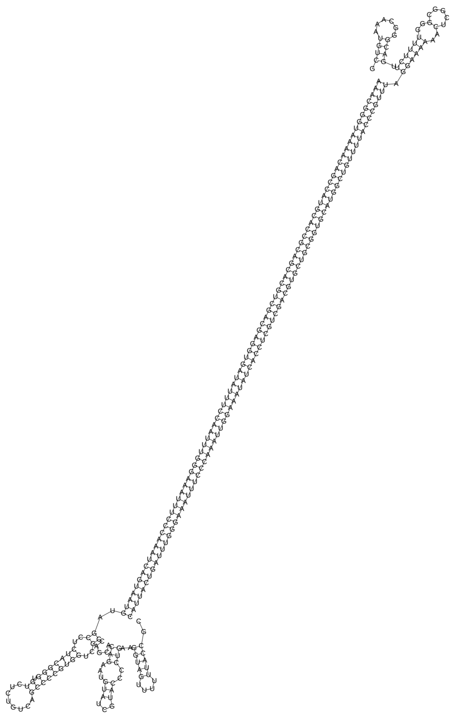

50 177336_121_1_1 . . . 177376_120_1_-1

177336_121_1_1

AAAAGTGCCTATAGGGCTGCAGCTGCAGTTTTGGCCAAGAAATAGAACCAAAGCCAAATT
TATTTTGGGCCCTCGTTCAAGGGCCATCTCACCCTTGGCACTAAACGGTTAGTAGGAGGG
A

177376_120_1_-1

TTAATTTTTCATTGTTTCTAATTTGGGAAAAGTCCGATTTCCCTCCTACTAACCGTTTAG
TGCCAAGGGTGAGATGGCCCTTGAACGAGGGCCCAAAATAAATTTGGCTTTGGTTCTATT

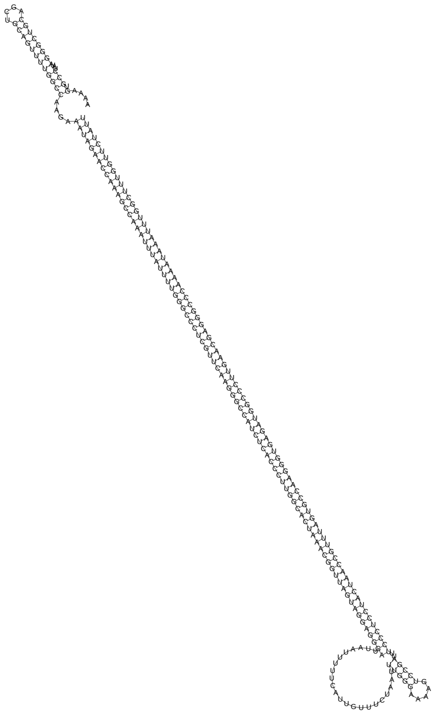

51 177336_121_1_1 . . . 99910_119_6_-1

177336_121_1_1

AAAAGTGCCTATAGGGCTGCAGCTGCAGTTTTGGCCAAGAAATAGAACCAAAGCCAAATT
TATTTTGGGCCCTCGTTCAAGGGCCATCTCACCCTTGGCACTAAACGGTTAGTAGGAGGG
A

99910_119_6_-1

GTTTTTTCTCCTAGCAACCGTTTAGTGCCAAGGGTTAGGCAATTGAACGAGGCCAAGACA
ATATTGGCTTTGCTTCTATTACTTGGCTAACATTGTGTCTGCAGGTCGAAAGGCACCTT

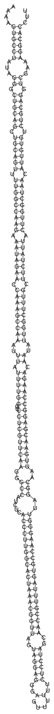

52 144176_119_11_1 . . . 144137_119_11_-1

144176_119_11_1

GTCGACTGAAATAGAAGGGATAAATCATGCATCTCCAGGATTATCCCTACTCCATTCATT
ACAACATGCGCCAAATCAAGCCTATATAAGATTCTCGTCATTTAGCATGCTCTATTGAT

144137_119_11_-1

CTTGATTTGGCGCATGTTGTAATGAATGGAGTAGGGATAATCCTGGAGATGCATGATTTA
TCCCTTCTATTTCAGTCGACGGCGAAATATGCCAAATTTAGAAAGCCCTCGGCCTTTGA

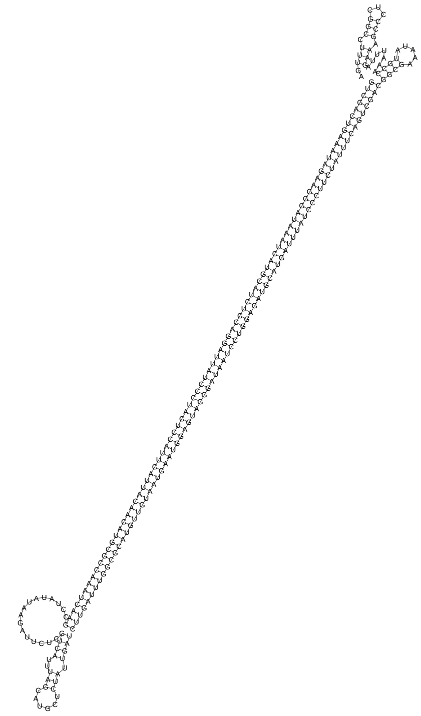

53 289817_120_12_1 . . . 289857_120_12_-1

289817_120_12_1

GGAGATATCTGCGCCGTTCAGGGGTCCATGTGCCTTGGACGATATTAAGGCAGAAGGCAG
TATCGGGGCGGATCACTCCGAACCGAGATTAGTTAAGCCCTTCCCATCTCAAGATGGGGA

289857_120_12_-1

TGCACAGTTAACTTTCTAGCAGGAGTATAATGCCATTTGCTCCCCATCTTGAGATGGGAA
GGGCTTAACTAATCTCGGTTCGGAGTGATCCGCCCCGATACTGCCTTCTGCCTTAATATC

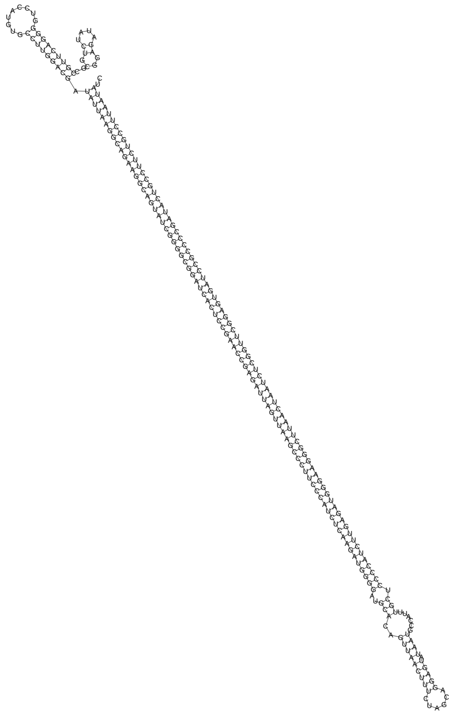

54 534573_158_12_1 . . . 534534_117_12_-1

534573_158_12_1

GTTTTTCCAAGTCATCTCAAAACAATGACCTATTTTATTTCTAGATAAACCCTGGTGTAA
GCAGGGTTAATTTTGTCCCAAACGGGCAAAATATAAATACCCCTTTCGGGAAATAAACTA
AAAAGAGTTCTAATTAGCCAATTGGCAAGAAAGCTCGA

534534_117_12_-1

GGACAAAATTAACCCTGCTTACACCAGGGTTTATCTAGAAATAAAATAGGTCATTGTTTT
GAGATGACTTGGAAAAACTTAACCCTGATGTGACTGTAACATAACATCTAAGAAAAA

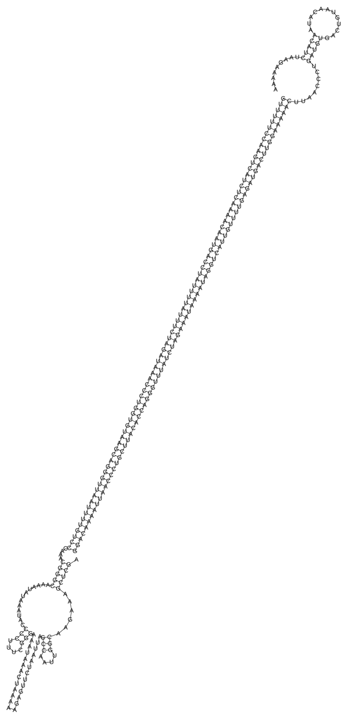

55 602614_121_12_1 . . . 602654_123_12_-1

602614_121_12_1

ATCGATTTCGGTGACTAGTTAGTAGATGATGTAATCGTAGACGCAGGGAACCCTTGCTTT
TCCCCAATCGTAACAAAAAAAAACAAGGGGAAATAAACTATTAGATAGTACGAGTTCGTT
A

602654_123_12_-1

GTAATCATTCGTTCTTCGACTTTTATAGCCTTTTTTTTAACTTAACGAACTCGTACTATC
TAATAGTTTATTTCCCCTTGTTTTTTTTTGTTACGATTGGGGAAAAGCAAGGGTTCCCTG
CGT

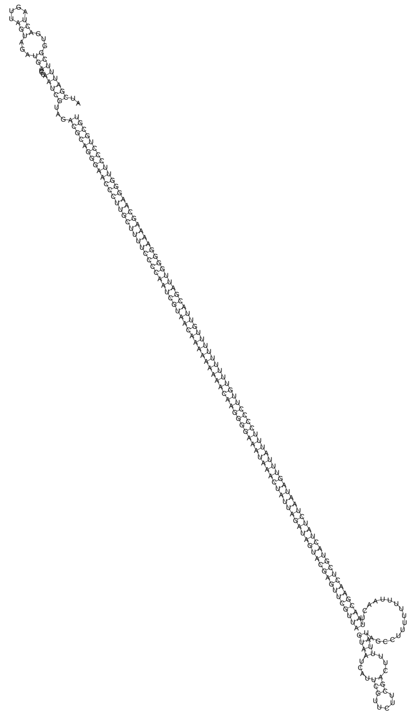

56 789982_162_12_1 . . . 789942_120_12_-1

789982_162_12_1

AAAACGGAAAAAAAATCTAAAAAAAGAAATTAATTGAGAGATCTCACGGAAATGCCGCGAG
GAATGTTTCTCGAGGCTGAGCGGCGTGGTCTGTGCAAAAAAAATGGCAATTTTTTTGTAGG
AGTTTGCATTGGGCCATTCAGAAGGAGCACCGTTAGATGGGA

789942_120_12_-1

CTCAGCCTCGAGAAACATTCCTCGCGGCATTTCCGTGAGATCTCTCAATTAATTTCTTTT
TTTAGATTTTTTTCCGTTTTCCGTTTGAATAATTTCGCGTTTTCGTAGAGCGCGACGGCC

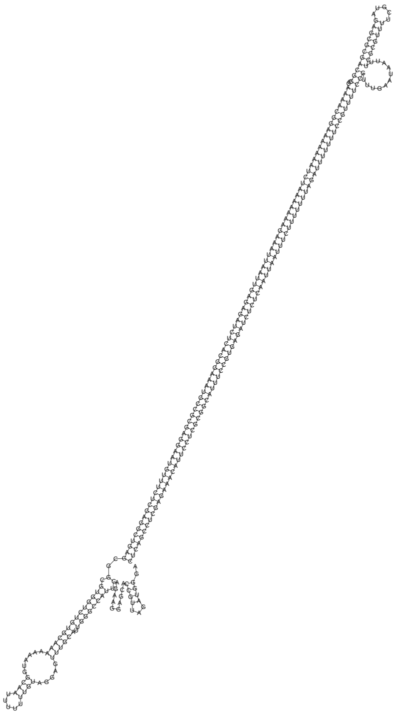

57 837505_46_12_1 . . . 837505_46_12_1

837505_46_12_1

TACACGCACACATATATATATATATATATATATGTATATGTGTATATA

837505_46_12_1

TACACGCACACATATATATATATATATATATATGTATATGTGTATATA

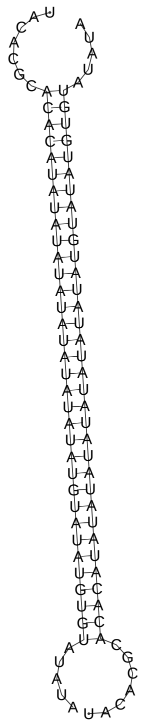

58 481942_123_13_1 . . . 481901_121_13_-1

481942_123_13_1

GCTACTGTTCAATAAATATTGTTGTAATGAAGACGGTCCAACTGTACAAATACAGCAAAC
TGTCATATATAAGGAGTCTTATGTGACAGCACTTGCGTTATTGTCAGCCGGAGTATGTCT
TTG

481901_121_13_-1

AAGACTCCTTATATATGACAGTTTGCTGTATTTGTACAGTTGGACCGTCTTCATTACAAC
AATATTTATTGAACAGTAGCTTGTAATAGGCCGGCATTTTTTTGGTTAATAACAATGCCA
G

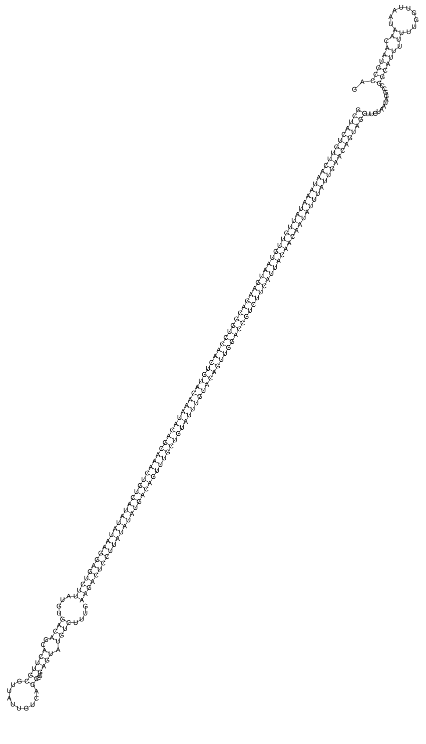

59 861651_124_13_1 . . . 861691_124_13_-1

861651_124_13_1

CAGTACACTTCGGTAGCAACCTTCGTTTGTGATTGTCTTGGTAATTGCTTCCAACAACTT
TATCCATCATTGAGACAGGGGCCATATCACCCGCGGGGTCTCAAAGAAGGGGCCCACTAA
TAAA

861691_124_13_-1

TTATTTCGGAGCTTTTTTGCTGGCATTCTTATCTCGTGCTTTTATTAGTGGGCCCCTTCT
TTGAGACCCCGCGGGTGATATGGCCCCTGTCTCAATGATGGATAAAGTTGTTGGAAGCAA
TTAC

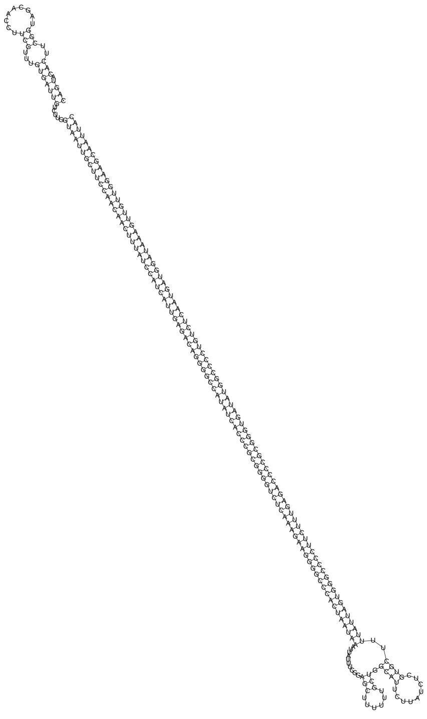

60 87556_120_14_1 . . . 87516_120_14_-1

87556_120_14_1

CACGAACAAGAATTTCGTGTTAAAAATCGCGTCCGGCGCGAAATTTTTCGCGGAGGCATG
CGACGCAAAAGCGACTCGAAATGTCGGGAGCCAAATGAGGCTACAAGGCTGTGGGCAGAT

87516_120_14_-1

TTCGAGTCGCTTTTGCGTCGCATGCCTCCGCGAAAAATTTCGCGCCGGACGCGATTTTTA
ACACGAAATTCTTGTTCGTGCCGCTGTTGCCCTTTTGGGAAATATTTCCCGATCTGGCAT

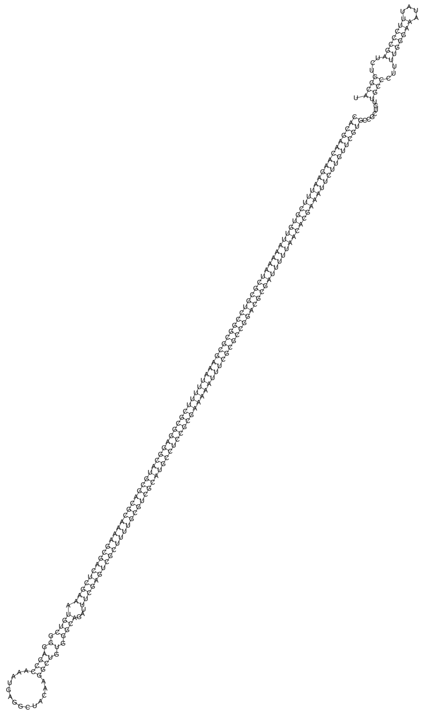

61 405776_124_14_1 . . . 405735_125_14_-1

405776_124_14_1

AAATTCAATAACTATCACTATATACGCAACAGTATTACCCTACATTGCTATCGGCTCAAT
GGAAATCCCCATATCATAGCTTCCATTGGGCCGATGAAGTTAGTCGACGGATAGAAGCGG
TTGT

405735_125_14_-1

GGAAGCTATGATATGGGGATTTCCATTGAGCCGATAGCAATGTAGGGTAATACTGTTGCG
TATATAGTGATAGTTATTGAATTTTATTACCCTGCGGGAATATTGAGACATCACTAAGCA
CGAAT

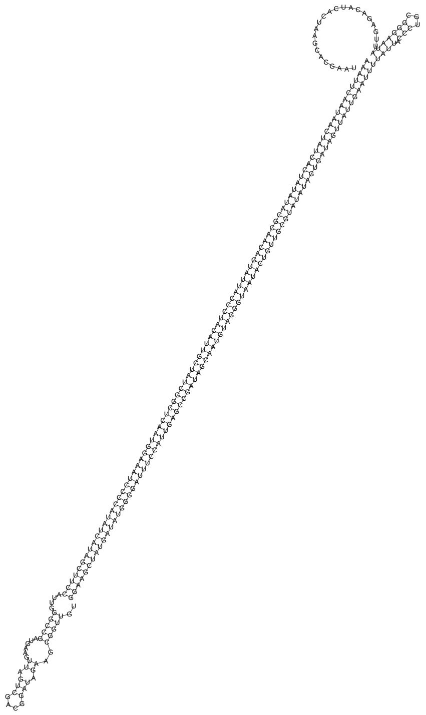

62 1004646_121_15_1 . . . 1004686_117_15_-1

1004646_121_15_1

TTGACTATTTGACTCAAATTACCAAAATCATAACTTTTCTTTTCAGCATTCCATTATTGA
AGGCTTTAAAATAATAGTTTCTTTTTTCCTTATTATTTTATTTTTTCCTTTACATTGGAA
T

1004686_117_15_-1

AGAAAATAAAAAAAAATTAAAACTCAAAATAAAATGTATTCCAATGTAAAGGAAAAAATAA
AATAATAAGGAAAAAAGAAACTATTATTTTAAAGCCTTCAATAATGGAATGCTGAAA

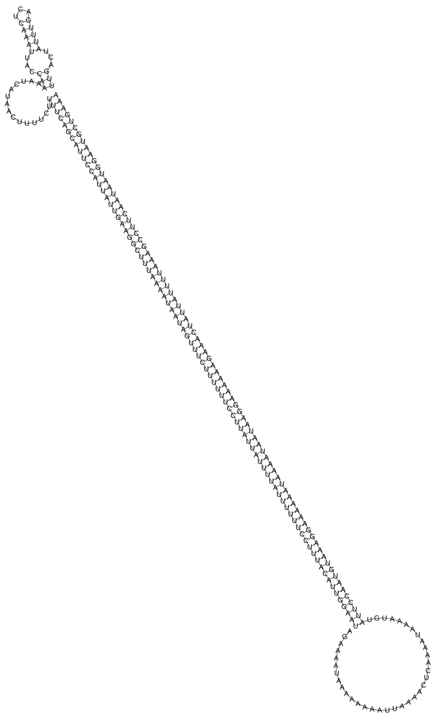

63 1057877_121_15_1 . . . 1057918_121_15_-1

1057877_121_15_1

AATTAACTGAATTTTTTTTCTGGATTGATGTTCTTTCCAATTGTTGTGGTAATCCACAGC
TGGTATAACATAAAAAGAAGTTTTTTGTAAAAAAAAAAAAATTTCTCAGTAAGCATATAAG
C

1057918_121_15_-1

AATAGAATAGCGTGTAGTTGTTAAAATTTTATTTCACTGAGGCTTATATGCTTACTGAGA
AATTTTTTTTTTTTACAAAAAACTTCTTTTTATGTTATACCAGCTGTGGATTACCACAAC
A

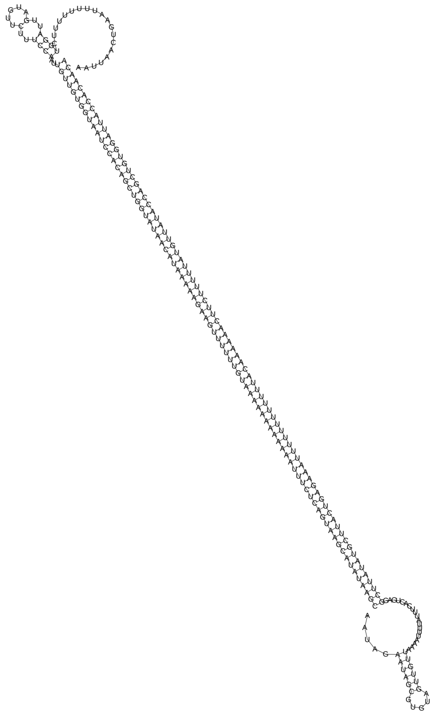

64 425897_158_16_1 . . . 425979_122_16_-1

425897_158_16_1

ATGCTGGCGTCCTTAGCCTCACGCCTACTAAACAAGAGCATCTTACTCTCACCATCCATT
TATATACATATATAAGTACGTAAAAATAGATGTGTATAAGTCGTTCTATCTATAACGCGC
CCCGAAGTTGTTTAGTATAACAAAAACGGAAAAAAAAA

425979_122_16_-1

TCTTTCTGTCAAACTAGCGCGCTTTTGGCAATCTCCATTTTCCTTCTTTTTTTTTCCGTT
TTTGTTATACTAAACAACTTCGGGGCGCGTTATAGATAGAACGACTTATACACATCTATT
TT

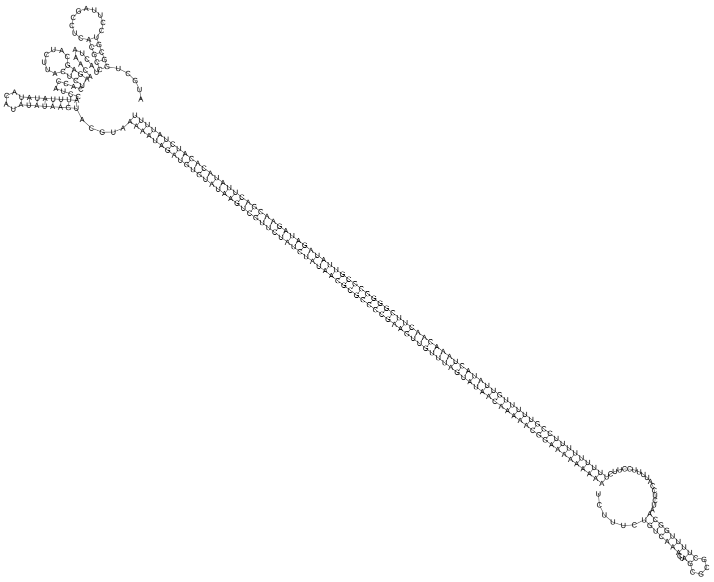

65 812831_165_16_1 . . . 812791_123_16_-1

812831_165_16_1

AGCGAGGAAAAACCTCGAGATGGAAAACCAGTTTATGGAAAACTTCAAAATAACTCGCCC
GCCTTATCGCGGGCAAACCAGCTACGAGAGATAAGGATTTGGCGCCGAGATAAGGTGGAG
ATGTTTCTCCGCCGCGCGGCACTTTTTGCCGGAACTGGAACTGGT

812791_123_16_-1

AGCTGGTTTGCCCGCGATAAGGCGGGCGAGTTATTTTGAAGTTTTCCATAAACTGGTTTT
CCATCTCGAGGTTTTTCCTCGCTTTCCACGCTATGACCCTTTTTAGTTAAGGTACCCGAT
GGC

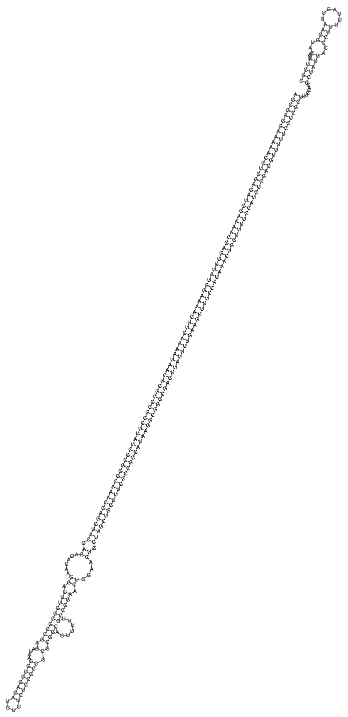

66 830605_137_16_1 . . . 830482_202_16_-1

830605_137_16_1

AAATGAAAAAAAATACCAGAAAAAAAAATAACAAATCATATGCACTTCTTCATTAACGATTA
TGAAGAAGTCTTATTTCTAACTAATATTATTATTATTATTATTATTATTATCATTCACCC
CGCTTTCTTTTATTATA

830482_202_16_-1

TAGAAATAAGACTTCTTCATAATCGTTAATGAAGAAGTGCATATGATTTGTTATTTTTTT
TCTGGTATTTTTTTTCATTTTTTTTCGGGTTTTCCTTGGGTATAATTTTTTGTTTCTTCCGA
AAGACGGAAAGGGATGAGGGAAAACGGAGTTTTCTTATTTGCTTTCTTTTTTCCCTAATT
TATTCACGGTGTCCATTTATTA

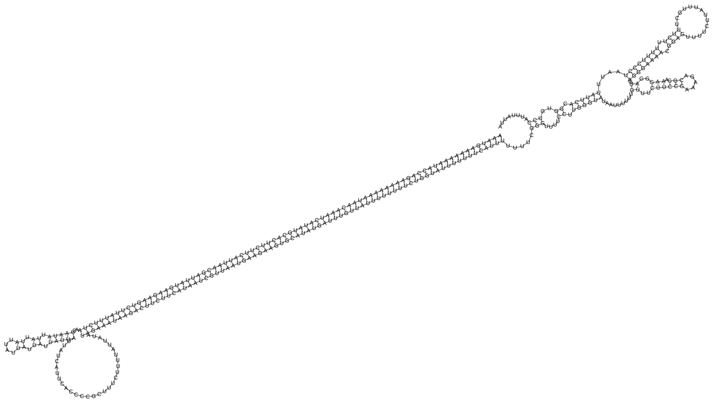

67 1269_305_4_1 ... 1370_244_4_-1

1269_305_4_1

CTATTGACATGGTATCGAAAGGTTGTCCACATTGGGAAGTAACTTGGTTCTATGAATCTT
CATGTCAGATACGTAGGACAGACTCTTTCCTGTGTAAATATTTGTGACAGCTACGTCTAT
TTTCTACTAGATGTTTACACAGTTTTGTCACAGGAAATCTACGCTTAAAATATGTATTTC
ATTCAAGCGGTAACCGCTGTACGAGCAGTGACATTGCTGGTCGCACCTTAAATGTAAACC
AACGTTACGGCACACCGTGATGTACCCGCATTAAAGTTTTGTAAATTCGTTATTACGATT
ATTGA

1370_244_4_-1

AAGGGTATCCATTTCATTTCCTGTTTTCTATCTAGCCAATTCAATAATCGTAATAACGAA
TTTACAAAACTTTAATGCGGGTACATCACGGTGTGCCGTAACGTTGGTTTACATTTAAGG
TGCGACCAGCAATGTCACTGCTCGTACAGCGGTTACCGCTTGAATGAAATACATATTTTA
AGCGTAGATTTCCTGTGACAAAACTGTGTAAACATCTAGTAGAAAATAGACGTAGCTGTC
ACAA

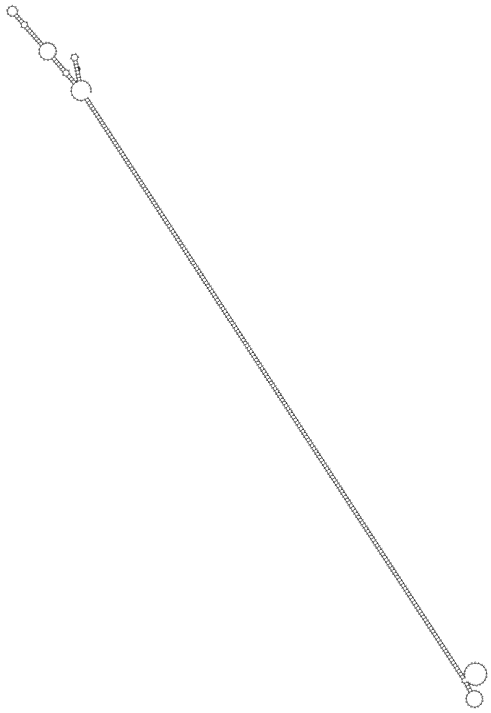

68 1269_305_4_1 . . . 5973_130_8_-1

1269_305_4_1

CTATTGACATGGTATCGAAAGGTTGTCCACATTGGGAAGTAACTTGGTTCTATGAATCTT
CATGTCAGATACGTAGGACAGACTCTTTCCTGTGTAAATATTTGTGACAGCTACGTCTAT
TTTCTACTAGATGTTTACACAGTTTTGTCACAGGAAATCTACGCTTAAAATATGTATTTC
ATTCAAGCGGTAACCGCTGTACGAGCAGTGACATTGCTGGTCGCACCTTAAATGTAAACC
AACGTTACGGCACACCGTGATGTACCCGCATTAAAGTTTTGTAAATTCGTTATTACGATT
ATTGA

5973_130_8_-1

TGGTTTACATTTAAGGTGCGACCAGCAATGTCACTGCTCGTACAGCGGTTACCGCTTGAA
TGAAATACATATTTTAAGCGTAGATTTCCTGTGACAAAACTGTGTAAACATCTAGTAGAA
AATAGACGTA

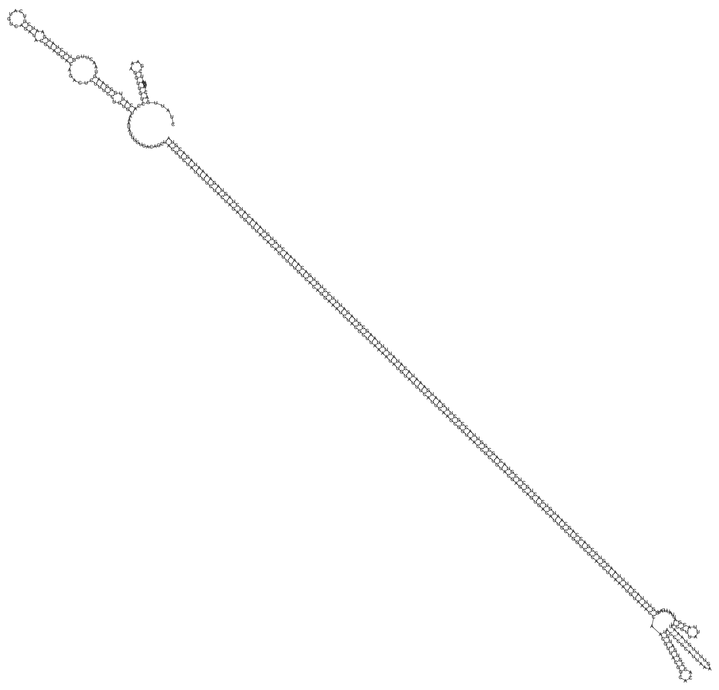

69 60609_124_4_1 . . . 60648_126_4_-1

60609_124_4_1

TTATTGACGTTTTTTTTTTCTTAGTCTCTTTAATGAAGAAGGAAAAAGAACTAAGTTATT
ATTAGAAGGAAAAGGAGGAGGGGAGGCGAGAAAGAATGGTGGAGAATAAATGAAAATCTC
CAAG

60648_126_4_-1

GTGACATGGTATAATCCATATGTATCGTGCTGAAATTTTTTCTTGGAGATTTTCATTTAT
TCTCCACCATTCTTTCTCGCCTCCCCTCCTCCTTTTCCTTCTAATAATAACTTAGTTCTT
TTTCCT

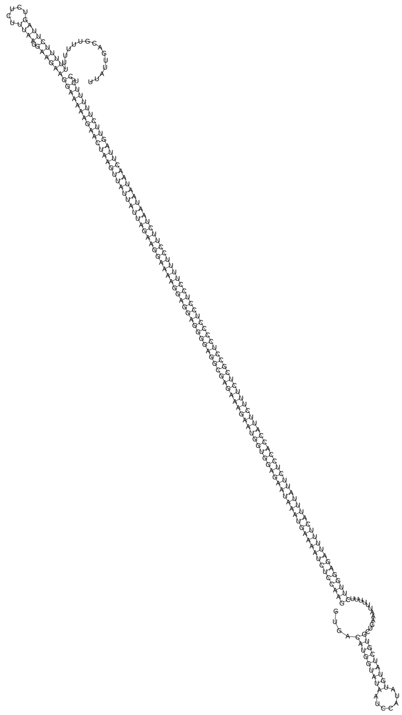

70 1330725_124_4_1 . . . 1330771_121_4_-1

1330725_124_4_1

CCAAGCCGATCTATTTTGGATATCTTATTGTCATTTTTCAAGTGAAAAAAGGTCTCCCTT
TCTATACGAGAGTTCTCATATGGAATGAGAGAATAAGCGGTAAGATATCGAACATTCACA
AGAA

1330771_121_4_-1

GATATTGTATATATTCAATTGCACCTCTTAACTGGTACTTTATTTCTTGTGAATGTTCGA
TATCTTACCGCTTATTCTCTCATTCCATATGAGAACTCTCGTATAGAAAGGGAGACCTTT
T

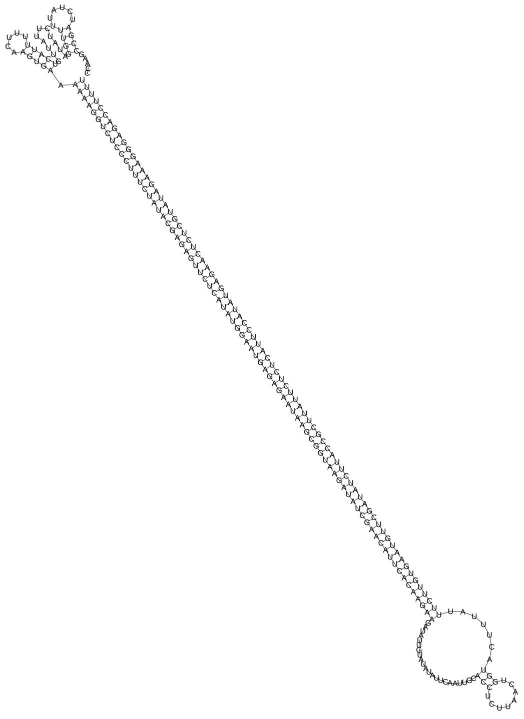

71 1516735_200_4_1 . . . 1516529_286_4_-1

1516735_200_4_1

ATAGCAGAATGTGACACAAGCTATACCATACGTCTCTGATCGACATTCATGTATGGTATC
GTCTATATCTTGTGCTTAAAGGTAGCGTATAGTAAGGTAATTACTATATGATCAATACTG
TGTGTGTAGAGTACAATAAAATATATGGGAAGAACATAAAAAGTTCGTATTGTGACATGT
TATTGAGGCTATGGTAAGCG

1516529_286_4_-1

TTTAAGCACAAGATATAGACGATACCATACATGAATGTCGATCAGAGACGTATGGTATAG
CTTGTGTCACATTCTGCTATCGTATATGGTATACATGGCACATACTTTAAATTTTAAAGT
AATGTCACTTGATTAAGATCTTTCCTATACGTCAAGGTCTCGAGATAAAACTTTATGTCT
CAAATAATACATGCTAGGCATATAATGACCCATTTTCTGGAGTTGATGTACTCAAGACAT
TGGTTAAATATCAATCACGCATGATGATAGTGCGCCCTATTATAAC

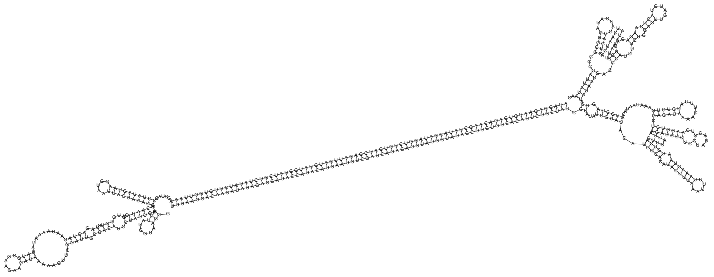

72 1517880_200_4_1 . . . 1518000_200_4_-1

1517880_200_4_1

GATATCCGCTCGAGATTAAGTGCGGCCCTCTCCACCCGATAAGGAACAAAGGGCATGCTT
CCTTTTTTAGCTTTACGCACTATTGTCAAATTGTTTATATAAACAGGCTGATATAGAATT
CATCCTGAAAACAATGGTAAAGTTTTTCTTATTGCACTTCGCTATCCTAAATTCAGGGTT
GAGATTTCTTTCTCTTTATG

1518000_200_4_-1

TTGCGTTCTTACGTCAAAGAACAATGATGCTACGGAGCGCAAGAAAGTGACGTGGCCAAC
TATCAGCGCGCTAGGATATGTATGCACTGCCGAAAGGCTGAAGCGTATCGTACATTAATT
CATAAAGAGAAAGAAATCTCAACCCTGAATTTAGGATAGCGAAGTGCAATAAGAAAAACT
TTACCATTGTTTTCAGGATG

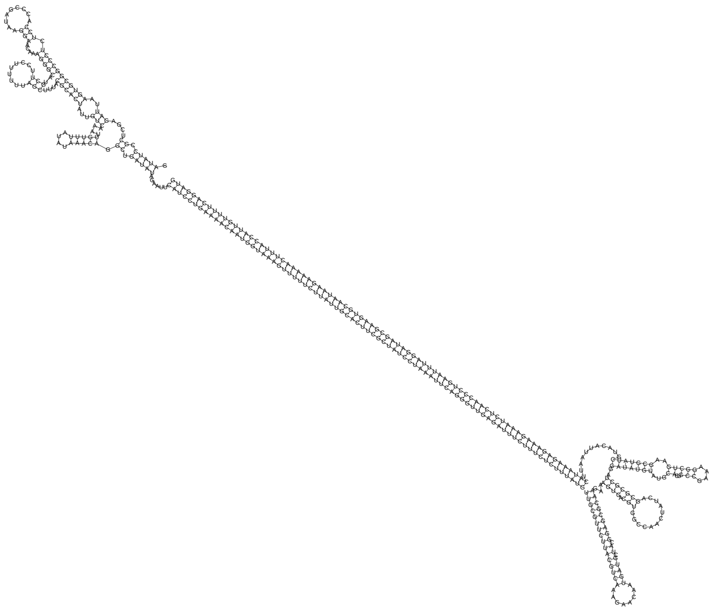

73 77832_120_5_1 . . . 77751_161_5_-1

77832_120_5_1

AAACTTTTTTCAACCTCGAAGAAAATCGTCGTCCTTCGAAGATTTAGTGGGAATCTTCAA
GGTGGATAGTTTCCAAATTAAAACTGCAGAAGAGGCTTTTAAAACATGCCTGAGGTATAA

77751_161_5_-1

TAATTTGGAAACTATCCACCTTGAAGATTCCCACTAAATCTTCGAAGGACGACGATTTTC
TTCGAGGTTGAAAAAAGTTTTTCAAGAATTTTCGCGCTTCTTCCTAATCGGGAATTTATC
TGTCATAATTATAATTCCCTATTTGGGAATTGGAACGAAAT

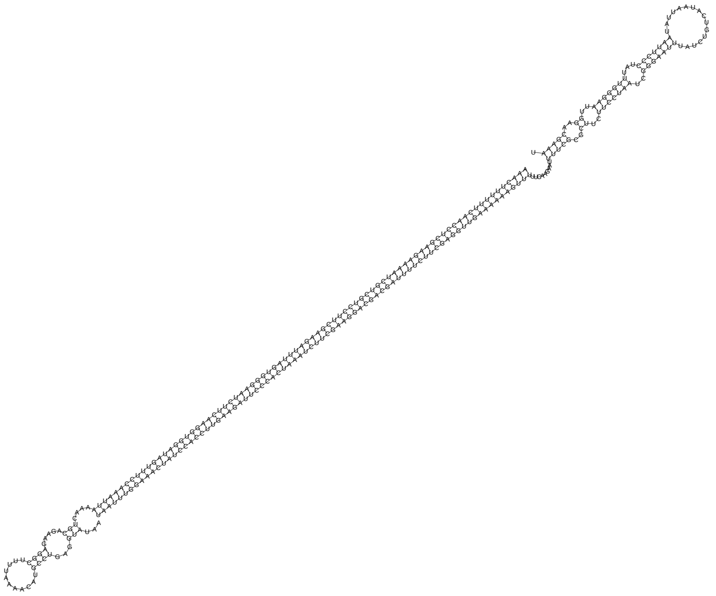

74 322006_121_5_1 . . . 322046_121_5_-1

322006_121_5_1

TAAAAAGCGTTGCGTGATGTTTGGGTGCAATTGGCGGAAAAGCGGAACTTTCAAATTTAC
GATAGGTGGAGATACATCTCCACCTTTCTCCCCATTATCTTTGAATTTTCCTCAAATATA
T

322046_121_5_-1

GATTAAACAATGGCATTTCTATGGATTTGTTGTTCTCTTTATATATTTGAGGAAAATTCA
AAGATAATGGGGAGAAAGGTGGAGATGTATCTCCACCTATCGTAAATTTGAAAGTTCCGC
T

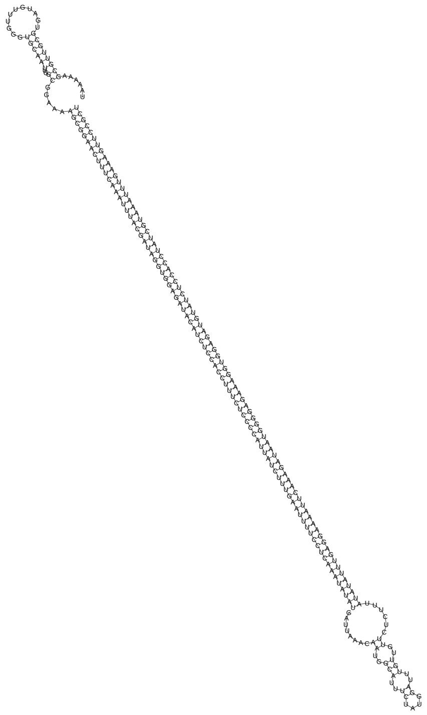

75 559126_122_5_1 . . . 559168_200_5_-1

559126_122_5_1

ATGCGATTGTCGTGTAATGGTATCGCGCATACTGAAGGTTTCAGCCCTGTTTTGATGTGC
CGCGGCAAAGTGGTTTTTCTCCATACAGGATGAAGTGTTTCTTTCCCCATGCCGCGGCAA
GC

559168_200_5_-1

CAAGAGACTATACATCCCACAATTGATTCGGTAGGTGCTTTTATACTGATTCATTTGAGG
TCTTGGCATTTGTTCAATTGAAGTACATTTAGTTGAAAAGTTGAGATTTATTTAGGATGA
GCTTGCCGCGGCATGGGGAAAGAAACACTTCATCCTGTATGGAGAAAAACCACTTTGCCG
CGGCACATCAAAACAGGGCT

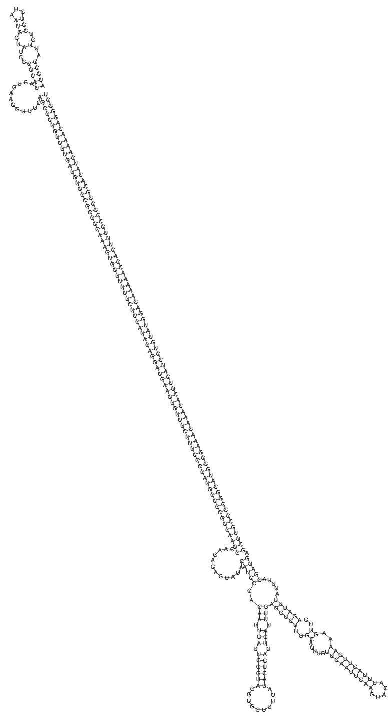

76 255408_119_6_1 . . . 255367_120_6_-1

255408_119_6_1

TTTTCTTTTGTTGGTTTTTGCAACCAGAGTATTTTTCCTGGTTGCTCCAGTAAGGTATTT
CGCTATCTTCCAACAAACGGGGCACACGTGCGGGAGTTTCAAAGGGGCAGAATAGTGGG

255367_120_6_-1

CGTTTGTTGGAAGATAGCGAAATACCTTACTGGAGCAACCAGGAAAAATACTCTGGTTGC
AAAAACCAACAAAAGAAAAAATGGAAGACCTAAGAACTATGCATTTTTTTTTAAGGGGTT

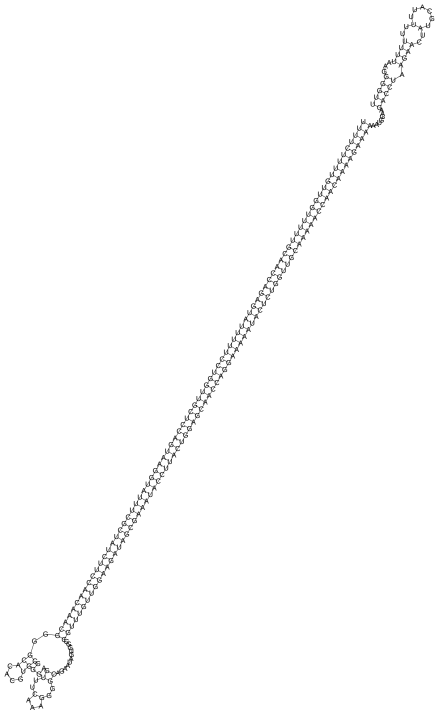

77 268349_431_6_1 . . . 268591_148_6_-1

268349_431_6_1

GATTTCTTCTCGAGTCATATATACTATTACATGAGTTCGAGTATGGTGTTGTTTTCTTTA
GAATGTCTTATCAAGACCGACAATTCATACAGGTCGAGATATGTAAAGTCTACATGCCGA
GCTAGGATGAGAAAAAAAGTATACAAATTGCAGGCAAATAAACACACCAAAAGATACTAA
AAAAATCTTTTAAAGTTTTGGTTAGTTATTGGGGATCATCATGCATTTAACGACAACAGA
GAAAAGAAAAACAAAAAAAAACAATACAATGTAAAAAGCAGTACTATGTAGGAATAAATC
CTGCCTCAATTGCAGAAAAAAAAAGGTATGCGTAATCTAAATAAGATACTATGTATTGAT
ATTTCATATCACTAACTTCTCTCAGATCAAATATGATTTAAAGGAGCGATTTCTGGACTG
TACAAAAAAAA

268591_148_6_-1

TTGATCTGAGAGAAGTTAGTGATATGAAATATCAATACATAGTATCTTATTTAGATTACG
CATACCTTTTTTTTTCTGCAATTGAGGCAGGATTTATTCCTACATAGTACTGCTTTTTAC
ATTGTATTGTTTTTTTTTGTTTTTCTTT

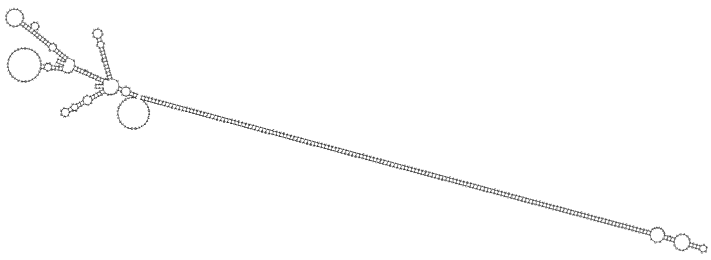

78 17016_121_7_1 . . . 16965_130_7_-1

17016_121_7_1

ACCTTGAAGTATATATATATATTTAATAATAAATACATTATTGGAATGGCTAGTAAATTTTG
ACATAAACATGATTTCCGAACGAATTAAAATACAGTTGATTTAGGAGAAAGTATCAGTAC
A

16965_130_7_-1

TCGGAAATCATGTTTATGTCAAAATTTACTAGCCATTCCAATAATGTATTTATTATTAAA
TATATATATACTTCAAGGTCTGATGGGGGTGCTGTAGCATTTGCGATGTAGCGATAGCAA
TTGAAAATAA

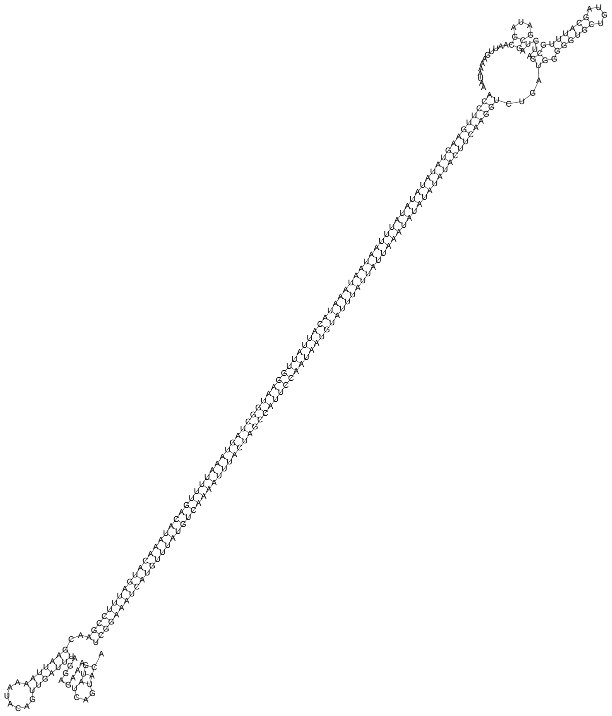

79 166622_123_7_1 . . . 166663_122_7_-1

166622_123_7_1

TGTATGCCCTTTTCATGTATAAAAGAAGGGTAATTTAAAAAAGTTTATAATCATCCAGAT
TCCTTATTTTTTTTCTTCTTTCGCCAGGTGTTTTACCCAGCTAAGACTAACCTTGTGGTT
CAG

166663_122_7_-1

ATTTCAGAATTGTAATATCGGACGGCAATACTAGTGTAATCTGAACCACAAGGTTAGTCT
TAGCTGGGTAAAACACCTGGCGAAAGAAGAAAAAAAATAAGGAATCTGGATGATTATAAA
CT

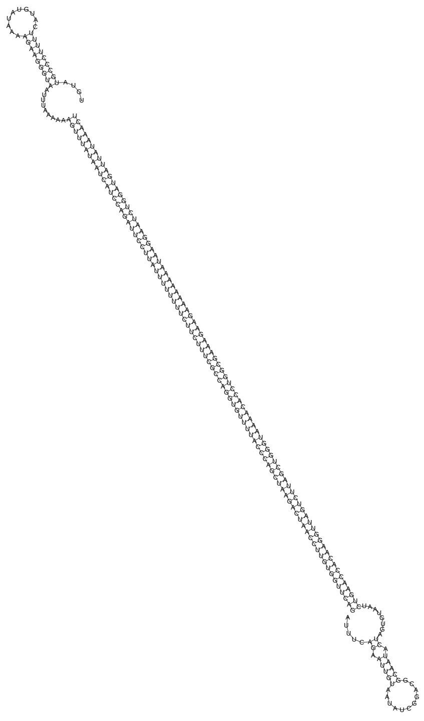

80 272298_67_7_1 . . . 272298_67_7_1

272298_67_7_1

ATATATATATATATATATATATATGTTTGTATGTATATATATATATACGTATATATATCATA
TATACGA

272298_67_7_1

ATATATATATATATATATATATATGTTTGTATGTATATATATATATACGTATATATATCATA
TATACGA

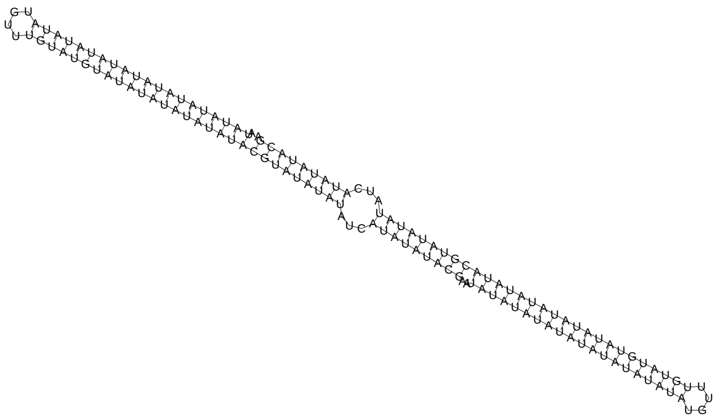

81 272298_67_7_1 . . . 464799_78_11_1

272298_67_7_1

ATATATATATATATATATATATATGTTTGTATGTATATATATATATACGTATATATATCATA
TATACGA

464799_78_11_1

TCTTGCCGACGGCTACGTATATATATATATATATATAAATATATATATATATATTCATGT
ATACTGTATATGTACAAT

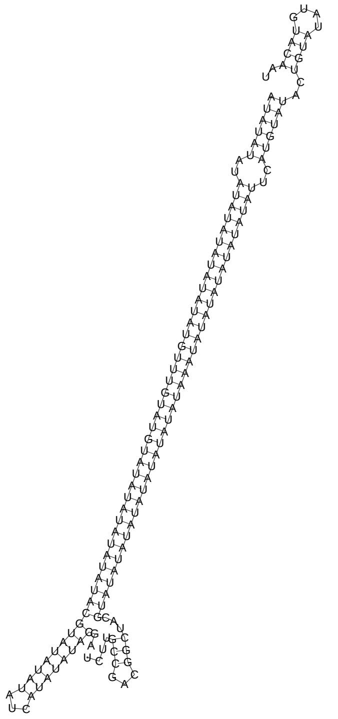

82 884226_119_7_1 ... 884266_119_7_-1

884226_119_7_1

AATATTTTGGTGCTGGGATTCTTTTTTTTTCTGGATGCCAGCTTAAAAAGCGGGCTCCAT
TATATTTAGTGGATGCCAGGAATAAACTGTTCACCCAGACACCTACGATGTTATATATT

884266_119_7_-1

GTAACCCGTACATGCCCAAAATAGGGGGCGGGTTACACAGAATATATAACATCGTAGGTG
TCTGGGTGAACAGTTTATTCCTGGCATCCACTAAATATAATGGAGCCCGCTTTTTAAGC

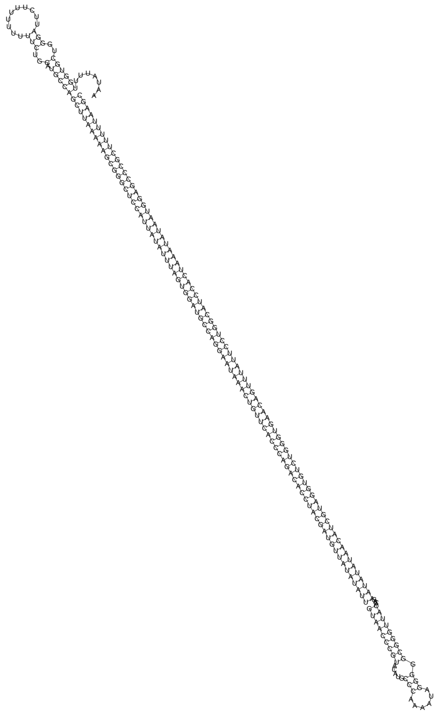

83 50743_119_8_1 . . . 50784_119_8_-1

50743_119_8_1

AATAATCTAAATACCGCGTTCGTGCGAGACATTCTCTGTGATTCACAGATTACGGCGCAG
AAAATAAAATAGCCGTCCAGCTCGGAGTTTCTAAAATTACGTCCGAGATGACGGAGGCT

50784_119_8_-1

TCGTAGATTGGCATCCCTCACCCGTCTGAAATGATTTTATAAGCCTCCGTCATCTCGGAC
GTAATTTTAGAAACTCCGAGCTGGACGGCTATTTTATTTTCTGCGCCGTAATCTGTGAA

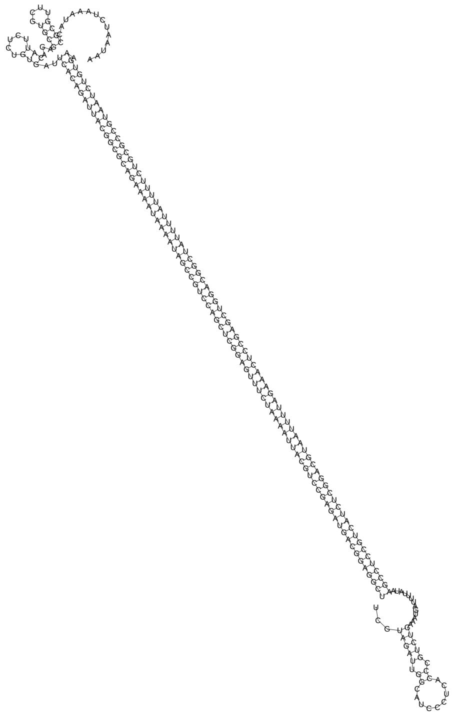

84 **236597_120_8_1 . . . 236637_122_8_-1**

236597_120_8_1

TAAAAGTATTTTAGTTTGTTGTTGTAATTTCGTCTTTTCTCTCACTACCGTAACAACGTG
GTAGTTGGCAGAATATATATATTCTTGGCAACCTAGTTGTTACTCAAATAGAAGAAAAAG

236637_122_8_-1

TGCGAGTGAATAGGGAGAACCAAATAAATAGGGAGAAACTTGCTTTTTCTTCTATTTGAG
TAACAACTAGGTTGCCAAGAATATATATATTCTGCCAACTACCACGTTGTTACGGTAGTG
AG

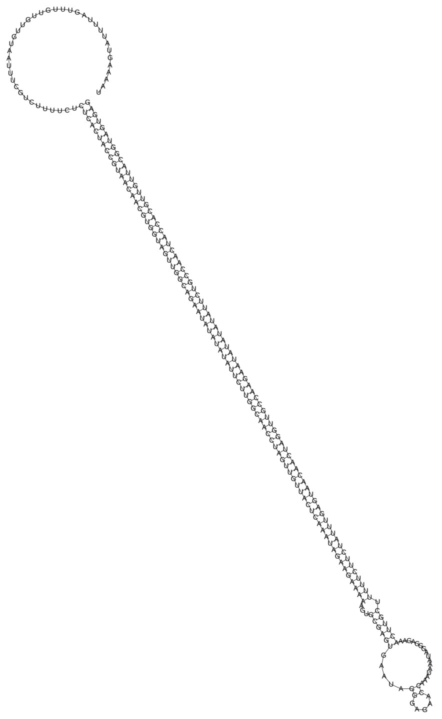

85 319950_120_8_1 . . . 319990_120_8_-1

319950_120_8_1

CAAGGTTAGCGCTTTACGGACTTTATTGCTATTCTAGTTCAAAGAAAGCAAATGGCGCAG
TTACCAAACCTCGCCATTTGTTTTTTCCGTTATTTTTTCTTCTTATAAACGCGCCTAGAA

319990_120_8_-1

TTAGCGCTTTCTCAGGATTTGCGCTTCGCACGGTTTTTTCTTCTAGGCGCGTTTATAAGA
AGAAAAAAATAACGGAAAAAACAAATGGCGAGGTTTGGTAACTGCGCCATTTGCTTTCTTT

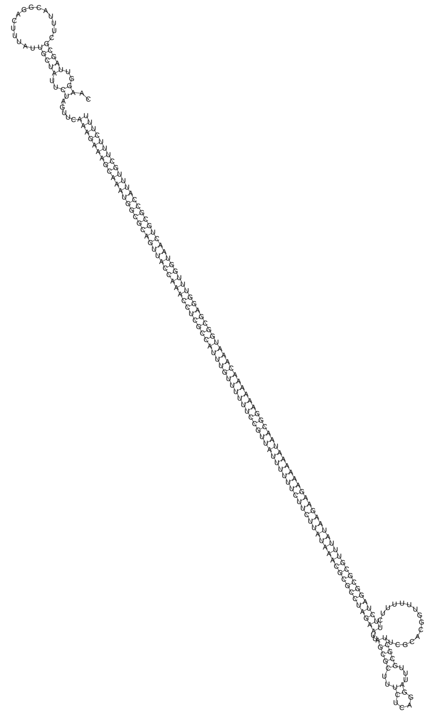

86 117720_237_9_1 . . . 117760_117_9_-1

117720_237_9_1

GCCAGCTATATTTCCATATTCCTTAGTTAAAGCTCATCGCAAAATTTTTCAAAGAGCATT
AGGGTCACGTGTATACATTCACGTGACTATCTAGTACCCGCCCCGTGGCAAAAAAAGAAG
ACACCAATTTGGCACTGAGAAAAGAAAAAGTTTATCAATAACGAAAACGAAACTTAAGAG
GAAAAAGAGTATAGAGAAAAACAAAGGCAATCAGCGGCTAATTTGGTGGAAATAACA

117760_117_9_-1

TGATAAACTTTTTCTTTTCTCAGTGCCAAATTGGTGTCTTCTTTTTTTGCCACGGGGCGG
GTACTAGATAGTCACGTGAATGTATACACGTGACCCTAATGCTCTTTGAAAAATTTT

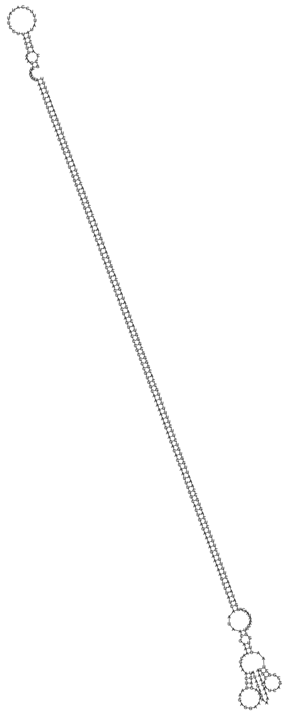

87 12506_86_0_1 . . . 32419_121_0_1

12506_86_0_1

TATAGTGGTGGGGTCCCAATTATTATTTTCAATAATAATTTATCATGGGACCCGGATATC
TTCTTGTTTTTATTTATTATTTTATT

32419_121_0_1

AATAAAATAATAAATGATAAACAAGAAGATATCCGGGTCCCAATAATAATTATTATTGAA
AATAATAATTGGGACCCCATATAGAATATAAATAATTAAATATATATATATATAAATAATAA
T

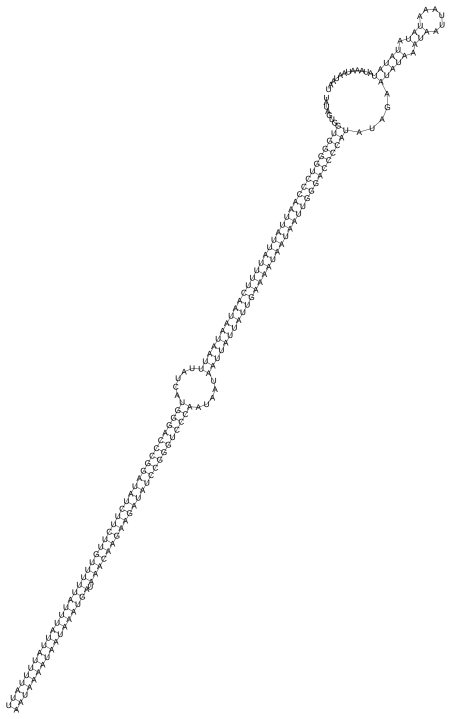

88 12506_86_0_1 . . . 45142_123_0_1

12506_86_0_1

TATAGTGGTGGGGTCCCAATTATTATTTTCAATAATAATTTATCATGGGACCCGGATATC
TTCTTGTTTTTATTTATTATTTTATT

45142_123_0_1

TAAATAATATAATAAATGATAAACAAGAAGATATCCGGGTCCCAATAATAATTATTATTG
AAAATAATAATTGGGACCCCCATCTAAAATATATATATAACTAATAATATATTATATATA
TTA

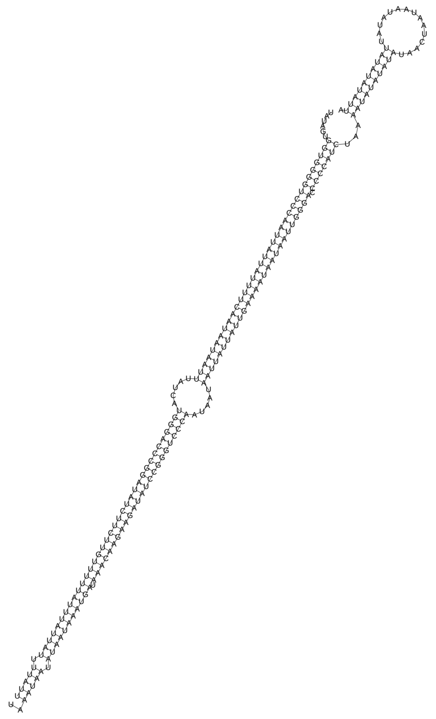

89 29726_260_0_1 . . . 29783_136_0_-1

29726_260_0_1

TATAGTATTAAAAAAAAATAAAATATTTAATAAATATTATTATTAATAATATTTATTAAAA
ATAATATAACATAATAAATATAAGATTATTATATAATATATTTATTATATCATATAGTTC
CGGGGCCCGGCCACGGGAGCCGGAACCCCGGAAGGAGAAATTATAACATATTTTTTAATA
ATATTCATATTTATTTTATATACAAATAAATATATTTATTTAGAATAATAAAAAAAAATA
ATAAATAAATATATTATTAT

29783_136_0_-1

TAAATATGAATATTATTAAAAAATATGTTATAATTTCTCCTTCCGGGGTTCCGGCTCCCG
TGGCCGGGCCCCGGAACTATATGATATAATAAATATATTATATAATAATCTTATATTTAT
TATGTTATATTATTTT

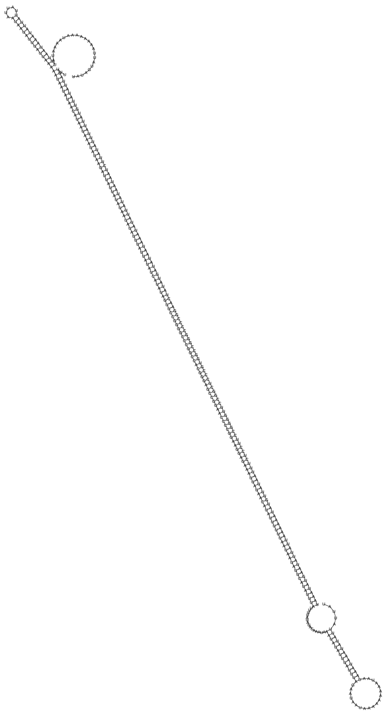

90 29726_260_0_1 . . . 34913_97_0_-1

29726_260_0_1

TATAGTATTAAAAAAAAATAAAATATTTAATAAATATTATTATTAATAATATTTATTAAAA
ATAATATAACATAATAAATATAAGATTATTATATAATATATTTATTATATCATATAGTTC
CGGGGCCCGGCCACGGGAGCCGGAACCCCGGAAGGAGAAATTATAACATATTTTTTAATA
ATATTCATATTTATTTTATATACAAATAAATATATTTATTTAGAATAATAAAAAAAAATA
ATAAATAAATATATTATTAT

34913_97_0_-1

TTTATTAATATAATTTATATATTTATCTTATTCCTCCTTTCGGGGTTCCGGCTCCCGTGG
CCGGGCCCCGGAACTATTAATATATAAATTGAATTAT

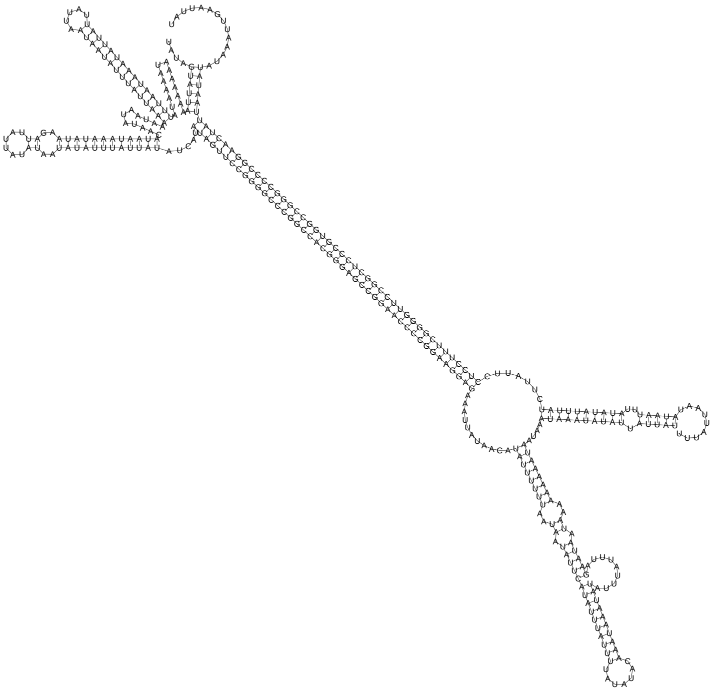

91 32419_121_0_1 . . . 12506_86_0_1

32419_121_0_1

AATAAAATAATAAATGATAAACAAGAAGATATCCGGGTCCCAATAATAATTATTATTGAA
AATAATAATTGGGACCCCATATAGAATATAAATAATTAAATATATATATATATAAATAATAA
T

12506_86_0_1

TATAGTGGTGGGGTCCCAATTATTATTTTCAATAATAATTTATCATGGGACCCGGATATC
TTCTTGTTTTTATTTATTATTTTATT

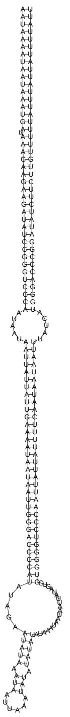

92 32419_121_0_1 . . . 30507_136_0_-1

32419_121_0_1

AATAAAATAATAAATGATAAACAAGAAGATATCCGGGTCCCAATAATAATTATTATTGAA
AATAATAATTGGGACCCCATATAGAATATAAATAATTAAATATATATATATAAATAATAA
T

30507_136_0_-1

TATTTCTATTTTATATACATTATTATTATATTAATTAATATGATATTATAATGGTGGGGG
GTCCCAATTATTATTGAAAATAATAATTATTAATGGGACCCCAGATATCTTCTTGTTAAT
CATTTATTATTTTATT

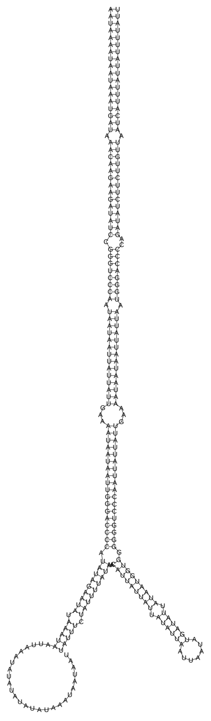

93 32419_121_0_1 . . . 54498_123_0_1

32419_121_0_1

AATAAAATAATAAATGATAAACAAGAAGATATCCGGGTCCCAATAATAATTATTATTGAA
AATAATAATTGGGACCCCATATAGAATATAAATAATTAAATATATATATATATAAATAATAA
T

54498_123_0_1

ATTAATAATATATATATTATTTTATATATTTTATTTAATATAAATTATTTATATTTTTAT
ATTTTATTATGAGGGGGGGTCCCAATTATTATTTTCAATAATAATTTATCATGGGACCCG
GAT

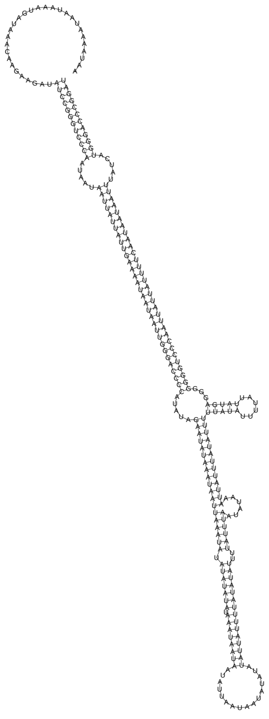

94 45142_123_0_1 ... 12506_86_0_1

45142_123_0_1

TAAATAATATAATAAATGATAAACAAGAAGATATCCGGGTCCCAATAATAATTATTATTG
AAAATAATAATTGGGACCCCCATCTAAAATATATATATAACTAATAATATATTATATATA
TTA

12506_86_0_1

TATAGTGGTGGGGTCCCAATTATTATTTTCAATAATAATTTATCATGGGACCCGGATATC
TTCTTGTTTTTATTTATTATTTTATT

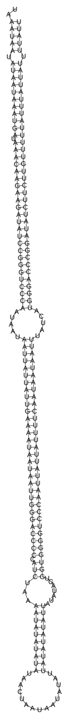

95 45142_123_0_1 . . . 30507_136_0_-1

45142_123_0_1

TAAATAATATAATAAATGATAAACAAGAAGATATCCGGGTCCCAATAATAATTATTATTG
AAAATAATAATTGGGACCCCCATCTAAAATATATATATAACTAATAATATATTATATATA
TTA

30507_136_0_-1

TATTTCTATTTTATATACATTATTATTATATTAATTAATATGATATTATAATGGTGGGGG
GTCCCAATTATTATTGAAAATAATAATTATTAATGGGACCCCAGATATCTTCTTGTTAAT
CATTTATTATTTTATT

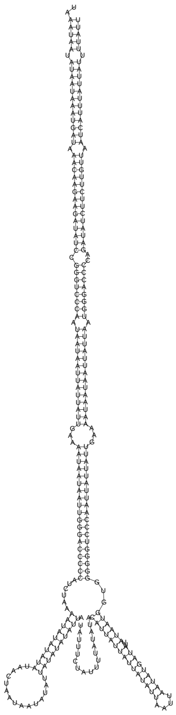

96 45142_123_0_1 . . . 54498_123_0_1

45142_123_0_1

TAAATAATATAATAAATGATAAACAAGAAGATATCCGGGTCCCAATAATAATTATTATTG
AAAATAATAATTGGGACCCCCATCTAAAATATATATATAACTAATAATATATTATATATA
TTA

54498_123_0_1

ATTAATAATATATATATTATTTTATATATTTTATTTAATATAAATTATTTATATTTTTAT
ATTTTATTATGAGGGGGGGTCCCAATTATTATTTTCAATAATAATTTATCATGGGACCCG
GAT

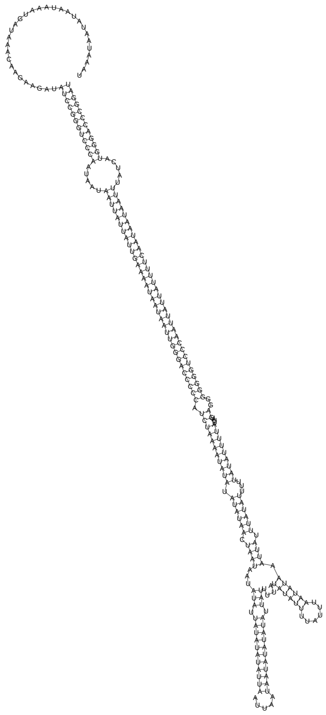

97 54498_123_0_1 . . . 32419_121_0_1

54498_123_0_1

ATTAATAATATATATATTATTTTATATATTTTATTTAATATAAATTATTTATATTTTTAT
ATTTTATTATGAGGGGGGGTCCCAATTATTATTTTCAATAATAATTTATCATGGGACCCG
GAT

32419_121_0_1

AATAAAATAATAAATGATAAACAAGAAGATATCCGGGTCCCAATAATAATTATTATTGAA
AATAATAATTGGGACCCCATATAGAATATAAATAATTAAATATATATATATAAATAATAA
T

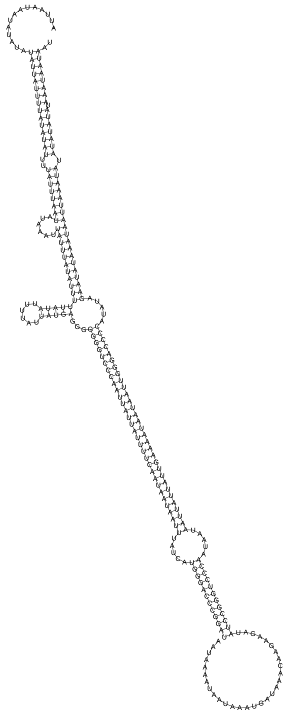

98 54498_123_0_1 ... 45142_123_0_1

54498_123_0_1

ATTAATAATATATATATTATTTTATATATTTTATTTAATATAAATTATTTATATTTTTAT
ATTTTATTATGAGGGGGGGTCCCAATTATTATTTTCAATAATAATTTATCATGGGACCCG
GAT

45142_123_0_1

TAAATAATATAATAAATGATAAACAAGAAGATATCCGGGTCCCAATAATAATTATTATTG
AAAATAATAATTGGGACCCCCATCTAAAATATATATATAACTAATAATATATTATATATA
TTA

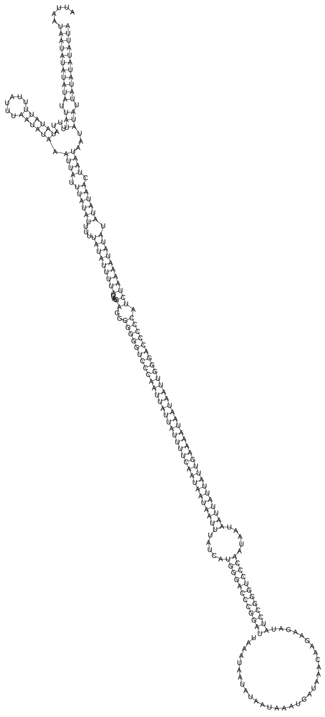

99 62832_137_0_1 . . . 34913_97_0_-1

62832_137_0_1

ATATATTATTAATTTAATAATTATTATAATAGTTCCGGGGCCCGGCCACGGGAGCCGGAA
CCCCGAAAGGAGTTTATAAAAGATATATTTTTATATTATATTATATTATATTTAATAAAT
ATTACCTTTTTTTATTA

34913_97_0_-1

TTTATTAATATAATTTATATATTTATCTTATTCCTCCTTTCGGGGTTCCGGCTCCCGTGG
CCGGGCCCCGGAACTATTAATATATATAAATTGAATTAT

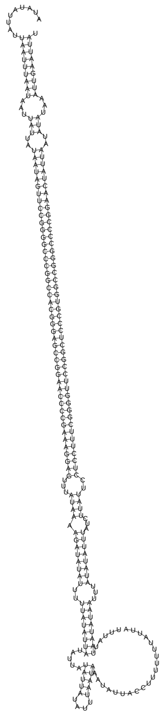

100 62832_137_0_1 ...29783_136_0_-1

62832_137_0_1

ATATATTATTAATTTAATAATTATTATAATAGTTCCGGGGCCCGGCCACGGGAGCCGGAA
CCCCGAAAGGAGTTTATAAAAGATATATTTTTATATTATATTATATTATATTTAATAAAT
ATTACCTTTTTTTATTA

29783_136_0_-1

TAAATATGAATATTATTAAAAAAATATGTTATAATTTCTCCTTCCGGGGTTCCGGCTCCCG
TGGCCGGGCCCCGGAACTATATGATATAATAAATATATTATATAATAATCTTATATTTAT
TATGTTATATTATTTT

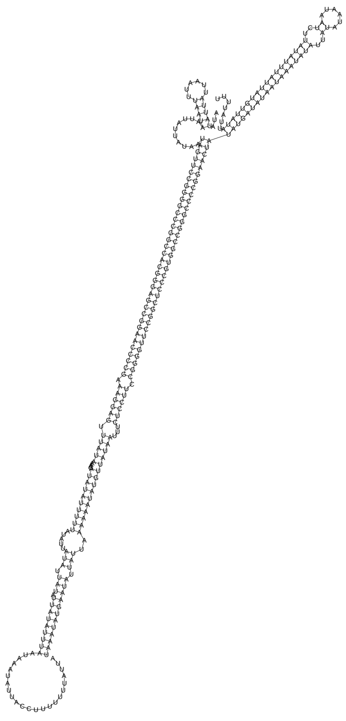

101 62832_137_0_1 . . . 2096_297_0_-1

62832_137_0_1

ATATATTATTAATTTAATAATTATTATAATAGTTCCGGGGCCCGGCCACGGGAGCCGGAA
CCCCGAAAGGAGTTTATAAAAGATATATTTTTATATTATATTATATTATATTTAATAAAT
ATTACCTTTTTTTATTA

2096_297_0_-1

TCTATAAAGAAATGAGATTAGATGATATTGAAATGAAATAAAATGAGATGAGATGAATTG
GGGCCCGGAAAGGGACCGAACCCCTCATAATAATTAATTTAATATATATTATAATAAAAC
TTATTATATATATTTTTATATATATATATATATTTTTTTTATATATTATATAATAAATAT
TACTTCTTATTAAAATTCCTTATTTTTATTATTTTTAATAATTAAATTCATATAGTTATT
ATAATATATAGATAAACTCCTTTCGGGGTTCCGGCTCCCGTGGCCGGGCCCCGGAAC

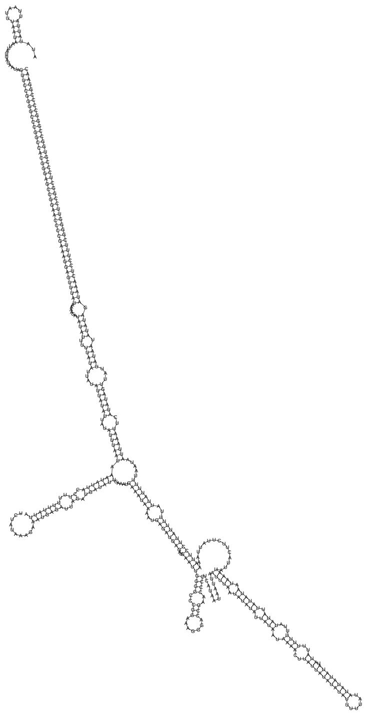

102 76206_186_0_1 ... 29783_136_0_-1

76206_186_0_1

TATATTTATAAAAATATTAATATTTTATTTAAAATAAATAATGATTAATTTATAAAATAT
ATATTAATTAAGTTTCGGGTCCCGGCTACGGGACCCGGAACCCCCGAGAGGAGTTATTAT
ATTTATAATTAAATCTTTAAATAATATATCTTAAATTATTATATTGATATTAATATTATA
TTGATA

29783_136_0_-1

TAAATATGAATATTATTAAAAAATATGTTATAATTTCTCCTTCCGGGGTTCCGGCTCCCG
TGGCCGGGCCCCGGAACTATATGATATAATAAATATATTATATAATAATCTTATATTTAT
TATGTTATATTATTTT

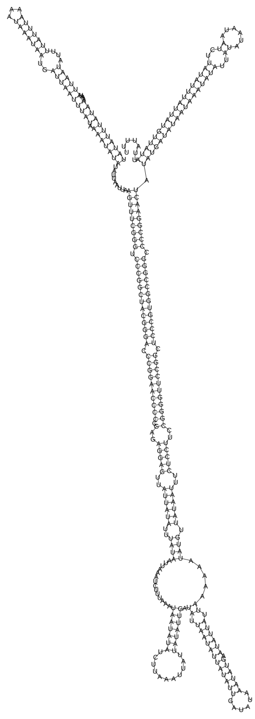

Supplement: Additional file 10 — Analysis of potential duplexes formed by predicted intergenic ncRNA transcripts. First, we filtered potential duplexes by fast searches for overlap regions with wublast (Gish, W., personal communication) with parameters that also allow for G-U basepairs, as described in Steigele et al [3]. Second, the thermodynamically preferred duplex between two predicted RNA molecules was calculated by RNAcofold. In most cases, only very large overlaps between predicted RNA molecules were found. [file 1741-7007-5-25-S10.pdf]
